# A Serious Game (MyDiabetic) to Support Children’s Education in Type 1 Diabetes Mellitus: Iterative Participatory Co-Design and Feasibility Study

**DOI:** 10.2196/49478

**Published:** 2024-05-07

**Authors:** Daniel Novak

**Affiliations:** 1 Department of Cybernetics Faculty of Electrical Engineering Czech Technical University in Prague Prague Czech Republic

**Keywords:** diabetes mellitus, serious games, mobile app, co-design, user-centered design, serious game, gaming, diabetes, child with diabetes, child, children, insulin, glucometer, glucose, patient education, insulin, mobile phone

## Abstract

**Background:**

Serious games, which are gaming applications used for purposes beyond entertainment to educate users on, and address, specific issues, may present a timely approach to promote healthy diabetes management behaviors among children with type 1 diabetes mellitus (T1DM). The lasting benefits associated with these serious games encompass improved patient education; enhanced glycemic control; the reinforcement of bonds within the community of people with diabetes; the facilitation of meaningful dialogues with caregivers, especially within the familial setting; and a significant reduction in the economic burdens associated with subsequent complications.

**Objective:**

This paper primarily aims to provide a detailed overview of the iterative design process and the associated evaluation methods used in the development of the educational game. Furthermore, this study aims to enhance motivation for sustained and extended engagement with the game over time. The MyDiabetic game design aims to educate children on various aspects, including the connections among food, insulin, and physical activity. Furthermore, it seeks to impart knowledge related to the operation of a glucometer and an insulin pen, as well as more advanced technologies such as administering glucagon, measuring ketoacidosis, and continuous glucose monitoring.

**Methods:**

The co-design methodology was applied, involving interviews, design workshops, and prototype feedback sessions. A combination of several approaches, such as tailoring, observational learning, social and family support, decision-making practice, and reward systems, was used to support children’s compliance. Moreover, incorporating the literature, guidelines, and current practices into the design ensured that the game was aligned with established health care pathways and included relevant information and best practices for diabetes management.

**Results:**

The game was tested on 32 children in 3 iterations. Positive responses were received from children who tested the game as well as their parents. The game was also presented to 5 schoolmates of children with T1DM who appreciated a better understanding of the disease and the opportunity to support their friends more efficiently in T1DM compensation. The involvement of children and clinicians in participatory co-design contributed to to the game's high acceptance. With regard to the game’s impact on education, 1 week of testing revealed an enhancement in educational outcomes.

**Conclusions:**

The game is especially suitable for children newly diagnosed with T1DM because it acquaints them in a fun way with new terminology; for example, they can try to measure glycemia levels in an interactive way. The game also caters to children who still need to develop reading skills by including an audio guide. The guide ensures that children of all literacy levels can benefit from the game’s educational content and interactive experiences. The game is available for download on Google Play and the Apple App Store.

## Introduction

### Background

Type 1 diabetes mellitus (T1DM) is the most common form of diabetes in children. Poor blood glucose control increases the risk of chronic microvascular complications, including renal and retinal complications. When T1DM starts in childhood or young adulthood, the course of the disease is long, and metabolic control is essential to prevent such complications [1]. Advances in the treatment of T1DM have decreased the risk of complications and delayed their occurrence, with a resultant overall increase in the quality of life of patients. Nutritional education, with systematic assessments of carbohydrate intake and the use of the insulin-to-carbohydrate ratio, has allowed for optimizing insulin dosage [2]. The basal-bolus scheme with multiple-dose insulin injections, continuous subcutaneous insulin infusion, and multiple capillary blood glucose measurements allow for better metabolic control. For this, adequate and continued diabetes education for patients and families is required [3].

In general, mobile phones are a natural choice for use in increasing the efficiency of medical care and patient motivation [4,5]. The development of monitoring technologies and designing systems for improving diabetes mellitus compensation based on a mobile platform is increasing and widespread [6,7]. In the field of diabetes mellitus, there are already several pilot studies that validate the effectiveness of this new technology [1,8-10]. The biggest problem so far is the motivation of patients, as many stop cooperating after a few weeks or months. Furthermore, some authors state that the noncooperation of patients is one of the most common reasons why we encounter failure in education outcomes [4,11,12].

Well-designed serious games can improve children’s learning, skills development, attitudes, emotions, motivation, and many other factors that encourage children to work together with family members and health professionals on treatment [13]. The long-term benefits of the serious game are improved patient education; better diabetes compensation; increased connections with the community of people with diabetes; stimulation of discussion with caregivers, especially within the family; and a significant reduction in the economic costs of subsequent complications. Furthermore, due to the widespread use of technology among children, using serious games to educate and support health behavior for children with diabetes self-management is an emerging and promising practice [14,15].

Some examples of previous approaches are Packy & Marlon (Super Nintendo) [16], Balance [17], Mario Brothers [18], Monster Manor (Nintendo) [19], and mySugr Junior [20]. Carb Counting with Lenny [21] is geared toward teaching users valuable information about healthy food choices and allowing them to apply that knowledge during gameplay. Jerry the Bear [22] seeks to educate children about T1DM by getting them to take care of the game’s avatar, check the avatar’s blood glucose level, manage insulin dosage by administering the doses using a pen or a pump, and feed the avatar with various food items. Except in the case of Jerry the Bear, the games show only the relationship among food, blood glucose level and physical activity. Some diabetes management games tested in small populations have never been brought to market and have no scalability. In addition, no studies were identified examining T1DM educational games’ impact on user behavior, knowledge, or clinical indicators. Further research is needed to better understand the sustainability of T1DM gaming as a tool for promoting adherence and the effect of education.

### Objectives

This paper primarily aims to provide a thorough overview of the iterative design process and the accompanying evaluation methods applied in the development of the educational game, MyDiabetic. Furthermore, this study aims to enhance motivation for sustained and prolonged engagement with the game.

MyDiabetic aims to not only teach children the relationship among food, insulin, and physical activity but also pass on knowledge related to working with a glucometer and an insulin pen, as well as more advanced technology such as continuous glucose monitoring (CGM), insulin pump, glucagon administration, and ketones urine test. The game is designed for children aged between 5 and 12 years. It is especially suitable for children newly diagnosed with T1DM because it acquaints them in a fun way with new terminology; for example, they can try to measure glycemia levels in an interactive way.

## Methods

### Overview

**Figure 1:**
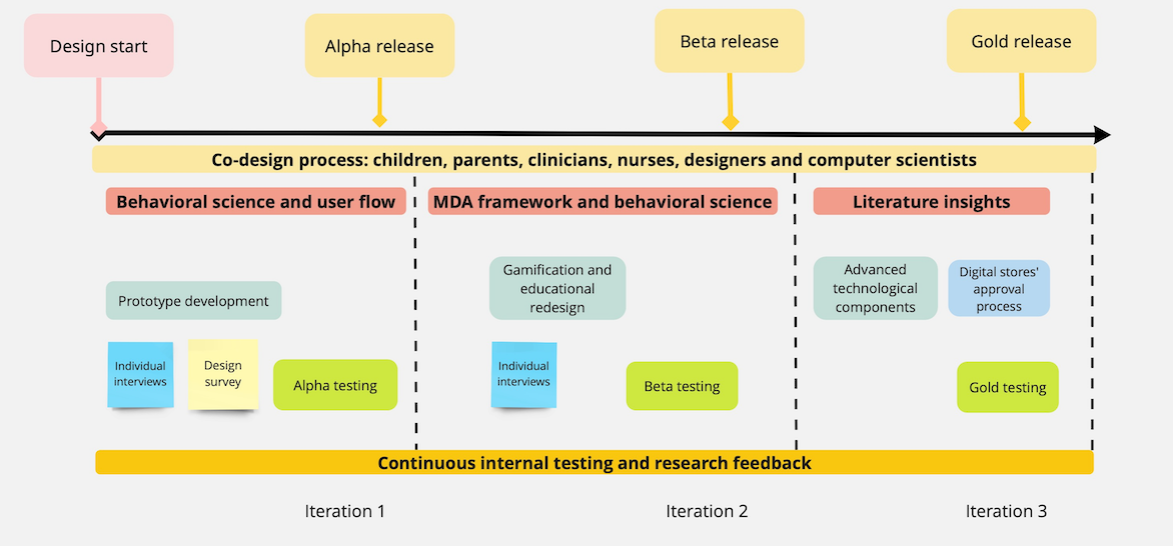
Approximate timeline for the MyDiabetic project showing the principal elements of the participatory co-design methodology along with the predominant principles used in each iteration indicated by the red bars. MDA: mechanics, dynamics, and aesthetics.

A participatory iterative co-design approach was adopted. Participatory design in each iteration was guided by the fundamental principles from both traditional game design elements (eg, user flow and the mechanics, dynamics, and framework approach) and behavioral theory tailored for diabetes support. Figure 1 illustrates an approximate timeline for the MyDiabetic project, depicting key components of the participatory co-design methodology, along with the predominant principles used in each iteration indicated by the red bars.

### Participatory Iterative Co-Design

The game was continually developed, resulting in the 3 iterations of the co-design phases applying participatory co-design principles. Figure 1 presents the timeline of the co-design process. Co-design process is intrinsically incorporated into the research. Co-design is a research methodology that actively engages diverse stakeholders to initiate, create, and validate solutions by adding creative and participative principles and tools [23]. Participatory co-design establishes that the necessary features of person-centered design, clinical acceptability, and health IT feasibility are accounted for, with each process needed for the ultimate success of the serious game. Table 1 describes user statistics during the co-design process.

The design team included several types of participants:

- Children in the user group and their parents, who were recruited through collaborating institutions, such as patient organizations, or Facebook groups
- Researchers with backgrounds in behavior change, informatics, and design
- Designers and developers (external and from the in-house IT system development group)
- Clinicians with vast expertise in T1DM management and nutrition nurses who are part of the project’s advisory group

The first iteration of the MyDiabetic game was based on qualitative and quantitative research with children with T1DM diabetes, which led to the requirement for a game to help educate these children and help them cope with their new diagnosis. The outcome of the co-design research was the concept of continuous care of an avatar (based on the Tamagotchi principle) that is represented as a 3D avatar, including technical modules illustrating glucometer and insulin pen use. Subsequently, the co-design of the second iteration focused on incorporating educational material, such as an educational library and audio guidance provided by a physician’s avatar, as well as developing other gamification features, such as game levels, minigames, and storyline extension. In addition, a model for measuring blood glucose levels was added. The third iteration addressed new technologies and methods, such as CGM and insulin pump modules, glucagon administration, and an explanation of ketoacidosis. Each iteration ended with usability testing. We followed software release cycle terminology. The alpha usability testing was focused on the evaluation of the main game concept, while the beta usability testing was the most detailed, concentrating on educational and adherence aspects. The gold usability testing evaluated new technological components only. The depiction of screens can be seen in Figure S1 in Multimedia Appendix 1.

Detailed information regarding the characteristics of the children for each iteration can be found in Tables S1-S6, S9-S10, and S12-S13 in Multimedia Appendix 2.

The clinical team was composed of experts who possessed diverse experience in various aspects of T1DM. Throughout the development process, these clinicians consistently provided invaluable feedback during presentation workshops conducted at the conclusion of each iteration.

**Table 1:** Description of iterative design and user statistics.

Iteration and activity		Users	Age (years), mean (SD)	T1DM^a^ duration (years), mean (SD)	Stage of development	Features introduced			
**Iteration 1**							Basic gaming concept, design of control elements, taking care of the avatar concept, carbohydrate counting, insulin pen administration, measurement of glycemia level, and performing exercise		
	Interviews	Boys: 4; girls: 0	12.8 (1.3)	5.5 (2.6)	Mock-up				
	Design survey	Boys: 15; girls: 12	10.2 (2.1)	4.8 (3.0)	Mock-up				
	Alpha testing	Boys: 8; girls: 4^b^	10.5 (2.7)	3.7 (2.2)	Alpha release				
**Iteration 2**									Education elements, such as an educational library or simulations of blood glucose, minigames, storyline, and levels design
	Beta testing	Boys: 2; girls: 4	9.6 (4.5)	4.8 (2.9)	Beta release				
**Iteration 3**							Advanced topics such as CGM^c^ and insulin pump illustration, measurement of ketoacidosis, and glucagon administration		
	CGM testing	Boys: 7; girls: 2	12.8 (1.7)	7.0 (3.7)	Gold release				
	Glucagon testing	Boys: 4; girls: 4	13.2 (2.6)	6.4 (1.8)	Gold release				
	Keto-acidosis testing	Boys: 2; girls: 5	12.2 (2.7)	8.9 (3.2)	Gold release				

^a^T1DM: type 1 diabetes mellitus.

^b^Of the 12 users, 5 (42%; n=2, 40% boys and n=3, 60% girls) did not have diabetes.

^c^CGM: continuous glucose monitoring.

### Serious Games and Behavioral Theory Foundations

Methodological aspects of user flow [24]; the mechanics, dynamics, and aesthetics framework [25]; and behavioral theory (tailoring [26,27], observational learning [27-29], decision-making practice [13], social and family support [30,31], and reward systems [27,32]) were used during the design of MyDiabetic. Theoretical backgrounds are summarized in the Theoretical Background and Game Design section in Multimedia Appendix 1.

On the basis of literature research, guidelines, and current practices [13,19,21,22,33], the characteristics that are important for a successful serious game were further identified (Textbox 1).

**Characteristics that are important for a successful serious game.**

**Important characteristics**

1. The main avatar should be empathetically connected to the player. One possibility is to use avatars that reflect the player in the game [34]. Customizing the avatar in game should also be possible (refer to the Avatar Tailoring subsection under Theoretical Background and Game Design and Figure S2A in Multimedia Appendix 1).
2. The game should have an incentive and customizable reward system that supports the player’s learning. As a reward, the game can offer trophies or new *unlocked* game content (refer to the Level Progress Design subsection under Theoretical Background and Game Design and Figure S2B in Multimedia Appendix 1).
3. The game should aim to develop the player’s skills by setting clear but challenging goals related to changing the player’s behavior in real life (mainly all educational and technical features developed in iterations 1-3: iteration 1 [main game concept], iteration 2 [educational and gamification features], and iteration 3 [advanced features]).
4. The difficulty should gradually increase and adapt to gradually improving the player’s skills, giving the player further opportunities for improvement (refer to the Level Progress Design subsection under Theoretical Background and Game Design in Multimedia Appendix 1).
5. The game should have a realistic and health-related story. This attractive design includes high-quality graphics, sounds, and animations to immerse the player in the game (refer to the Scenes Description subsection under Theoretical Background and Game Design and Figure S1 in Multimedia Appendix 1).

A summary of the game and the methodology is provided in Multimedia Appendix 3. In addition, Multimedia Appendix 4 contains a video highlighting the game’s main features.

### Participant Recruitment

The whole concept was iteratively validated throughout the 3-stage iteration design process. Children were recruited through the project website [35], at diabetes summer camps, and via Facebook groups and through nonprofit organizations dealing with treatment support. The eligibility requirements were as follows: (1) fluency in Czech, (2) possession of a smartphone for a minimum of 2 weeks, (3) a confirmed diagnosis of T1DM for at least 1 year, and (4) aged between 5 and 16 years.

### Ethical Considerations

The design process was approved by the committee for research ethics at the Czech Technical University in Prague, Czech Republic (0000-01/24/51902/EKČVUT). Written study information was provided, and written informed consent was obtained from parents. All users agreed to provide anonymized data for research and data analysis during the sign-up process, which was required for app use.

### Development Approach

Agile principles [36] were used for the rapid development of various components and for iteratively integrating. Undergraduate and graduate students (computer science and biomedical engineering students) participated in developing new functionalities over 8 semesters. The game was prereleased on Google Play at the end of each iteration design process for feasibility testing. User feedback and bug fixes were communicated in weekly core team meetings and daily written discussions (via email and Slack). During the academic term, student meetings were also held once a week. A cross-platform solution, Unity software (Unity Technologies) [37], was used for game development; a free and open-source platform, Blender, was used for 3D modeling [38]; the GitHub tool [39] was used for versioning; and the Trello project management tool (Atlassian) [40] was used to track bugs and for project management. Furthermore, each task was developed in a separate branch on GitHub before being tested and approved by another team member and then merged into the master branch.

## Results

Herein are presented the results of the design and development phase that gave rise to the MyDiabetic game as a person-centered education tool, along with details of the contributions provided by each iteration of the participatory co-design methodology.

### Iteration 1: The Main Game Concept

#### Overview

In the first phase, the main game framework was outlined, focusing on carbohydrate counting and elementary technological tools for the measurement of glycemia levels and insulin administration. In addition, the basic gamification concept was proposed. The main aim of the usability study was the determination of user experience.

#### Individual Interviews and Design Survey

The co-design methodology was applied throughout the whole game design process. The main gaming concept was built in the first co-design iteration. First, qualitative (individual interviews) and quantitative research (design survey) methods were used to collect game design requirements. The additional documents for interviews are described in Multimedia Appendix 1 (refer to the Screener, Session Guide, and Design Survey subsections under Alpha Release). In addition, research was performed among parents, who are very important stakeholders in T1DM management. The questionnaire for parents is presented in the Questionnaire for Parents subsection under Alpha Release in Multimedia Appendix 1; a summary is presented in Table S1 in Multimedia Appendix 2.

User needs were specified, for which an informal interview was conducted. Four users with T1DM were invited to the interviews. Two observers were present during the interviews. One observer moderated the session, while another took notes, kept track of the time, and made observations. This was followed by rapid prototyping using the user-centered design methodology. The prototype used the basic paradigm mentioned in the Theoretical Background and Game Design section in Multimedia Appendix 1. The summary of the interviews is presented in Table S2 in Multimedia Appendix 2.

Parents play a big role in diabetes compensation. In this study, in children diagnosed at a young age, parents took care of insulin administration, the measurement of blood glucose levels, meal preparation, and writing in the diabetes diary. Parents would check their children regularly, some excessively, especially if they were physicians themselves. All participants used paper diabetes diaries because it was almost always the participants’ mothers who took up the responsibility of maintaining the diaries, and they did not seem to be very confident about using mobile app technology (if the children were to enroll, they would have preferred to use a mobile app). The children learned about managing the disease by observing their parents’ actions. Initially, when they were at school, they had to call their parents, who told them the appropriate insulin dose to inject. In time, the children became confident, and consulting their parents was no longer necessary. Quantitative research was carried out at diabetes summer camps (refer to the Design Survey subsection under Alpha Release in Multimedia Appendix 1 as well as Table S3 in Multimedia Appendix 2). In total, 27 questionnaires were collected among children aged 7 to 13 years. The main interest centered around where children obtained most of their information about diabetes and if they had ever encountered a game about diabetes. Other topics of interest were current favorite games among the children and how independent the children were in terms of counting bread units (BUs) and insulin administration.

On the basis of the results (refer to Tables S1-S3 in Multimedia Appendix 2), the main requirements for the game can be summarized as follows:

- Teaching the relationship among food, insulin, and physical activity to children with diabetes
- Teaching children with diabetes how to count bread exchange units
- Demonstrating the symptoms of hypoglycemia and hyperglycemia and their solutions
- Demonstrating insulin administration, including technical skills and dose adjustment, and explaining how to assess diabetes compensation by blood testing
- Explaining the nature of the disease
- Motivating children to exercise
- Being available free of charge and to as many people as possible
- Being both educational and fun so that children can continue playing it for as long as possible

When designing the game, the choice was driven by the experience of children from quantitative research (refer to Table S3 in Multimedia Appendix 2): the most played game was Pou, which involves caring for a simulated creature, which is a proven gameplay principle of other successful games such as Moy, My Talking Tom, My Talking Angela, The Sims, and the older Tamagotchi. These games are based on the player taking care of a pet. Games of this type have had great success in the past and in recent times [34,41].

The primary design concept of the game divides the avatar’s day into 6 parts, representing 6 meals, similar to the routine of a person with diabetes. The player’s tasks include measuring the avatar’s blood glucose level, administering insulin, and feeding the avatar. For completing these tasks, the player is rewarded with virtual coins, which can be used to enhance the avatar as well as buy new furniture, food, and other items. Neglecting the avatar’s care can result in the avatar experiencing hypoglycemia or hyperglycemia, which the player can identify based on the virtual glucometer reading and the avatar’s symptoms.

#### Onboarding and Tutorial

**Figure 2:**
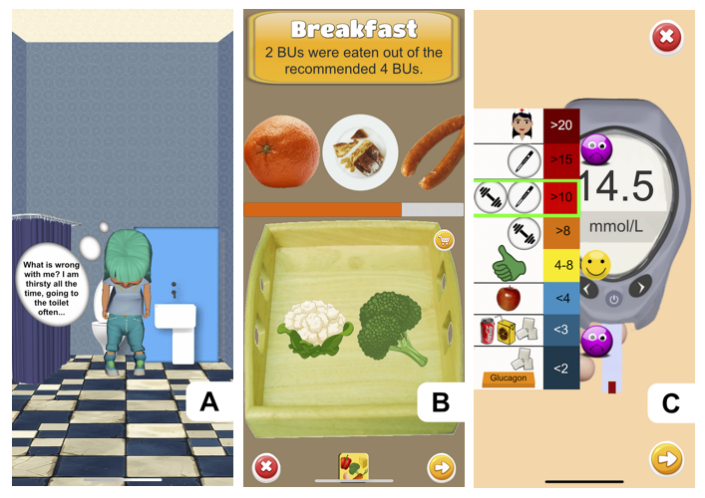
(A) Onboarding, including the first symptoms and diagnosis. (B) Carbohydrate counting. (C) Measuring blood glucose level using a glucometer. BU: bread unit.

The game includes an introduction in which the avatar experiences the initial symptoms of diabetes and is transferred to the hospital in an ambulance, where it is diagnosed by a physician (Figure 2A).

#### Control Elements

**Figure 3:**
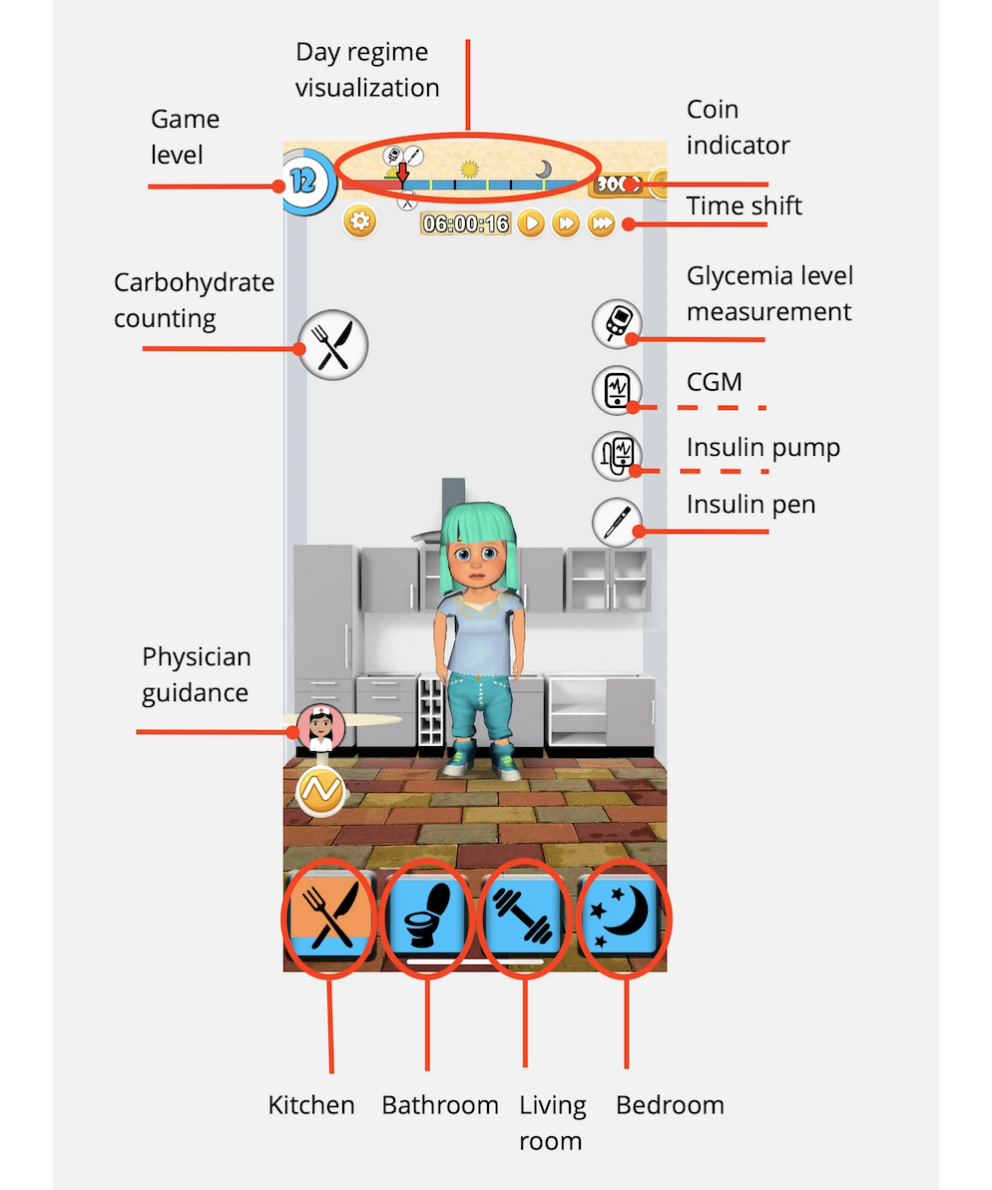
Description of the control elements. Continuous glucose monitoring (CGM) and insulin pen features were added in the final iteration (iteration 3) and appear in level 5.

Herein, the control elements of the game are described using the example of a kitchen scene (Figure 3). The different rooms are represented by an icon in the bottom panel. Each icon serves not only as a switch to access that room but also to indicate whether one of the avatar’s basic needs, such as sleep or food, is met. In the upper left corner, the player is reminded of the level the avatar is currently in. The key is to follow the daily routine, which can be seen on the timeline. The timeline is the same every day. During the repetition of basic actions, the player learns about the problem.

If needed, time can be accelerated using the designated button. The amount of coins currently available in the avatar’s cashbox is displayed on the right side.

**Figure 4:**
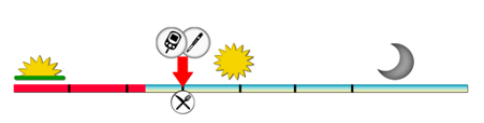
Illustration of the timeline for the day regime of a person with diabetes.

To remind players of basic tasks such as measuring blood glucose levels, injecting insulin, and eating, a timeline was designed that always shows 1 task ahead, and all tasks are marked on the timeline so that the player knows approximately when the next task will occur (Figure 4).

#### Carbohydrate Counting

The player must choose the proper combination from the offered food items to achieve the specified number of BUs as closely as possible. The offered food items will be selected randomly from the purchased food so that the player cannot cheat (eg, by learning the BU component of only 1 food item and repeatedly giving it to the avatar). The player can see food items as a picture of food on a plate to estimate the amount of food based on the plate size (Figure 2B).

#### Glycemia-Level Measurement

**Figure 5:**
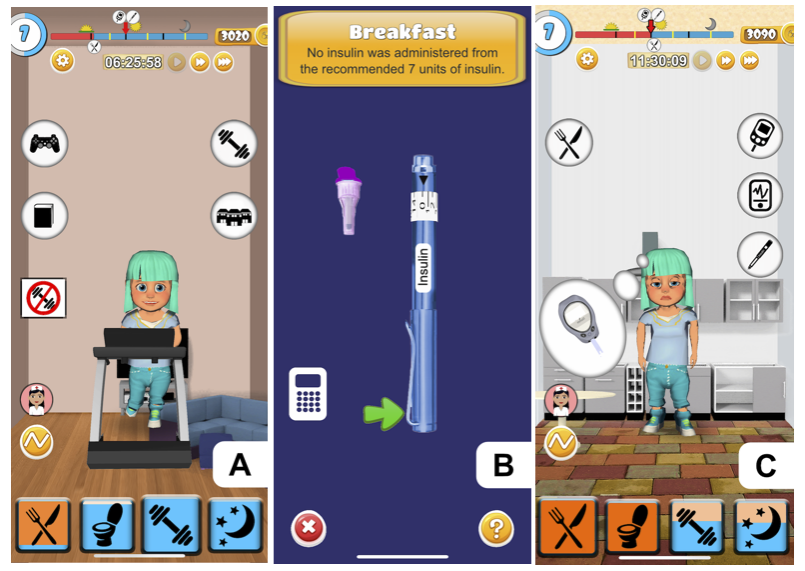
(A) Performing exercise. (B) Administration of insulin with an insulin pen. (C) Visualization of the symptoms of acute hyperglycemia.

There is no blood glucose–level indicator in the game. The player determines the exact blood glucose level by measuring it, for which they receive a reward. The virtual measurement mimics the real measurement (Figure 2C). The needle of the lancet becomes dull over time and needs to be replaced. A limited number of test strips are inserted into the pen and run out over time. The player must buy new needles and test strips in the virtual store with the collected coins. Estimating the blood glucose level based on the avatar’s current mood is possible; for example, in the case of hypoglycemia or hyperglycemia, the avatar appears sick and in a bad mood (Figure 5C).

#### Administration of Insulin

Insulin administration too mimics real-world settings. The player learns certain habits, such as pointing the needle skyward, giving the pen a little flick or tap to loosen any bubbles to the top, and performing an *airshot* (expelling air, thus priming the needle for injecting and avoiding possible air bubbles in the cartridge). The user is first required to assemble an insulin pen by removing the cap from the pen, taking a new needle, removing it from the sterile packaging, inserting it in the pen, and removing the needle cap and needle shield (Figure 5B). The game only allows the user to inject insulin once the needle has been primed for injecting. The game advises the user on the appropriate dose of insulin, as well as the type of insulin to administer to the avatar. The user administers short-acting insulin before main meals and long-acting insulin at night. Changing the needle and changing the insulin cartridge (the amount of insulin in it gradually decreases) are also incorporated.

#### Performing Exercise

The avatar requires regular exercise; otherwise, the avatar will be in a bad mood. The player determines the duration of the exercise (Figure 5A). If the exercise is too long, the avatar goes into hypoglycemia. The muscles tend to be more insulin sensitive for 1 to 2 days after exercise, leading to an increased risk of hypoglycemia, but this is not implemented in the game. To motivate the player to be active, the game offers them bonus coins for performing physical activity. If the player owns a Fitbit fitness bracelet, it is possible to connect it to the game and receive a virtual coin for every 2 steps taken in real life.

#### Alpha Usability Testing

The main aim of alpha usability testing was to determine users’ experience and assess their understanding of the main game concept. In total, 12 children participated in alpha testing (refer to Table S4 in Multimedia Appendix 2): 7 (58%) with diabetes (n=5, 71% boys and n=2, 29% girls; aged between 6 and 13 years) and 5 (42%) without diabetes (n=2, 40% boys and n=3, 60% girls; aged between 6 and 13 years). The involvement of children without diabetes is important to address the reduction of stigma and to provide education to friends and schoolmates. The recruitment was performed through the Association of Parents and Friends of Diabetic Children. The testing was organized at participants’ premises and consisted of completing the pretesting screener (refer to the Screener subsection under Alpha Release in Multimedia Appendix 1) and testing (onboarding and going through all 4 basic screens). One observer was always present to take notes.

Regarding user experience and game mechanics, all participants were satisfied; they liked the design and were able to find their way around in all scenes, although only after several attempts. Most users asked for help, both written and audio. Some users were looking for basic educational information about diabetes. The majority of users mentioned the issue of long-term motivation to use the game regularly. The main feedback is summarized in the Feedback Summary subsection under Alpha Release in Multimedia Appendix 1. Most shortcomings were addressed in future development in iterations 2 or 3.

### Iteration 2: Educational and Gamification Features

#### Overview

On the basis of the outcomes of the usability alpha testing and continual feedback from the clinical team (nurses and physicians), in the next iteration phase, the focus of the main design effort was on increasing adherence, game experience, and educational impact by incorporating (1) game level design (Figure S2B in Multimedia Appendix 1); (2) minigames concept and storyline (Figures S3A, S8A, and S8B in Multimedia Appendix 1); (3) virtual guidance; and (4) an educational library, including the simulation of glycemia [42] (refer to the Theoretical Background and Game Design section in Multimedia Appendix 1 for a description of the new add-ons). Incorporating the audio guidance helps preschool children who cannot usually read yet to better orient themselves within the game. The main aim of the feasibility follow-up testing was the determination of educational and adherence effects.

#### Beta Usability Testing

The recruitment was organized via Facebook groups and targeted children aged 5 to 15 years willing to play the game for at least 1 week. Children without diabetes were not included because this feasibility test aimed to determine the educational and adherence effects in children with T1DM. In addition, adherence was tested by inspecting game statistics implemented by applying the Google Analytics framework.

##### Individual Interviews

The interviews were conducted in person with the participant and their parents. The first questionnaire was the screener (refer to the Screener subsection under Beta Release in Multimedia Appendix 1), which helped to place the children in the correct category corresponding to the desired target group of the participants. This was followed by a general questionnaire (refer to the General Questionnaire subsection under Beta Release in Multimedia Appendix 1) to estimate the participant’s interest in games. Furthermore, the participant was asked questions regarding their management of their diabetes (refer to the Diabetes Management subsection under Beta Release in Multimedia Appendix 1). The user was also presented with a questionnaire on diabetes education (refer to the Pretesting Knowledge of Diabetes subsection under Beta Release in Multimedia Appendix 1). Subsequently, the game was installed on their mobile device. This was followed by observation of the participant and their behavior as they undertook the first steps in the game. The participant was required to go through the whole game tutorial and later try to find their way around the game for a few minutes. The gameplay was then interrupted, and the participant was asked about first impressions (refer to the First Impression of the Game subsection under Beta Release in Multimedia Appendix 1). The participant’s parents were then interviewed (refer to the Questionnaire With Parents subsection under Beta Release in Multimedia Appendix 1). The main findings of the interviews are summarized in Table S5 in Multimedia Appendix 2. Seven participants took part in the study, of whom 1 (14%) did not complete the usability testing and was excluded. Of the remaining 6 participants, 1 (17%) was a boy and 5 (83%) were girls, and they were aged 5 to 15 years. The average duration of diabetes was 4.8 (SD 2.9) years; 4 (67%) of the 6 participants were using an insulin pump.

##### Beta Usability Testing

The participant was asked to play the game for at least 1 week, after which they were contacted by telephone, and the second part of the test questionnaire was discussed. First, the participant was asked about their impressions of the app after the 1-week testing (refer to the Beta usability testing subsection under Beta Release in Multimedia Appendix 1), followed by a knowledge questionnaire on diabetes (refer to the Posttesting Knowledge of Diabetes subsection under Beta Release in Multimedia Appendix 1). The educational effect of the game is outlined in the next subsection. Regarding the usability testing results, the main findings are summarized in Table S6 in Multimedia Appendix 2.

In summary, the participant group consisted of children in different age ranges. Of the 7 participants, 2 (29%) were aged <7 years and could not read; 1 participant (14%) could not complete the usability testing because he required hospitalization. Of the 4 participants aged 8 to 12 years, 3 (75%) expressed high levels of enjoyment and found the game entertaining. Evidently, all children in this category understood the game well, confirming the study hypothesis that this age group should be the primary focus. Of the 6 participants who completed the testing, the remaining 2 (33%), aged 13 to 15 years, also understood the game well, although they commented that the entertainment aspect could have been more engaging. They highly recommended the game to younger audiences and stated that they wished such an educational tool had been available when they had been first diagnosed with diabetes. In general, the girls particularly enjoyed shopping, while the boy was more interested in minigames.

##### Educational Effect

Table 2 summarizes the educational impact based on the answers to the questionnaires, showing the level of education before and after the testing. The specific percentage response rates are given in Tables S7 and S8 in Multimedia Appendix 2. This score is expressed as a percentage depending on the quality of the correctly answered questions and the degree of confidence in answering them (assessed subjectively). If the answer is accurate and complete (correct), then 100% is given for this question. If the answer is partially correct or incorrect, a percentage measure of accuracy is estimated, where 0% is given for an outright nonsensical answer or if the participant does not know the answer.

**Table 2:** Effect of education.

	Educational score before testing (%)^a^	Educational score after testing (%)^b^	Difference (%)^c^
Participant 1	39	44	5
Participant 2	96	98	2
Participant 3	27	55	28
Participant 5	80	93	13
Participant 6	84	95	11
Participant 7	89	94	5

^a^Average 69.2.

^b^Average 79.8.

^c^Average 10.7.

Table 2 depicts the results of the educational effect after 1 week of usability testing. On average, the increase in education knowledge was approximately 10%; in the particular case of participant 3, the gain was considerably high: 28%.

##### Statistics Summary

Regarding game analytics, a significant improvement in skills was observed throughout the gameplay, specifically in the time taken to perform certain procedures. The time taken for glycemia-level measurement decreased by 42%, and the time taken for insulin administration decreased by 45% from the first interaction (day 1) to the last interaction (day 7).

Analyzing the purchase behavior of players, the most frequently bought food items were the large basket (46%), followed by the medium-sized basket (26%), and the small basket (28%). As for medical supplies, the majority of purchases consisted of glucometer strips (59%), followed by lancet packs (18%), injectable lancets (12%), and insulin (11%).

Examining the distribution of glycemia statistics, glucose measurements were most commonly applied on the middle finger (50%), followed by the index finger (22%), ring finger (19%), little finger (6%), and thumb (3%). Short-term insulin injections were most frequently applied to the abdomen (29%), left biceps (12%), and left thigh (7%). Long-lasting insulin injections were predominantly applied to the left and right thighs (11% and 12%, respectively) and to the chest (6%). The less frequent injection site was the right thigh 0.78%, of the 129 injections recorded.

During the gameplay, the avatar was hospitalized 31 times, with 15 (48%) of the cases involving hypoglycemia and 16 (52%) involving hyperglycemia. The average blood glucose level during these hospitalizations was 15.7 mmol/L. In addition, the avatar engaged in exercise 72 times, with 36 (50%) of the sessions lasting for 30 minutes, 19 (26%) for 90 minutes, and 17 (24%) for 60 minutes. However, the exercise was completed in only 32 (45%) of the cases, with early terminations occurring in the remaining 40 instances (55%).

**Figure 6:**
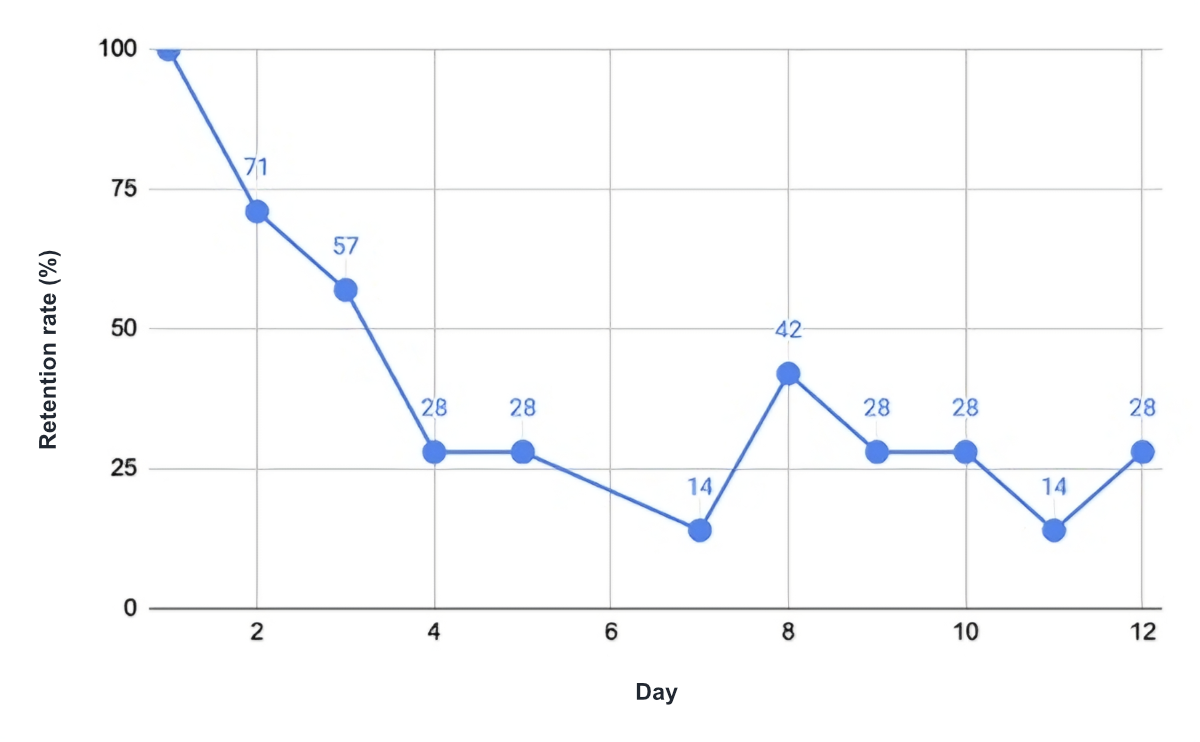
Retention rate of participants.

The final phase of the usability testing focused on measuring adherence. At the beginning of the game, the day and time were recorded to mark the initial interaction. Subsequently, for each following day, whether the player returned to the game was noted. The retention rate, depicting the percentage of players who continued playing the game over time, is illustrated in Figure 6.

In the first week, the participants tested the game without any intervention. The telephone interview was performed on day 7. As shown in Figure 6, the adherence rate was >14%, and on the following day, day 8, the rate increased to 42% after the interview. The time spent playing the game on day 1 was approximately 39 minutes per user; on day 7, it was approximately 25 minutes. Some participants even played the game for 1.5 hours on 1 day. The daily average time for all participants was approximately 17 minutes.

##### Participant Feedback

Given the high number of participants (70%) using insulin pumps, it was unsurprising that there were requests to add insulin pump functionality and CGM in the game. Some users also expressed a desire for more advanced tools, such as the ability to administer glucagon and measure ketoacidosis. In response to this feedback, these features were included in iteration 3 of the game.

Of the 6 participants, 2 (33%) indicated that the game became routine after a certain period and that they desired some variation. Of these 2 participants, 1 (50%) suggested that a solution could be to introduce complications related to diabetes, such as cardiovascular issues, neuropathy, or retinopathy, which the player would need to manage.

In the real world, various types of insulin are available, each designed with different properties regarding onset, peak, and duration. Participants expressed the desire to be able to purchase and use the specific insulin types that they are familiar with and to understand their characteristics within the game.

### Iteration 3: Advanced Technological Components

On the basis of the feedback received from the beta usability testing, in this iteration, more advanced tools for diabetes management were implemented. Subsequently, the final usability study briefly evaluated the new modules.

#### CGM and Insulin Pump Implementation

This module illustrates the use of the application of the CGM sensor (Figure S4 in Multimedia Appendix 1) and tethered insulin pump (Figures S5 and S6 in Multimedia Appendix 1). When the player’s blood glucose levels reach a certain threshold (>5mmol/L), the advanced technology of using a CGM sensor and an insulin pump becomes available, unlocking new features and options in the game (see insulin pen and insulin pump icons in Figure 4).

#### Glucagon Administration

Glucagon administration is an essential treatment for severe hypoglycemia, particularly when a person with diabetes cannot consume food or fluids because they are in a hypoglycemic coma [12]. Another individual must administer the glucagon injection in such cases. The selected design closely resembles the real-life GlucaGen HypoKit (Novo Nordisk A/S) and visually represents the glucagon injection process (Figure S7B in Multimedia Appendix 1). The glucagon administration icon will be displayed when the avatar experiences hypoglycemia, indicating the appropriate action to take.

#### Measuring Ketoacidosis

Specifically, patients treated with continuous subcutaneous insulin infusion have a greater risk of developing diabetic ketoacidosis because there is no subcutaneous depot of insulin, and therefore ketoacidosis can occur much faster [1]. As the measurement of ketone bodies from urine using diagnostic strips is the most common and most accessible method in the home environment, the focus is on designing the scene using the home kit (Figure S7C in Multimedia Appendix 1). To simulate the level of ketoacidosis, the simplify model from the work by Fabietti et al [43] was applied (refer to the Ketoacidosis Module Implementation section in Multimedia Appendix 1).

#### Gold Usability Testing

The primary objective of the usability testing was to evaluate the technical feasibility of the advanced modules. Participants were recruited from a summer camp for children with diabetes. In the first phase, a general questionnaire was administered to assess the participants’ knowledge about diabetes compensation. A total of 30 participants (n=12, 40% boys and n=18, 60% girls) aged between 7 and 15 years completed the questionnaire (refer to Table S9 in Multimedia Appendix 2). In the second phase, the 24 participants completed the tutorial (refer to the Onboarding and Tutorial subsection) together. Finally, the participants were divided into 3 groups to test the insulin pump and CGM sensor module (9/24, 37%), the ketoacidosis measurement module (7/24, 30%), and the glucagon administration module (8/24, 33%). Before testing, a pretesting questionnaire (refer to the Pretesting Questionnaire subsection under Gold Release in Multimedia Appendix 1) was administered. The usability testing session was conducted by a researcher, who explained the main goals to be achieved in each group in several steps.

A cohort of 9 participants (n=2, 22% boys and n=7, 78% girls) were enrolled to test the insulin pump and CGM sensor module. The study required the participants to complete several tasks, including applying the glucose sensor and insulin pump infusion set, calibrating the sensor, and spending a portion of the day using the sensor and the pump. The summary of the testing is provided in Table S10 in Multimedia Appendix 2. Overall, the insulin pump and CGM sensor module were rated positively for their realism; however, participants reported that certain complications were not accounted for in the game, such as disconnecting the infusion set from the reservoir, the possibility of the pump tubing becoming kinked and clogged, and adding insulin to the reservoir. During the pump assembly (refer to step 2 in Table S11 in Multimedia Appendix 2), participants had difficulty connecting the reservoir to the infusion set using tubing and squeezing the air out of the tubing by long pressing the button on the tank. In addition, confusion arose during the insulin infusion scene because participants needed clarification on how many units of insulin they should inject. Improvements in this scene could help better convey the necessary information to the player.

Eight participants were involved (n=4, 50% boys and n=4, 50% girls) in the testing of the glucagon administration module (refer to Table S12 in Multimedia Appendix 2). All participants were able to administer glucagon; however, sometimes the game took longer than expected due to minor bugs or nonintuitiveness, such as using a click gesture instead of a drag-and-drop gesture, which caused problems in cases of drawing the solution back into the syringe and then removing the syringe from the vial or removing the protective cover of the syringe. All participants praised the realistic handling of the game.

The ketoacidosis measurement module was successfully tested by all 7 participants (n=2, 28% boys and n=5, 72% girls), who found the scene good and did not suggest any improvements (refer to Table S13 in Multimedia Appendix 2). Only 1 (14%) of the 7 testers did not observe an increase in ketone bodies when insulin was not administered to the avatar. When asked about alternative methods of measuring ketones, most participants expressed openness to new options beyond diagnostic strips, especially for children accustomed to different measurement methods.

## Discussion

### Principal Findings

The main objective of this paper was to provide education mainly to children newly diagnosed with T1DM by using mobile technologies and gamification theory for educational purposes. The inclusion of gamification in diabetes management offers additional benefits compared to current methods, particularly in terms of improving patient motivation [4,5]. Given the widespread use of mobile technologies among young children, a mobile app is an ideal platform for introducing this serious topic to them entertainingly and engagingly. This approach involves combining various techniques, such as gamification theory [24] as well as topics from behavioral theory, such as tailoring [11], observational learning [29], decision-making practice [13], social and family support [30,31], and reward systems [32], to support patient motivation and compliance.

The game provides comprehensive education for children on various aspects of diabetes, including technical skills such as measuring blood glucose levels and administering insulin, as well as explanations of the nature of the disease. As a result, the game should be considered an additional resource for children newly diagnosed with T1DM to learn about the disease, along with books and brochures. The game’s virtual avatar can also serve as a supportive friend for children unable to attend diabetes camps.

In addition, the game has the potential to raise awareness of diabetes among children without diabetes as well as parents [1]. By increasing knowledge of the disease and its symptoms, the game can aid in earlier diagnosis and treatment of diabetes. Initial feedback from children with T1DM and their parents on the game has been positive, with many expressing enjoyment and a willingness to participate in further testing.

Concerning the educational effect of the game, after a week of testing, there was a noticeable improvement in the educational outcomes (refer to Table 2). When compared to a previous study [16], it can be inferred that the improvement rate in educational outcomes is similar (33%). However, it is important to consider that the game in that study was tested for a year, while in this study, testing only lasted a week. In comparison, another study [18] reported an improvement rate in educational outcomes of 7% only. MyDiabetic aims to enhance traditional patient education formats (such as patient information booklets [44]) rather than replace them.

Recent literature reviews on gameful eHealth and mobile health tools have indicated that the most commonly used game elements are externally oriented, such as points and rewards [45,46]. The concept of avoiding excessive use of externally oriented motivational features has also been explored in theoretical works on designing engaging and gameful experiences [47]. Nevertheless, in a review of similar apps targeting gaming in T1DM (refer to Table S14 in Multimedia Appendix 2), authors found that the archetypal game elements of points, badges, and competitions were the primary approaches used [48]. MyDiabetic involves using rewards to purchase food, goods, and healthy supplements, which appeal to most children. The shopping aspect serves as an educational tool that prepares young children to increase self-efficacy in managing their T1DM, consequently reducing their dependence on their parents [5,17].

One of the most effective aspects of the co-design process in achieving design goals was the participation of clinicians, patients, family caregivers, developers, and game designers. Participants brought their unique expertise to the table and contributed to the design process; for example, health care researchers provided knowledge and feedback on diabetes compensation, while the developers and designers worked on making the tool user-friendly, and the external game designer suggested more interactive and immersive solutions. Research indicates that collaborative, team-based approaches are recommended for developing mobile health interventions [49]. The strategic coordination of stakeholder involvement at each stage of development was a key benefit of this design approach.

Although there is still no consensus on the optimal combination of game mechanics for serious games [34], it is acknowledged that MyDiabetic currently leans too heavily toward education, leading to lower retention rates than anticipated. Drawing from the insights gained during the second and third rounds of the iterative usability studies, it is proposed to redesign the game by incorporating multiple storylines, such as school experiences, dating, and career development, to enhance the entertainment factor and introduce new topics as the avatar ages. The existing educational components will be seamlessly integrated into the game mechanics to improve overall gameplay and increase engagement. This new direction should also provide an easier transfer of knowledge and skills from this serious game to real-world situations [34].

### Limitations

Initially, all participants involved in the study voluntarily opted to participate or were contacted by the project team or collaborating institutions. Consequently, the participating groups may have had higher motivation, resourcefulness, and better chronic illness management, thus creating a potential bias that may only represent a portion of the user group. Nonetheless, this is a common limitation in this type of research.

While the sample sizes were indeed modest in the alpha, beta, and gold testing phases (12, 6, and 24 participants, respectively), the significance of the results lies in the qualitative insights and detailed observations that were gathered through iterative usability testing. This process enabled deep delving into the experiences and perceptions of each participant, yielding rich and meaningful data on the game’s usability and educational impact. Furthermore, the results that were obtained from the limited numbers of participants were consistent and provided actionable insights for further refinement and development of this serious game.

The design outcomes were influenced by the stakeholders involved in the project, who had the potential to both positively impact and limit the final design. One example is the nutrition nurse who facilitated the design activities but had limited design experience and had to learn co-design processes as the project progressed.

The MyDiabetic app was developed and evaluated solely by Czech users, although an English version is now accessible. There may still be cultural discrepancies that have not been fully addressed. The project team constantly receives feedback and makes necessary adjustments to improve the app’s usability and relevance.

It is important to note that this paper focuses solely on the technical development of the game and user acceptance testing. The clinical validation of the game, using metabolic control markers such as mean glycated hemoglobin levels, is beyond the scope of this paper. While it is important to evaluate the effectiveness of the game in improving diabetes management, this will be the subject of future research.

### Comparison With Prior Work

After examining the survey presented in Table S14 in Multimedia Appendix 2 [13,16,18,19,21,24-31,33,42,43,50-65], it was observed that all games, except Packy & Marlon [16], lacked clinical validation. Furthermore, assessing adherence was not a focus in any of the games. In addition, of the 18 games, 3 (17%) were previously available on Nintendo or mobile game stores, but currently only 2 (11%; MyDiabetic and Jerry the Bear) are available for download on mobile game stores. Most games were developed as prototypes or concept studies as part of academic projects, with only a few aimed at preschool children [50-52]. Only 1 (6%) of the 18 games addresses both basic (carbohydrate counting, blood glucose–level measurement, and insulin administration) and advanced (CGM and insulin pump) educational self-management skills [50]. Nevertheless, no game tackles the complexity of diabetes management from such a wide perspective as MyDiabetic, in which unique features such as glucagon administration and ketoacidosis modules are included.

One of the challenges that serious game designers face is the *uncanny principle*, also known as the *uncanny valley*. This phenomenon occurs when a representation of a human or an animal looks and behaves almost but not exactly like the real thing [66]. The result is a feeling of eeriness, discomfort, or even revulsion in the observer. The *uncanny principle* can affect the learner’s immersion, engagement, and emotional connection with the serious game. The *uncanny valley* can be useful for creating more effective and engaging serious games. By carefully navigating the *valley* and finding the right balance between realism and abstraction, game designers can create experiences that are both educational and emotionally compelling [67]. MyDiabetic aimed to strike this balance by using cartoonish avatar designs and environments, while carefully incorporating technology features that closely resemble real-world settings, setting it apart from most other games [16,50].

### Future Directions

To enhance the game’s realism, it would be beneficial to incorporate real-time elements. This would involve the avatar aging as time passes In addition to managing diabetes symptoms, the avatar would also engage in typical daily activities such as attending school/work, participating in outdoor sports, visiting friends, and partaking in entertainment (Figures S8A and S8B in Multimedia Appendix 1). In addition, chronic retinopathy or diabetes foot problems would also be demonstrated (Figure S8C in Multimedia Appendix 1). By including these elements, the game could provide a more comprehensive experience for users and better prepare them for managing their diabetes in real-life situations.

Including social support in the app could be an additional valuable feature [68]. Games that allow users to connect with others in a community-type setting or through social networks could be particularly beneficial because research suggests that increased social support is linked to improved self-efficacy practices and better clinical outcomes in children with diabetes. A review of studies also found that social media were commonly used to facilitate self-care in patients and caregivers, with 77.1% of the identified studies reporting such use [69].

### Conclusions

This paper has shown how participants involved in co-design activities played a creative and productive role in shaping the content and design of the MyDiabetic app.

The main objective of creating a serious educational game was to captivate children with T1DM and also make it accessible to those who are interested in learning about the disease. On the basis of the testing described earlier, the educational goal was achieved because significant enhancements in children’s understanding were evident after a mere week of gameplay. The game was well-received by the participants, who expressed a willingness to recommend it to their friends or siblings who are not affected by diabetes but are curious about the disease and would like to understand it better.

To summarize, this research suggests that the target audience for this game is children aged between 5 and 12 years, with those in the 8- to 12-year range being able to fully engage with and benefit from all the features the game offers.

## Appendix

Multimedia Appendix 1 Additional theory background, questionnaires, and details of implementation.

Multimedia Appendix 2 Results of the feasibility testing.

Multimedia Appendix 3 PowerPoint (Microsoft Corp) presentation showing key aspects of MyDiabetic and related material.

Multimedia Appendix 4 MyDiabetic video.

## Abbreviations

BU: bread unit
CGM: continuous glucose monitoring
T1DM: type 1 diabetes mellitus
