# Supplementary material for: A Serious Game (MyDiabetic) to Support Children’s Education in Type 1 Diabetes Mellitus: Iterative Participatory Co-Design and Feasibility Study
Source: JMIR Serious Games. 2024 May 7;12:e49478. doi: 10.2196/49478 (PMC11109855; doi:10.2196/49478)
Supplement: Multimedia Appendix 1 [file games_v12i1e49478_app1.pdf]

Appendix 1. Supportive material

A0. Theoretical Background and Game Design

A0.1 User flow

There are several ways to motivate the player. One of the often-mentioned principles is to focus on user flow [1]. It is based on balancing between the game challenge and the user's skill. The game should strike an optimal balance between these factors because in the case of dis-balance, the game can make the player feel tension (the game is too difficult for him) or the game is uninteresting (the player is bored). In both cases, there is a high probability that the player will soon stop the game.
The game-flow characteristic, which contributes to the equilibrium between the skills of the player and the difficulty of the game has the following conditions:

- Specific and clear objectives with distinct rules. The objective is to balance compensation for diabetes treatment, reflected in the main design inspired by the Tamagotchi principle.
- The requirements of the objective correspond to the abilities of the player. The definition of the target group is a crucial factor. We hypothesised that the optimal age group for the game is between 6 to 12 years old.
- Clear and timely feedback concerning performance and precision. Instant feedback is incorporated in the main screen in case of taking food, including or measuring glycemia, virtual MD will also alarm the player.
- Reduction of non-influential external distractions, which increases concentration. We included in the game design non-educational topics such as minigames or storyline development in the later development stage.

A0.2 MDA framework

The game's design draws inspiration from a formal framework known as the MDA framework, which stands for Mechanics, Dynamics, and Aesthetics [2]. This framework serves as a bridge that unites game design and development, emphasizing the holistic integration of these elements. Aesthetics, within this context, encompasses the range of desired emotional responses evoked in players as they engage with the game system. It's worth noting that designers and players often hold distinct perspectives on what constitutes a game's most pivotal aspect. This divergence

frequently influences whether a player develops a fondness for the game or not. The achievement of entertainment and eventual success in a game involves the consideration of several criteria, some of which include:

Narration: This pertains to the game's ability to provide compelling storytelling and evoke a sense of drama.
Challenge: The evaluation of whether tasks and obstacles retain their degree of difficulty after an extended period of gameplay.
Competition: The exploration of the potential to outperform fellow players and claim the title of the best.

Dynamics, on the other hand, encompasses the behaviour that unfolds during runtime. It encompasses how the game's mechanics respond to player inputs and interact with each other's outputs over time which is very nature process in case of self-management of T1D. The ultimate goal is for players to comprehend the game's conclusion conditions and know when they've emerged victorious (see section Iteration 2: Educational and gamification features). By constructing models capable of describing and predicting these gameplay dynamics, designers can sidestep potential design challenges.

Mechanics, as the third facet, involves the fundamental constituents of the game, delving into aspects like data representation and algorithms. Even subtle adjustments to mechanics can lead to the emergence of a novel game, extending its potential to captivate players. A simple tweak in mechanics can introduce new dynamics, prompting players to adopt fresh strategies and view the game from distinct vantage points. In our game, this concept is manifested through a series of elaborated minigames.

A0.3 Behavioural theory

The game was also guided by applying behavioural theory, which is considered effective in motivating, encouraging learning, or changing the user's behaviour suffering from chronic conditions: the opportunity for tailoring, observational learning, decision-making practice, family support, and rewards systems.

Tailoring

The ability to tailor a game to the user’s needs or preferences is important in improving the game’s relevance and effectiveness [3]. Tailoring might allow the player to modify the avatar to look more like him or herself or an animal or avatar they like. Tailoring makes the user's experience more relevant and effective, as a user's attention is more likely to be attracted and maintained during gameplay [4]. Research has shown that tailoring can be an effective facilitator for producing behavioural changes, particularly with more complex behaviours such as changes in diet and exercise.

Observational learning

Using modelling and observational learning through game avatars that model behaviours can also be highly effective in increasing knowledge, teaching specific skills, demonstrating problem-solving or decision-making, and setting goals [4]. In particular, children and young people frequently learn behaviour or skills by observing others receiving feedback (whether positive or negative) as a result of their performance of that skill or behaviour [5,6]. In contrast to a more passive educational activity, such as watching a video, observational learning in game playing can be more active as players are likely to be more interested in the avatars, especially if they feel part of the avatar's world. Observing an avatar receiving a reward for making a smart choice, such as checking BG levels or administering the appropriate dose of insulin, may reduce apprehension and encourage children to engage in such behaviours themselves.

Decision-making

Games can also provide the ability for trial and error without fear of real-world failure, which can be a less intimidating means of practising skills. If errors occur, users can try again and learn from their mistakes [7]. In our design concept, a good example is when the player needs to take appropriate steps in case of hyper or hypoglycemia.

Family support

Diabetes education is the main therapeutic tool to achieve the implication of the family in the care of children with T1DM, and the best metabolic control is achieved when participation and intervention of patients and families are adequate. Family empowerment refers to caregivers who have received more comprehensive training in managing diabetes to make them able to make decisions in multiple situations [8]. This results in improved blood glucose control, and treatment optimisation is greater

as the modality is intensified [9]. In most cases, the family is a part of the game experience involving at least one family member when the child shares his observation from the gaming. Parents also played an active role in game design.

Reward system

Finally, as espoused in social cognitive theory, the use of external rewards, incentives, or reinforcements, whether a points system, a make-believe currency system or verbal praise, can be both an effective motivator for the player as well as a valuable learning tool [9,14].

A0.4 Game Design

Scene's description

The game MyDiabetic consists of a basic scene in an apartment. There are four rooms - kitchen, bathroom, living room and bedroom. There are also scenes for individual activities that the player can perform. Figure S1 shows a graph of all the scenes depicting basic transitions between them.

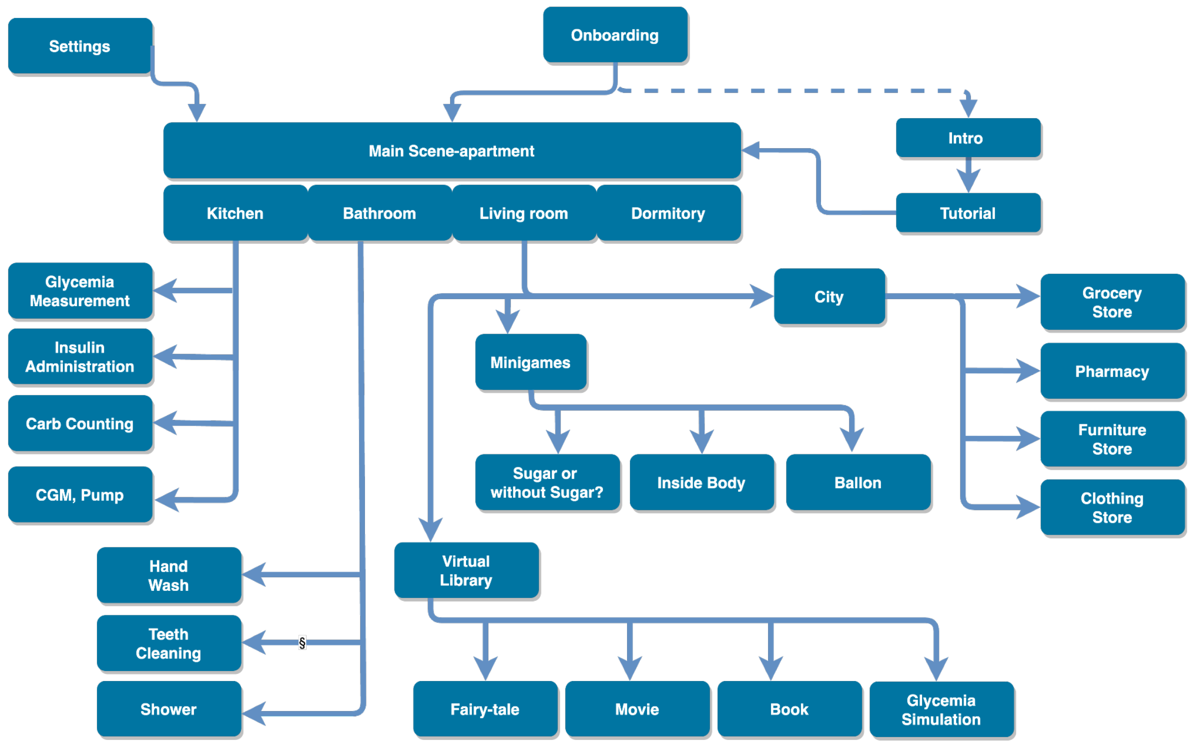

Figure S1. Scenes graphical representation

Game mechanics are implemented centrally using a shared game manager. The game manager cooperates with an extensive state machine. This automaton contains all possible states that can occur in the game. Transitions between states are dependent on actions performed and time elapsed. An example of the game manager is outlined in A6.Tutorial description.

Avatar tailoring

The game includes a virtual clothing store for the avatar, where the player can customise the avatar to their requirements. In another store, the player can buy new equipment for the apartments, so each room has different types of furniture. Other stores include a food store and a store with diabetic supplies, as depicted in Figure S2(a).

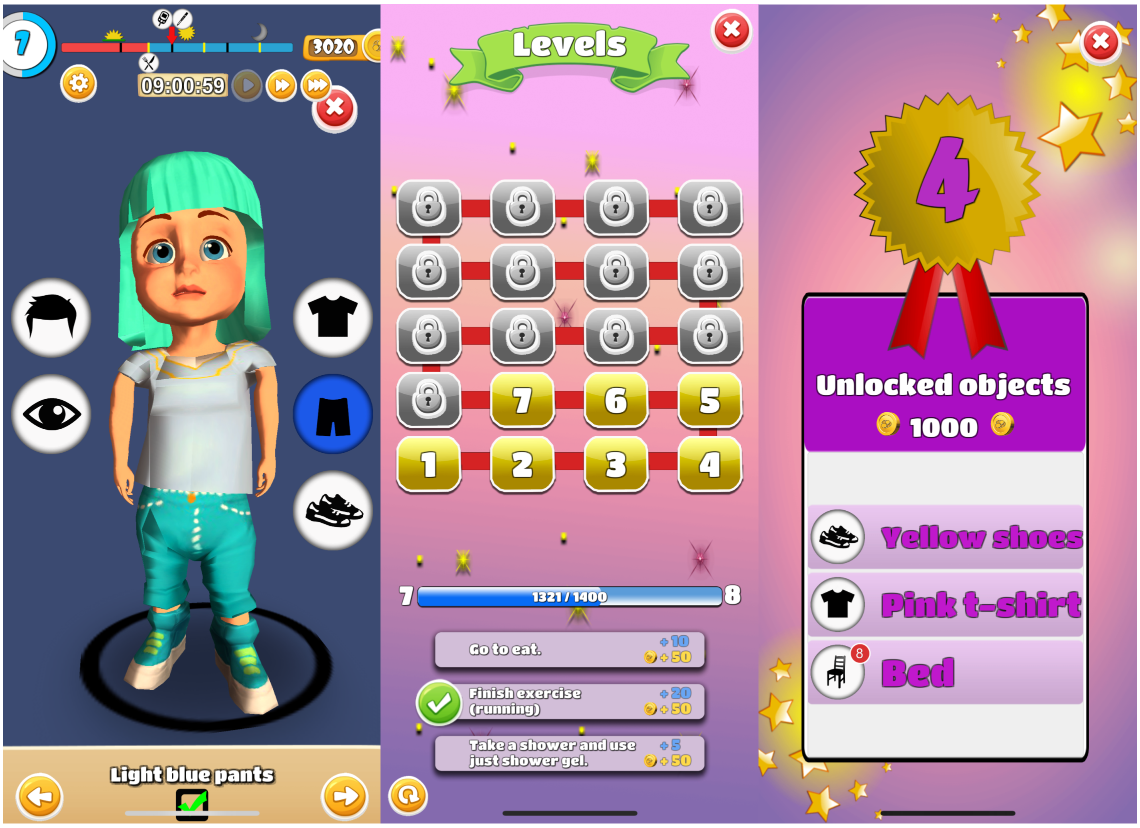

Figure S2. (a) tailoring, (b) levels and three tasks visualisation, (c) task description

Level progress design

The levels visualisation can be reached by clicking on the level indicator in the upper right screen - see Figure S2(b). In each level, the new items (e.g., clothing or furniture) are opened after the player accomplishes the specific tasks. The new open items need to be bought using virtual game currency.   Another important part of the game adherence is the presence of tasks. It is a list of activities that the player is expected to perform. For their successful completion, the player will receive a reward of coins and experience points. These experience

points are used to advance in levels. The tasks are presented in the level scene in the lower part - see Figure S2 (b),(c).

Minigames concept

In addition, the game includes minigames that serve as a supplementary learning tool for diabetes, reinforcing knowledge while adding an element of fun and relaxation to the overall game experience. These minigames focus on activities such as practising carbohydrate counting or selecting food items and placing them into two baskets based on their sugar content (refer to Figure S3(a)).

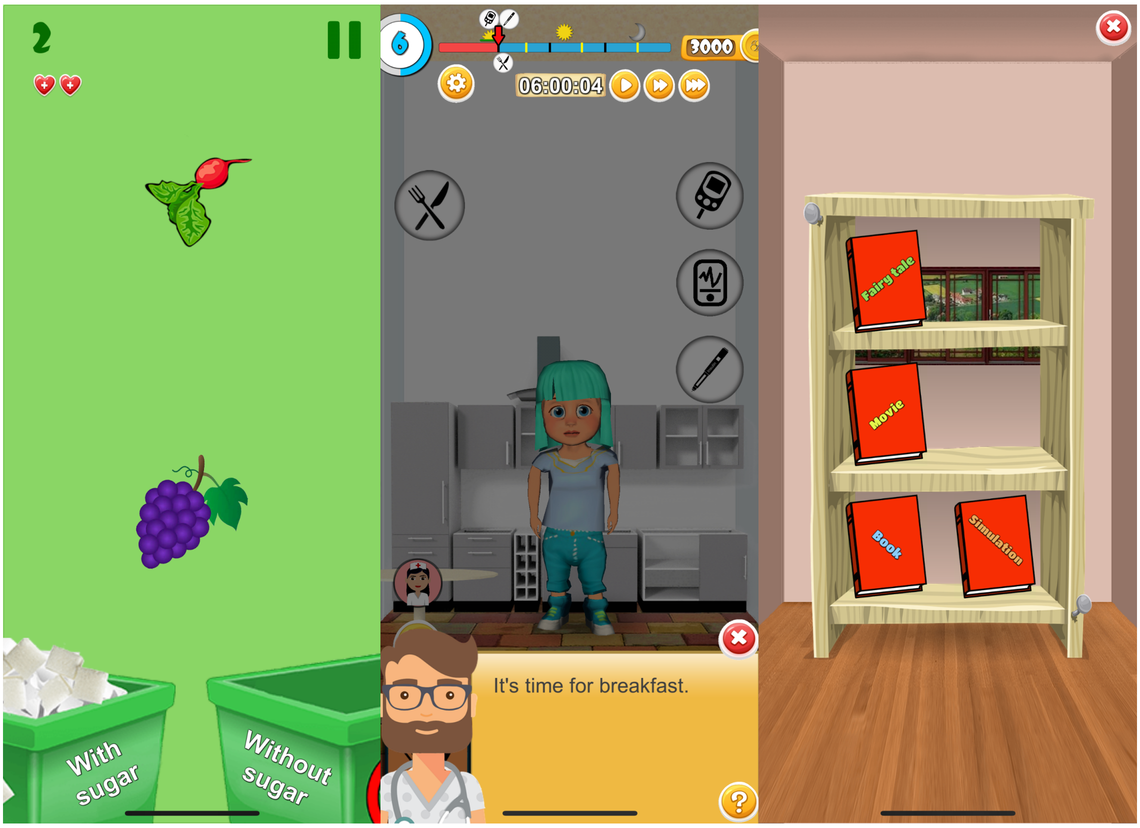

Figure S3 (a) Example of a minigame, (b) virtual physician, (c) virtual library

Virtual physician

During the iterative design process and through valuable feedback obtained during alpha testing, we recognised the need for guidance within the game, particularly when children encountered unfamiliar, unpleasant, or challenging situations that required problem-solving. To address this, we introduced a "virtual help" feature in the form of advice from a male or female medical physician (MD) displayed on the screen. The MD recommends the next steps or provides insight into what might be going wrong. To ensure minimal disruption to the player's experience, the MD avatar is positioned in the corner of the screen (see Figure S3(b)). Additionally, at the beginning of the game, the MD reminds players about the importance of

timely meals, glucose measurements, and insulin injections. The MD communicates verbally to cater to younger children who may not yet be able to read. To enhance the sense of realism, instead of utilising a text-to-speech engine, all the text was recorded with native English speakers providing voiceovers. The gender of the MD corresponds to the choice of the avatar's gender made at the beginning of the game; if the avatar is male, the MD will be female, and vice versa.

Educational interactive library

The game is aimed not only at children with T1DM but also at those involved with the disease as siblings, friends, schoolmates and acquaintances to educate the player's circle. Even children with long-term diabetes can still learn some advanced topics. Therefore we incorporated the concept of the educational library, which includes an interactive book about diabetes, a fairy tale explaining diabetes, a youtube video, and a simulator of blood glucose (see Figure S3c and Section Simulation of blood glucose).

Simulation of blood glucose

The glycaemia model is an important part of the game. The simulation model chosen for the game is AIDA (An Education Simulator for Insulin Dosage and Dietary Adjustment in Diabetes). Glycaemia is sampled every fifteen minutes. The remaining values are calculated by linear interpolation of these samples [10] In addition, we created a simple simulator that is part of the Educational library.

A1. Alpha release (Iteration 1)

A1.1 Screener

1. How old are you?
    (a) less than 7
    (b) 7 - 10
    (c) 11 - 15
    (d) more than 15

2. Are you a girl or a boy?
    (a) girl
    (b) boy

3. Do you have time for an interview (45 min.)?
    (a) yes

(b) no

4. Are you diabetic?
   (a) yes
   (b) no

5. If you're diabetic, what type?
   (a) type 1
   (b) type 2

6. How often do you play games?
   (a) every day
   (b) at least 3 times a week
   (c) at least once a month
   (d) at least once a year
   (e) not at all

7. If you play games, where?
   (a) on your mobile phone
   (b) on your computer
   (c) on a console (XBOX,Wii,...)

8. Do you have your own mobile phone or tablet?
   (a) yes
   (b) no

A1.2 Session Guide

A1.2.1 About gaming

1. gaming in general
   (a) When do you play games? At break time at school? At home? In the car?...
   (b) Where do you find out about new games?
   (c) How old have you been playing since?
   (d) What do you play games on?
   (e) What do your parents say you play?
2. Favourite games
   (a) What is your favourite game?
   (b) What do you like/dislike about it?
   (c) How long have you played it?

(d) Do you play it all the time?

(e) If not, why did you stop?

3. Appearance and type of game

(a) Do you like games with a story (GTA, War of Warcraft, Diablo, Neverhood,...) or games where an action is repeated over and over again (Angry Birds, Flappy Bird, Hungry shark,...)?

(b) What is your favourite style of game? (Action, RPG, Simulation, Sports,...)

(c) Do you prefer the game to be controlled with a joystick or just by clicking?

(d) Do you like to play alone or in a group with friends (multiplayer)?

4. Motivation

(a) Do you like to compete with your friends to see who has the best result in the game?

(b) Do you like to share your achievements in the game on Facebook?

(c) Do you like to collect points/minutes in the game to buy in-game bonuses?

(d) Which game have you played the longest and why?

(e) Is there any game you haven't played? And why?

A1.2.2 Diabetes and games

1. The first years with diabetes

(a) At what age were you diagnosed with diabetes?

(b) Where did you get your information about the disease and how to compensate for it?

(c) Was there much you had to learn?

(d) Did you forget to check your glucose meter?

(e) Do you know any other children with diabetes? If so, did you help each other?

(f) What do you think diabetes is? Could you explain it to me?

2. Diabetes today

(a) What does your normal day look like?

i. How many times a day do you eat?

ii. What sports do you do?

iii. How many times do you measure your blood glucose? iv. Do you forget to measure?

v. Have you ever had very high or low blood glucose? How often?

vi. Do you record the data in your diabetes diary or do your parents do it?

3. Diabetic games

(a) Have you ever played a game that was related to diabetes?

(b) If not, why not?

i. If you wanted to find such a game, where and how would you look for it?

(c) If yes:

i. How did you find out about this game?

ii. Did you enjoy it? Why?

iii. Did it motivate you?

iv. Did it teach you anything new?

v. What bothered you about her?

4. Diabetic apps

(a) Do you use any mobile apps - diabetes diary etc.?

(b) If not, why not?

(c) If yes:

i. what do you like/dislike about it?

ii. is there anything missing?

iii. Is there anything unnecessary?

A1.3 Summary of the individual interviews

Table S1. Summary of individual interviews

Participant	Age (years)	Sex	Gaming experience (years)	Diabetic friends	Duration (years)	Food frequency (daily)	BG measurement frequency (daily)	Insulin pump	Tools	Parents support
#1	13	m	8	4	6	6	3	no	paper diary	writing paper diary
#2	11	m	3	0	3	6	8	yes	paper diary, printing the ouput from the pump	accompanuing child to summer camp, consulting insuline injections, controling BG measurement
#3	14	m	8	once a year in a dia camp	4	6	7	yes	paper diary	controlling BG measurement
#4	13	m	na	several	7	6	2-6	yes	paper diary	at the begining insulin administration at school, paper diary filling

A1.4 Design survey

1. How old are you?
2. Gender?
3. How old have you been with diabetes?
4. Do you have a pump or pen?
5. Do you have a cell phone/tablet with an Android?
6. Which non-diabetic game do you play?
7. How did you learn about diabetes?
8. Do you know any games about diabetes?
9. Have you ever tried to play games about diabetes?
10. Are you keeping a diary?
11. Can you count BUs?
12. Can you determine the insulin dose?
13. Do you practise sports?

Table S2. Summary of design survey (quantitative research)

Participant	1. Age (years)	2. Sex	3. Diabetes duration (years)	4. Pump	5. Technology	6. Non-diabetic gaming	7. Diabetes source	8. Diabetes games knowledge	9.Experience in diabetes gaming	10. Diary	11. BUs calculation	12. Insulin calculation	13. Sports
#1	7	f	3	no	PC	puzzles	hospital	no	no	yes	yes	only lunch	cycling
#2	13	f	9	yes	-	The Sims	lectures	no	no	yes	yes	yes	trekking
#3	10	f	4	yes	PC+Android	Pou, Subway Surfers, Minecraft	parents	no	no	yes+parents	yes	yes	dancing
#4	12	f	9	yes	PC+Android	Pou, Moy, Loy, My dolphin show	parents, book	yes	no	yes	yes	no	swimming
#5	10	m	6		Android	Moy, My Talking Tom, Need for Speed	video	no	no	with parents	yes	no	floorball
#6	7	m	4	yes	Android	Mimoni, Lego Star Wars	parents	no	no	parents	no	no	running
#7	8	f	5		Android	Pou, Subway Surfers	parents	no	no	parents	no	no	yes
#8	7	f	4	yes	iOS	Angry birds, Pou	parents	no	no	parents	no	no	dancing
#9	7	f	5	yes	Android	Pou, Zombie tsunami	parents	no	no	parents	no	no	balet
#10	12	m	3	yes	Android	2048	hospital	no	no	yes	yes	yes	cycling
#11	10	f	9	yes	Android	Pou, Star girl, SpeedX	parents	yes	no	yes	yes	no	dancing
#12	13	m	10	no	Android	Shooting games	parents	no	no	yes+parents	yes	no	football, cycling
#13	9	f	7	no	Android	Subway Surfers	parents	no	no	parents	yes	no	swimming+squ ash
#14	9	f	3	no	Android	My Talking Tom, Pou	parents	no	Kompenzator	parents	yes	yes	box
#15	10	m	1	no	Android	Minecraft	hospital	no	no	no	yes	no	no
#16	13	m	7	yes	Android	GTA	video	no	no	no	yes	yes	badminton
#17	14	m	1	no	Android	Subway Surfers	hospital	no	Kompenzator	yes	yes	yes	football, cycling
#18	11	m	3	no	Android	no	hospital	-	no	parents	yes	no	badminton
#19	10	f	6	no	Android	no	parents	no	no	yes	yes	yes	no
#20	12	m	3	no	Android	no	hospital	no	some in PC	yes	yes	yes	no
#21	7	m	3	yes	Android	mobile games	hospital	no	no	parents	no	no	swimming
#22	12	f	1	no	Android	no	hospital	no	no	yes	yes	no	no
#23	9	f	1	no	PC+Android	Poe, Subway Surfers	hospital, book	no	no	yes+parents	yes	no	riding
#24	9	m	5	no	Android	Uno	parents, book	no	no	parents	no	yes	running
#25	10	m	6	no	PC+Android	Minecraft	hospital, parents	no	no	parents	no	yes	floorball
#26	13	m	11	no	Android	Counter strike, Minecrft, World of Tanks, BMX, fotbal, Hill climb ra- cing	parents	no	no	yes	yes	yes	floorball
#27	11	m	0	no	Android	Mafie, Minecraft, Call of duty, Zombie diary, Minecraft, Pou, Subway Surfers	arents, DVD, boc	no	no	yes	no	yes	inlines, footballl

In total, 27 questionnaires were collected among children aged 7-13 years. The main interest was focused on where children drew most of their information about diabetes from and if they had ever encountered a game about diabetes. Further topics of interest were about the favourite games now among the children and how independent the children are in terms of counting bread units (BU) and insulin administration. 15 girls and 12 boys completed the questionnaire. Most children got information about diabetes from their parents (15 times), the second place was occupied by the hospital (9 times), the third place was occupied by a video tutorial (4 times), followed by books (4 times) and lectures (1 time). Diabetes diaries for 11 children were filled in by parents only, 9 children filled in their own diaries, 5 children alternated with their parents and two children did not fill in diaries at all. Only four children did not own a mobile phone or tablet. Four children did not practise any sport. The most played games on mobile phones were Pou (9x), Subway surf (6x), and Minecraft (5x), four children do not play any games. Only four children knew a game about diabetes, and this was Czech online web-based educational game Kompenzator developed by Eli Lilly. Seven children did not think that a game about diabetes could exist, 4 children tried to look for a game but failed, and the rest did not look for a game for various reasons. Diabetes in this group of children was diagnosed at an average age of 5.5 years. 8 children could count the units themselves, another8 only sometimes and 10 not at all. 15 children could determine the insulin dose, 6 could only sometimes determine it and 5 could not determine the dose at all.

A1.5 Questionnaire for parents

1. Does your child have the ability to work with an Android mobile phone/tablet?
2. At what age was your child diagnosed with diabetes?
3. Do you fill in a paper diabetes diary?
4. What information do you write in your diabetes diary?
5. Does your child own a pump?
6. How many times a day do you measure a child's glucose?
7. What bothers you most about the child's disease? What limits you the most?
8. Have you ever considered using a mobile app instead of a paper diary? If not, why not?
9. What should the ideal mobile app for diabetes management do?
11. How was the education of your child and yourself? What were your biggest problems?
12. If you had the opportunity to devise an educational game for newly diagnosed children, what would it be? What would it teach the child?

Table S3. Summary of parents' responses

Participant	1. Mobile literacy	2. Age of diagnosis	3. Paper diary	4. Diary facts	5. Insuline pump	6. BG frequency	7. Concerns, limitations	8. mobile app usage	9. mobile app features	10. education	11. mobile app design
**#1**	yes	5	yes	BG, BU	yes	7	-	no, using pump software	no idea	dia-centre, internet	no idea
**#2**	yes	4	yes	BU, bolus, corr bolus, diseases	yes	5-7	pain when injecting canyle, BG night meas.	none iOS on mobile	-	real world practice	BUs recognition of food pictures
**#3**	yes	5	yes	BG, BU, Hypo, bolus	yes	7	regular food intake, hypoglycemia	no, using pump software with PC	no idea	in hospital	no idea
**#4**	yes	9	yes	BG, Insuline doses, urine (ketones), sports activity,deseases	no	7-9	stress generated by food intake control	yes, we considered	feedback about compensation	non-empatical education nurse	BUs recognition of food pictures
**#5**	no	2.5	yes	BU, BU, bolus	yes	5	BU estimation	no, using pump software, no pisiibility of inserting longer comments	List of food with BU values	in hospital, presentations in dia-centre	-
**#6**	no	2.5	yes	BG,BU, urine (ketones), regime changes, diseases	yes	6-7	fear about diabetes complications, technology maintainance	no, paper diary is OK	List of food, diary	in hospital, books, internet, regular meetings in dia-centre	Everything that can affect glycemia - insulin dose, composition and amount of food, sport
**#7**	yes	8	no	BG, insuline doses	no	5	regular food intake, insuline dose estimation	yes, we considered	Insuline dosage, food recomendation	-	Eating right, injecting the right amount of insulin

A1.6 Alpha usability testing results

Table S4. Summary of alpha usability testing

Participant	Age (years)	Sex	Duration (years)	BG meas. frequency (daily)	Insulin pump	Sports	Gaming	Tools	Diabetes Sources	Main findings: a child
#1	11	m	8	5	no	basketeball before, not now	Minecraft, My Talking Tom	paper diary by filled in parents	hospital, dia-friends ngo, dia journals	use of drag and drop gestures instead of clicking, when returning using HW button instead of dedicated button in the game, not reading help, better if MD talks, missing explanation of taking foond and applying insulin in case of low BG. Mother wants a tool for motivating daily regime.
#2	13	m	2	4	yes	tennis, golf, swimming	Minecraft, Atomas, Pou before	paper diary alone	hospital, participating in a dia summer camp	problems with lancet change and graphical visualization of the needle sharpness, missing help when measuring BG, need of meausuring character before practising sports, increasing size of food pictures, adding simulation of deseases. He was the only one to apply the insulin by a long click on the button. Mother would welcome it if even a character could be sick once in a while and his/her glycemia would rise. Also, a dubbing would have improved the game.
#3	11	f	4	5	no	athletics, basketball	Minecraft, Plants and Zombies, My Talking Tom before	paper diary alobe and by parents	hospital, internet and presentations,participating in a dia summer camp	couldn't keep up with the introductions text, drag and drop gestures instead of clicking, when shopping, clicking to food pictures, not food prizes, didn't know if the goods were already bought or not.
#4(1)	6	m	1	7	no	-	plays few games but he did not name them	-	-	missing help when measuring BG, small button to for realising the lancet, lack of feedback on the purchase of dia supplies, short of money to buy the character's clothes, but didn't notice, When buying furniture, he didn't know how to choose furniture to stay active. He didn't find the scrolling option in the food scene.
#5	9	m	4		no		War of Tanks, Pou	-	-	Suggesting a mini-game where the food would fall and one needs estimate BUs, pissibulity of byuing insuline pump or glucometer in the shop
#6	12	m	3	-	no	-	Minecraft, Weaphones, before My Talking Tom	-	-	simulation as in real life - wait at least 20 minutes after injecting the insulin, before the insulin starts to wear off and the diabetic can take food, adding sports in the form of mini-games (e.g. basketball, floorball), going to school,when returning using HW button instead of dedicated button in the game, use of drag and drop gestures instead of clicking
#7	9	m	4	-	no	-	War of Tanks, Pou	-	-	Suggesting a mini-game where the food would fall and one needs estimate BUs, pissibulity of byuing insuline pump or glucometer in the shop
#8	12	f	non-diabetic	-	-	-	jumping and logic games	-	-	She didn't understand why the insulin pen kept turning. When she found the help, she did not know how to actually turn the insulin pen. The time it takes to rinse the soap with water in the sink is too long. She didn't understand to hold the button for 5-10 seconds during isluin administration.
#9	13	f	non-diabetic	-	-	-	The Sims, Talking Tom	-	-	She could not cope well with glucose measurement and insulin administration, biiger food pictures,
#10	6	f	non-diabetic	-	-	-	Minecraft, Subway Surfers, Kitty Love	-	-	Reading text was the most difficult, she managed to read only the first two words of the introductio, problems to find the shop
#11	10	m	non-diabetic	-	-	-	Minecraft, War of Tanks	-	-	He did not know what are BUs and could not estimate them. He did not understand the meaning of the scenes.
#12	13	m	non-diabetic	-	-	-	Minecraft	-	participated in a dia summer camp	after changing the lancets in the needle, the help arrow remains above the lancets and the participant did not know what to do next, confused by the icon for going to the town with shops (door icon with an arrow), which he understood as an icon for leaving the game. Help icon for insulin could be bigger.

Feedback summary

No one had a problem with the food screen, but not everyone discovered the possibility of scrolling the panel in case there were more food items. As a result, a scroll bar that allows scrolling and visual alerts to more items was added. As far as bread units estimation is concerned, diabetics children performed better than non-diabetic children, non-surprisingly. They messed up with a maximum increase of one or two units. In contrast, non-diabetics could only estimate the visible sweet and sour food items (bonbons) from less sweet ones (chips).
In the insulin scene, most issues were caused by the removal of the paper's protective cap from the needle, which caused some users to get stuck on this step for several long minutes. Few users knew what a needle was, and even diabetic children faced the same issue. As a solution, a drag & drop gesture was incorporated. In the scene with the food purchase, some users clicked on the picture instead of clicking on the price tag. Therefore, this functionality was also added. Some of users didn't like that they couldn't choose a particular food item. This mechanism prevents repeated consumption of the same food without the need to learn about other food items.

- Objects in the glucose measurement scene only respond to clicks => allowing dragging control as well.
- In-game undo only with in-game buttons => allow undo with hardware back button.
- The game should instruct the player by voice.
- The game could offer more sports for the character.
- Apply insulin before or after meals depending on current glycemia.
- Add a spin animation when changing the lancet needle.
- Modify help after inserting the lancet into the needle to guide the player to insert the cap.
- Modify the colours to represent the sharpness of the lancet.
- Fix collision detectors between the pen and the fingers of the hand.
- Edit the icon for exiting the town with shops to not look like exiting the game.
- Add text help for measurement.
- Guide players to take measurements before practising sport.
- Simulate avatar sickness.
- Extend the time to read the introductory text.
- Edit the images in the help bubbles in the opening scene to not look like buttons.
- Edit unrecognisable food pictures.
- Improve feedback when shopping in stores.
- Enlarge the button on the pincushion.
- Add an exit button to the minigame inside the body.
- Warn about lack of money when shopping.
- Add help to set up purchased furniture.

A2. Beta Release (Iteration 2)

A2.1 Screener

1. How old are you?
    a) less than 7 years old
    b) 7 - 10 years old
    c) 11 - 15 years old
    d) more than 15 years old
2. Are you a girl or a boy?
    a) girl
    b) boy
3. Are you diabetic?
    a) yes
    b) no
    if yes, of which type?
        a) type 1
        b) type 2
4. Do you have your own mobile phone or tablet?
    a) yes
    b) no

A2.2 General questionnaire

1. How often do you play games?
    a) every day
    b) at least three times a week
    c) at least once a month
    d) at least once a year
    e) never
2. What device do you play games on?
    a) mobile phone/tablet
    b) computer
    c) console (XBOX, Wii,...)
3. When did you get your phone/tablet?
    a) when I was 5 years old
    b) when I was 6 years old
    …
    e) when I was 12 years old

4. Which operating system do you have?
   a) android
   b) iOS

5. Do you know what a tutorial is? What is the tutorial for? Have you ever played a game which had a tutorial at the beginning? Did you go through the whole tutorial, or did you have the option to skip it, and so you did?
6. Where do you play games? At school/home/car/somewhere else? Where do you obtain information about new games?
7. Since when do you play games? Which device do you use for playing?
8. Do your parents approve that you are playing games or not?
9. Do you have a favourite game? What do you like about it? Do you still play it? If not, why did you stop?

A2.3 Diabetes management

1. When were you diagnosed with diabetes? Where did you obtain all the information about diabetes from? Was it a lot of things you had to learn? Do you remember the first symptoms of diabetes?
2. Did you have a problem with forgetting glucometer measurements? Do you still sometimes forget about measurements?
3. Do you know any other children with diabetes? If yes, did you help each other?
4. Describe your daily routine
5. Do you change places of injections of insulin?
6. Have you ever had a high/low blood glucose level? How often?
7. Do you have a diabetic diary? If yes, who is managing it? You or your parents?
8. Are you satisfied with the way you can manage diabetes? Do you handle all the tasks like measuring, injecting, and dieting? What would you improve?
9. is there anything in which diabetes is limiting you? Is there anything you would like to do but cannot do?

A2.4 Pretesting knowledge of diabetes

1. Do you know what diabetes is? Can you explain it?
2. From which part of the body insulin is absorbed the fastest?
3. Describe the daily routine of the person with diabetes
4. Do you know by what blood glucose level is affected?
5. Describe the process of measuring blood glucose level
6. Describe the process of injecting insulin
7. Food.
   (a) Do you know what bread units are?

(b) Do you have an idea about how many bread units some foods have? How many bread units a day do you eat?

(c) Do you know the bread units of the following foods?

a) Apple
b) Roll
c) Spaghetti with tomato sauce
d) Egg

8. Can you define hypoglycaemia? What are the symptoms? What would you do if you had hypoglycaemia?
9. Can you define hyperglycaemia? What are the symptoms? What would you do if you had hyperglycaemia?
10. Do you know between which two values you should keep your blood glucose level?
11. When will you be able to see a change in blood glucose level on a glucometer after injecting insulin?
12. Do you adjust insulin intake when you go to exercise?
13. How do you conclude your knowledge about diabetes? Do you think

A2.5 First impression of the game

1. What is your first impression of the game? What did you do, and what didn't you like?
2. Did you understand the concept of level tasks? If yes, how does it work?
3. Did you understand that character in a game needs your help, and you cannot forget about insulin injections and food?
4. Can you read? If yes, did you look at the tutorial book? If yes, did you learn anything new?

A2.6 Questionnaire with parents

1. How is it to have a child with type one diabetes?
2. Is your life markedly influenced by that, or do you already take it as a part of your daily life?
3. How does your child psychically deal with the fact he/she is diabetic? Does she/he feel different from the others?
4. Is your child rigorous when treating the disease?
   (a) Does your child measure blood glucose level regularly?
   (b) Does she/he forget sometimes?
5. What is your opinion on playing educational games?
6. Do you think educational games could help a child learn how to treat diabetes better?
7. Where do you obtain information about diabetes? Are there enough sources?

A2.7 Beta usability testing

1. Which character did you choose? Emma or Adam?
2. How often did you play? How many times a day?
3. Did you have a sound on or off?
4. Has it ever happened that Emma/Adam had hypo/hyperglycemia? How did you react?
5. Has it ever happened that Emma/Adam was hospitalised? Can you tell me why that happened? How did you react?
6. Did you check the game settings?
   (a) did you change any values there? Which ones?
   (b)did you choose an option of a new game? if yes, why?
7. Did you visit a virtual town? What did you buy there? Did you like shopping for new clothes and furniture?
8. Did you fulfil the tasks you were given?
   (a) Was it motivating to collect more points, so you could update to a new level and buy more clothes and furniture?
   (b) What was the maximum level you got into?
   (c) Have you always had enough coins? How many coins do you have now?
   (d) Were there some tasks you were not able to fulfil?
9. Did you play minigames? Which one was your favourite?
10. Did you record any mistakes in a game? Was there something that behaved differently than you expected? What was it?
11. Have you ever got into a situation when you did not have an idea what to do next? What was the situation?
12. Have you had a look at the virtual library?
    (a) Did you read the book tutorial? If yes, did you learn anything new?
13. Did you try a glycemia simulation in a library?
    (a) Have you checked the graph of blood glucose level?
    (b) Did you understand where good blood glucose levels should lay?
    (c). Did you use a graph for estimating your blood glucose level?
14. Did you like that the written/audio help of the physician was showing up?
    (a) Did you read it/listen to it, or were you cancelling the help?
15. Was there anything that surprised you in a game? Anything you did not know about diabetes or that you have a different experience with?
16. What is your overall opinion on the game?
    (a) Specify what you liked.
    (b) What you did not like.
    (c) Was the game entertaining for the whole time? If not, when it stopped entertaining you?

A2.8 Post-testing knowledge of diabetes

1. What do you think diabetes is? How would you like to explain it?
2. Describe a series of actions that every diabetic should take within a day.
3. Food:
    (a) What are VJs?
    (b) Did you make a list of the dishes when you fed Emma/Adam?
        i. Did you remember any specific foods?
        ii. How many VJs do you have on your food list?
            A. banana
            B. broccoli
            C. bread
            D. chips
        E. bread rolls
4. How do you adjust your meal of insulin when you go to exercise?
5. What is the difference between the two types of exercise shown in the game?
6. How did the glucose change after the exercise?
7. How did your blood glucose change after food?
8. How long after the injection did the glucose monitor show a change?
9. What is your overall knowledge of diabetes now? After playing the game, did you learn anything new as a result?

A2.9 Summary of the individual interviews and beta usability testing

Table S5. Summary from the questionnaires 2.1,2.2, 2.3 and 2.6

Participant	Age (years)	Sex	Experience in mobile gaming (years)	Experience in tutorials	Diabetic friends	Duration (years)	Food frequency	BG measurement frequency	Insulin pump	Tools	Parents opinion
#1	7	m	0,75	no	no	7	6-7	6-7	no	paper diaery and after parents put it in excel	ngo Diabetes friends
#2	15	f	10	sometimes follows, sometimes skipping	5	9	5-6	2 (*)	yes	no diaery, summary provided from the pump	from facebook and diabetic community
#3	5	f	0,5	no	yes	2	na	na	yes	no diaery, summary provided from the pump	most information from literature and others diabetic mothers
#5	12	f	1	no	4	2	5	4	yes	no diaery, summary provided from the pump	from dia-styl journal and from the hospital, needs to control the child on daily basis
#6	14	f	na	yes, follows	na	3	6	4	yes	no diaery, summary provided from the pump	not presented
#7	8	f	3	yes but skipping	yes	6	6	8(2)	no	diarey using mobile app	from internet and national or even frominternational community meetings, shooting educational videos fro others

Table S6. Summary from the beta usability testing using questionnaire 2.7

Participant	Summary	Character choosen	Playing duration (mins)	Level reached	Coins balance	Virtual library	Virtual MD	Settings
#1	already participated in the prototype testing, compensated well, liked furnitureshopping	Adam	30	5	-534	no	annoying	changed weight randomly
#2	understood everything, compensated well, liked shopping	Emma (1)	20	5	1000	used frequently	followed instructions	changed weight to follow her wieght
#3	did not know how to compensate, shopped a bit	Emma	first 2 days	2	-229	no	followed instructions	changed weight randomly
#5	did not fulfill the task, did not shop, not motivated	Emma	every second day	2	280	no	not used	no change
#6	compensated well, claimed to be to big for this kind of game	Emma	every second day	9	3785	no	sometimes followed instructions	no change
#7	compensated very well, liked shopping	Emma	15	5	1000	used frequently	followed instructions	no change

A2.10 Educational effect

Table S7. Diabetes knowledge using pretesting questionnaire (2.4 Pretesting knowledge of diabetes)

Answer	Participant					
	#1	#2	#3	#5	#6	#7
**2.4.1**	0	95	10	70	90	85
**2.4.2**	50	100	100	100	100	100
**2.4.3**	30	100	80	90	90	95
**2.4.4**	60	90	20	60	60	70
**2.4.5**	70	100	80	90	90	90
**2.4.6**	30	85	0	95	90	90
**2.4.7.a**	90	100	0	90	100	70
**2.4.7.c**	0	97	0	95	100	90
**2.4.8**	90	95	5	80	90	100
**2.4.9**	90	95	5	80	90	100
**2.4.10**	0	100	0	100	100	80
**2.4.11**	0	100	0	100	100	90
**2.4.12**	0	100	60	0	0	100
Average	39,2	96,7	27,7	80,8	84,6	89,2

Table S8. Diabetes knowledge using post-testing questionnaire (2.8 Post-testing knowledge of diabetes)

Answer	Participant					
	#1	#2	#3	#5	#6	#7
**2.8.1**	50	95	75	80	90	85
**2.8.2**	90	100	90	95	95	100
**2.8.3.a**	90	100	70	90	100	80
**2.8.3.b.ii**	87	88	0	92	93	96
**2.8.4**	0	100	60	90	90	100

**2.8.5**	80	100	0	90	90	100
**2.8.6**	0	100	100	100	100	100
**2.8.7**	0	100	100	100	100	100
**2.8.8**	0	100	0	100	100	90
Average	44,1	98,1	55,0	93,0	95,3	94,6

A3. Gold release (Iteration 3)

A3.1 Pretesting questionnaire

1. Age?
2. Gender?
   (a) Girl
   (b) Boy
   © I don't want to specify
3. Do you have diabetes?
   (a) Yes, Type I.
   (b) Yes, Type II.
   © No
4. If yes, at what age were you diagnosed (diabetes)?
5. If you have diabetes, do you use an insulin pen or pump?
   (a) Pen
   (b) Pump
6. Do you own a smart phone?
   (a) Yes
   (b) No
7. Do you use a smartphone?
8. If yes, Android or iOS?
9. Approximately how old were you when you started using a mobile phone?
10. Do you play games on your mobile phone?
   (a) Yes
   (b) No
11. Which platform do you prefer for gaming?
   (a) Mobile phone
   (b) PC
   © Game consoles – PlayStation, Xbox, etc.
   (d) I don't play video games
12. How often do you play games on your mobile phone?
   (a) I don't play
   (b) 1x – 3x per month
   © 1x – 3x per week
   (d) Every day or almost every day
13. Have you played any game that was for diabetics or with this theme?
   (a) Yes
   (b) No

14. What type of game do you like to play? (You can mark more than one)
    (a) Adventure games
    (b) Action games
    © Jumping games
    (d) Puzzle
    © Simulators
    (f) Sports games
    (g) Other (Specify which
15. Where do you most often play games?
    (a) At home
    (b) At school
    © On the move (public transport, car, train. . . .)
    (d) Elsewhere
16. How do you find out about new games?
17. How old have you been playing games?
18. Do your parents approve of you playing games?
19. Do you know what a game intro/tutorial is?

Table S9. Summary from questionnaire 3.1

Participant	1.Age	2.Gender	4.Dia duration (years)	5.Pump	6. Smartphone use	8. OS system	9. Since when	10. Playing on phone	11. Gaming platform	12. Frequency playing (weekly)	13. Diabetic game	14. Type of game	15. Where playing	16. Where learned	18. Parents agreement	19. Tutorial
#1	13	m	7,5	yes	yes	-	8,5	yes	mobile phone, gaming console	7	no	adventure, action, jumping, logic, simulaters	home, travelling	-	yes	yes
#2	13	m	2	no	yes	Android	5	no	PC, gaming console	1	no	adventure, action, strategies	home, travelling	youtube, internet	yes	yes
#3	14	m	13	yes	yes	-	8	no	PC	1	no	action	home	youtube	yes	yes
#4	14	f	6	yes	yes	Android	10	yes	mobile phone	2	no	action, jumping	travelling	friends	not really	yes
#5	14	m	9	yes	yes	Android	8	yes	PC	7	no	action, simulation	home	internet, TV	I don't know	yes
#6	16	f	7	yes	yes	iOS	8	yes	PC	2	no	logic, simulators	home	friends, internet	I guess so	yes
#7	15	m	6	no	yes	iOS	-	yes	All identically	2	no	adventures, action, simulators	home, elsewhere	News	yes	yes
#8	13	f	8,5	no	no	-	10	yes	mobile phone	1	yes	simulators	home	friends	yes	yes
#9	8	f	3	no	no	-	-	no	-	-	no	-	-	-	-	-
#10	15	f	10	yes	yes	Android	10	no	PC	1	no	adventure, sport	home	friends	no	yes
#11	10	f	5	yes	yes	Android	6	yes	mobile phone, gaming console	7	no	all types	home	ads	yes	yes
#12	7	m	7	no	-	-	7	-	-	7	no	action	home	internet	no	no
#13	12	f	4,5	no	yes	iOS	-	no	gaming console	1	yes	adventure, action,jumping, simulators, sport	home	friends	yes	yes
#14	15	f	6	no	yes	Android	11	yes	-	2	no	adventure, jumping, logic, sport	home, travelling	ads, youtube	no	yes
#15	14	m	6	no	-	-	11	-	-	2	no	action, logic, sport	home	internet, friends	yes	yes
#16	14	m	8	no	yes	iOS	8	yes	PC	7	no	action	home	internet	yes	yes
#17	13	f	10,5	yes	yes	Android	9	yes	mobile phone	2	no	action	home	friends	sometimes	yes
#18	12	m	8	no	yes	Android	8	yes	PC	7	no	action	home	internet	yes	yes
#19	14	f	8	no	yes	iOS	6	yes	gaming console	2	no	adventure, action , jumping, simulators, sport	home	friends	yes	yes
#20	13	f	12	no	yes	Android	10	yes	mobile phone, gaming console	2	yes	sport	home, school	friends	yes	yes
#21	15	f	10	no	yes	Android	-	no	-	not playing	no			not playing	not playing	yes
#22	12	f	4	no	yes	iOS	10	yes		2	yes	sport	elsewhere	friends	yes	no
#23	13	f	11	yes	yes	Android	7	yes	mobile phone	2	no	simulators	home, travelling	play stores	no	yes
#24	15	f	8	yes	yes	Android	10	no	gaming console	1	no	simulators	home	school	yes	no
#25	13	m	4	no	yes	Android	5	yes	gaming console	7	no	adventure, action cubicles, simulators, sport	home, travelling	internet	yes	yes
#26	11	f	8	yes	yes	Android	7	yes	-	2	no	sport	travelling	google play	Yeah	yes
#27	13	m	9	no	no	Android	10	yes	mobile phone, PC	7	yes	tycoon	home	google play	yes	yes
#28	10	f	8	yes	yes	Android	8	no	playing little	2	no	action	playing little	I'm not looking	playing little	yes
#29	14	f	9	yes	yes	Android	11	yes	mobile phone	7	no	logic	home	internet	yes	yes
#30	14	m	0	no	yes	Android	8	yes	PC	7	no	adventure, action, jumping, sport	home	friends on social networks	yes	yes

The mean duration of diabetes was found to be 7.3 years. Of the total participants, 17 reported using an insulin pen while 13 used an insulin pump. Concerning the mobile operating systems used, Android was the preferred choice for 18 participants, while 6 used iOS and 3 individuals other operating systems, respectively. Three participants did not use a smart mobile phone. The average duration of mobile phone usage among the

participants was 4.6 years. In terms of gaming devices, 9 participants used a personal computer, 7 exclusively used a mobile phone, and 9 used a gaming console. The mean frequency of gaming was 3.6 times per week. Among the participants, 5 played an educational game that focused on diabetes. With regards to the types of games played, 10 participants played adventure games, 17 played jumping games, 6 played logic games, and 10 played sports games. Only three parents expressed disagreement with their children engaging in game playing. Among the sources of information, 13 participants received game recommendations from friends, 10 found games through internet search, 4 from a YouTube channel, 3 from Google Play, and 2 from advertisements. Finally, three participants reported never using the tutorial.

A3.2 CGM and insulin pump module: post-testing questionnaire

1. If you use a CGM sensor, what type?
2. If you use an insulin pump, what type?
3. What did you like about the CGM sensor application?
4. What did you dislike about the CGM sensor application?
5. What did you like about using the CGM sensor?
6. What did you dislike about operating the CGM sensor?
7. What did you like about the application of the insulin pump infusion set?
8. What did you dislike about the application of the insulin pump infusion set?
9. What did you like about operating the insulin pump?
10. What did you dislike about operating the insulin pump?
11. Is there any aspect of life with a CGM sensor or insulin pump that is missing from the game?
12. Did you like the game? What did you like, what did you not like?
13. Did you have any problems with anything in the game?
14. What could be improved about the game?

Table S10. Summary of CGM/Pum module testing based on questionnaire 3.2

Participant	Age (years)	Gender	Duration (years)	Pump	Pump/CGM	Mobile phone ownerhsip	Gaming platform	Desciption of participant experience
#25	13	m	4	no	-	6	console	He had no problems with the basic game mechanics and found the game easy to navigate. However, he had trouble with movement when connecting the infusion set and reservoir with the tubbing.In the same scene, he also found an error causing the steps 2-4 to be rearranged and skipped during the infusion set application. As one of the few, he came up with the idea of measuring his glycemia with a glucometer before calibrating the sensor.
#28	10	f	8	yes	Dexcom/ Dana RS	-	-	She had a problem assembling the insulin pump, specifically connecting the infusion set to the reservoir using a tubbing. Similar to the first tester, Similar to the first participant, she discovered an error causing her to skip the steps to introduce the infusion set. While playing the game, she had trouble with dialog boxes that appeared in different parts of the game and got stuck for quite some time in the scene with food
#26	11	f	8	yes	Medtronic/ Medtronic	-	-	She quickly got oriented in the game. She applied the glycemia sensor without any problems, but similarly to the previous testers, she had trouble connecting the infusion set to the reservoir with the tubbing. After receiving a hint, she managed to pull out the tubbing, but had difficulty finding the place to connect it. During free play, she preferred using the glucometer and insulin pen over reading data from the sensor and pump
#13	12	f	4	no	No/ Enlite (Medtronic)	3	mobile	She had no problems with applying the sensor. During the sensor calibration, without any advice, she started by measuring glycemia using the glucometer. She had trouble pulling out the tubbing when assembling the infusion set, but after pulling it out, she easily connected it to the set. When administering insulin, she did not know how many units to enter.
#27	13	m	9	no	no/ Free Style Libre	5	mobile	He had trouble with the gestures for pulling, the applicator, and the disinfectant wipe when applying the sensor. He had problems assembling the insulin pump. He couldn't connect the reservoir to the infusion set and push the air out of the tubbing. He got stuck during sensor calibration and had to be advised that it is necessary to measure glycemia using a glucometer first. When administering insulin, he had difficulty determining the correct amount. During the game, he had trouble distinguishing between different types of food.
#20	13	f	12	no	no/ Free Style Libre	-	mobile	She was confused during the sensor application. She had trouble preparing the applicator. During the infusion set application, she successfully pulled the tubbing out of the reservoir, but could not find the connection to the infusion set. For the rest of the game, she was able to orient herself well
#30	14	m	0	no	no/ Dexcom G6	6	mobile/PC	He did not have any problems with the sensor application. He struggled with assembling the insulin pump and needed guidance on connecting the reservoir to the rest of the pump using a tubbing. He also had difficulty with the sensor calibration and needed to be told how to measure his current blood glucose level
#21	15	f	10	yes	Medtronic /Medtronic	-	mobile	She had no problems with applying the sensor. Before calibration, she measured her blood glucose level on her own. She managed to assemble the pump and remove the air from the tubing without any help. She had some trouble navigating the scene with food in the game, mistaking the purchase button for the list of meals.
#22	15	f	8	yes	Medtronic /Medtronic	-	consosole	At the beginning, she was confused during the application of the sensor. Then she started to follow the arrows. During the sensor calibration, she needed guidance to measure her blood glucose using the pen. When applying the insulin pump, she had trouble pulling out the tubbing and pushing out the air. It was necessary to guide her through the correct gestures. She was also confused during the rest of the game, and it was necessary to perform all necessary activities.

A3.3 Glucagon module usability testing

The glucagon application module was tested by 11 participants, consisting of 7 girls and 4 boys. Prior to testing the module, participants were asked a series of questions to assess their knowledge of fast-acting sugars:

Pretesting questionnaire on glucagon use

1.Are you familiar with glucagon?
2.Do you know when it is necessary to use it?
3.Do you have glucagon with you?
4. Have you ever administered glucagon before?

Tasks to perform

1. Measure your sugar levels
2. Overdose on insulin
3. Speed up time and wait until you get hypoglycemic
4. Take your blood sugar. How high is it?
5. Use glucagon.
6. Speed up the time and wait until you get back to normal.

Table S11. Summary of Glucagon module testing. *Participant #21 also participated in CGM module testing. **Participants #16, #18 decided to test the module functionality together.

Participant	Age (years)	Gender	Duration (years)	Pump	Glucagon experience	Desciption of participant experience
#15	14	m	6	no	lacked knowledge on proper usage.	Despite not owning a smart phone, he intuitively understood how to play the game. He had no problem with measuring his blood sugar, but was unable to press the button on the insulin pen in the insulin application panel. He knew which part of the body was best to inject long-term insulin and where to inject short-term insulin. In the glucagon injection scene, he wanted to shake the vial, but immediately realised that he could only shake the syringe. He spent more time in this scene and after testing said he liked this scene the best.
#12	7	m	-	no	-	Due to age, the tester was stressed. He was not very good at operating the mobile due to his lack of experience with the touch screen. He dragged most of the objects in the game with his finger, which did not always work because some had listeners set to click only. Still, he managed to complete all the tasks in the end. Like the first participant, he couldn't hit the button on the insulin pen.
#11	10	f	5	yes	knew about glucagon and had it with her, but never used it	The tester demonstrated a rapid understanding of the game controls. However, during a hypoglycemic episode in the game, she attempted to wash her character's hands, which is not permitted in the game due to the urgency of severe hypoglycemia and the possibility of administering glucagon through clothing. During the glucagon module, the tester also attempted to remove the syringe and vial from the box, which was not necessary for successful completion of the task.
#13*	12	f	4,5	no	knew about glucagon and had it with her, but never used it	Even though she doesn't play mobile games, she was able to do the tasks quickly and understood what she had to do without any help. The problem occurred in the insulin application scene with the small area of the button to press the plunger of the syringe. In the glucagon application scene, she tried to remove the protective wrapper by dragging, instead of the implemented click.
#16/#18**	14/12	m/m	8/4	yes/ yes	both knew about glucagon and had it with them, but never used it	The boys tested the game together and completed the tasks without difficulty. Similarly to other participants, they struggled with pressing the button on the insulin pen and attempted to drag objects with their fingers. They were also unsure about the rationale for injecting long-acting insulin into the buttocks.
#19	14	f	8		knew about glucagon and had it with her, but never used it	Considering her age, the participant managed the game with ease and completed all tasks without any difficulties. During the testing she encountered the same shortcomings as the previous testers, but immediately understood the principle of dragging and clicking.
#14	15	f		no	knew about glucagon and had it with her, but never used it	The 15-year-old tester performed well in the game as expected.

Summary of the main UX findings

- Opening the box

When the glucagon box and syringe appeared on the screen, the children were able to understand that they needed to click the box to open it. However, many of them attempted to pull out both items from the box, which was not possible in the scene. To address this expectation, changes were made in the scene (see Figure S7B).

- Preparing the solution

The scene displayed a syringe with a protective wrapper and a vial of glucagon hormone powder. However, some of the children attempted to remove the protective wrapper by dragging instead of clicking with animation. The same issue arose with injecting the aqueous solution from the syringe into the glucagon vial. Despite this initial confusion, the children quickly grasped the principle of clicking and were able to complete the task successfully.

- Shaking

After the solution was prepared, the vial required shaking, and the task was simplified by adding a particle system with a flickering light effect , which was well received by the children. However, during the shaking phase, the children instinctively attempted to shake the vial, which was not the intended feature as it was added to the syringe. To accommodate the majority's preference for holding the vial instead of the syringe, corresponding adjustments were implemented.

- Drawing the solution back into the syringe and then removing the syringe from the vial

It was observed that the children had a preference for the more realistic action of pulling instead of a simple click. This was taken into consideration and applied to a different solution for both pulling the solution back into the syringe and removing the injection from the vial.

- Glucagon application

The actual application of glucagon via syringe into the thigh muscle of the physique is depicted in this scene. As with the previous cases, the finger-pulling motion was preferred by the children. This scene, along with the third one, is enjoyed by the children and is very popular among them.

- Reward

A reward dialog appeared in the form of receiving coins upon successful completion, as with the other scenes.

A3.4 Ketoacidosis module usability testing

Pretesting questionnaire on ketoacidosis knowledge

1. Do you know what ketone bodies are?
2. Do you know what ketoacidosis is?
3. Do you measure ketone bodies in your body?
    (a) Yes, from the urine using the diagnostic paper
    (b) Yes, from the blood using a device that's designed for that purpose.
    (c) Yes, from breath
    (d) No
4. If you measure your ketone bodies, how often?
    (a) Never or less than once a month
    (b) Rarely (about 1 – 3 times a month)
    (c) Occasionally (about 1x – 3x per week)
    (d) Every day or almost every day
5. Did you know that during illness (flu, viral illness, seasonal infectious diseases, etc.) a diabetic's insulin dose may change?
6. Do you know how you should behave during illness (flu, viral illness, seasonal infectious diseases) ?

Tasks to perform

1. Go to the bathroom and measure to Adam/Emma ketones in urine.
2. Stop administering insulin to Adam/Emma, repeat measuring ketones several times and observe how their levels increase.
3. Take care of Adam/Emma and play into the second day of the game.
4. On the second day, Adam/Emma gets sick, follow the regime that the physician in the game will specify.

Table S12. Summary of Ketoacidosis module testing. *Participant #21 also participated in CGM module testing.

Participant	Age (years)	Gender	Duration (years)	Pump	Glucagon experience	Desciption of participant experience
#9	8	f	3	no	The girl occasionally measures her ketone bodies using diagnostic paper strips from her urine, but her parents decide when to measure them. The girl herself doesn't know what ketone bodies or ketoacidosis are. She knows that when she gets sick, she must change her insulin dosage and daily routine after consulting with her doctor.	During testing, the girl struggled to navigate the game, but she had no problem finding the scene for measuring ketone bodies, and she was able to complete the whole scene successfully. After that, she stopped giving insulin to the character and repeated measuring her ketone levels several times during the day, which she was able to do successfully each time.
#8	13	f	9	no	Although she thought she knew what ketone bodies were, she later identified them as sugars, which is not true. She measures ketone bodies 1-3 times a month using diagnostic strips from urine, and she has a rough idea of what ketoacidosis is.	The player was able to measure the amount of ketones in the blood several times, and an increase in the ketone level was recorded when administering insulin, although there was an error that caused the time acceleration button to get stuck. As the game progressed to the second day, thecharacter fell ill.
#6	16	f	7	yes	The girl knew basic information about ketones and ketoacidosis, she measured her ketone levels herself using diagnostic urine strips a few times a month. She also knew how to behave in case of illness.	The girl was successful in measuring her ketone levels and increasing them by not administering insulin. She took care of her character until the next day, but she tried to overfeed him in order to achieve a very high blood sugar level, which didn't increase significantly. On the next day of the game, the avatar became ill and the girl continued to take care of him.
#5	14	m	9	yes	Theboy has heard of ketones and ketoacidosis but cannot define these terms precisely. He measured his ketones very rarely using diagnostic strips from urine. He had enough information about how to behave during an illness.	The boy measured his urine ketone levels several times during testing, but because the player continuously administered insulin to the avatar, the result on the diagnostic strip was always negative. During the testing, the boy did not want to complete the assigned tasks, but only explored the boundaries of the MyDiabetic game, subjecting the avatar to extreme conditions. Since this boy was already on the edge of the target group for which the game is intended, it was clear that he did not see it as an educational tool, but only as entertainment. Nevertheless, the players managed to progress to the next day in the game, where the avatar became ill, but the player was not interested in further care for the avatar.
#3	14	m	13	yes	He had a superficial knowledge about ketone bodies and ketoacidosis, but he measured his ketone levels in urine a few times a month using testing strips. He knew that in case of illness he should contact a doctor to establish a treatment plan.	The participant was able to measure the amount of ketones without any problems several times, but he became interested in the game and tried to explore its functions, so he forgot that he shouldn't administer insulin, and therefore he was unable to record an increased level of ketones. He also did not participate in the next testing day because he wanted to explore the game more and not complete tasks.
#23	13	f	11	yes	The girl had good knowledge about ketones, ketoacidosis, and also knew that during illness she must adjust her regimen after consulting with her doctor. She stated that she measures her blood ketones using a special glucose meter whenever she has high blood glucose levels.	The girl was successful in measuring ketones and then observing the color change of the strip after not applying insulin for several hours. In the first part of the scene where she opened the box of strips, the girl was impatient and clicked on the lid during the animation when it automatically moved to its place, causing the scene to get stuck. However, after reloading and removing the lid in the scene, everything worked again. In the next game day, the avatar became ill and the girl understood that she needed to take care of him more.
#21*	15	f	10	no	The girl knew the essentials about ketones, ketoacidosis, and how to behave during an illness. She measured her ketone levels from urine using diagnostic strips.	The participant also successfully measured ketones from her urine without any problems. Unlike other participants, she did not want to explore the game but only tried to quickly complete the assigned tasks. In the bathroom scene, she had no trouble finding the correct icon for measuring ketones. An increase in ketones in urine was visible when insulin was not administered.

A4. CGM and insulin pump implementation

A4.1 CGM model description

The CGM sensor is a standard subcutaneous sensor that realistically measures glucose concentration in the interstitial space. The BG and active blood insulin waveform can be extracted from the AiDA model that was implemented in the game. To obtain the interstitial glucose (IG), we need to model the two effects that the BG – IG dynamics introduce into the measurement of glucose by the subcutaneous CGM sensor, namely the delay and the absolute value. At the same time, it is necessary to remember the influence of calibration, that can reduce the delay and practically remove the difference of the absolute value.

Calibration of the sensor consists of measuring the glycaemia (using a glucose meter) and entering this value into the CGM sensor management. During calibration, the so-called calibration quality is calculated, which is obtained using the following formula:

$$k = \max\left\{0; \frac{9}{10} - \frac{1}{1 + |G_k - G_r|}\right\} \qquad (4.1)$$

where k is the quality of the calibration, G_k represents the glycaemia applied for calibration and G_r corresponds to the actual glycaemia. This metric ranges between 0 to 1, whereby a lower value indicates superior calibration. A value of 1 is assigned to an uncalibrated sensor. However, even if the calibration process incorporates a glucose measurement with an error less than 0.1 mmol/l, the quality k remains at 0. Additionally, the quality of calibration is observed to decrease with increasing calibration age, as tabulated in Table 4.1.

Table S13. Decreasing calibration quality

**calibration age (h - hours)**	**effect**
0-10	*k* remains constant
10-12	*k* increases at a rate of 0,05/h to a maximum value of 1
12 and more	*k* increases at a rate of 0,1/h to a maximum value of 1

The value of k is reflected in two ways: in the calculation of the noise and inaccuracy rate due to sensor desensitisation. The total error due to poor calibration is calculated using the following equation:

$$\hat{G}(k, G, t) = k \cdot \sin(t\frac{2\pi}{60}) + \begin{cases} (4,5 - G)k & G \leq 4.5 \\ (G - 7,5)k & G \geq 7.5 \\ 0 & jinak \end{cases} \qquad (4.2)$$

where *t* is the current time in minutes, *G* is the measured glycaemia without error. The first part of the equation simulates the error due to noise. The noise is modelled using a sine function with a period of 60 minutes. From the second part of the equation, we see that at higher glycemia, poor calibration will cause a lower value to be measured and at lower glycemia will cause a higher value to be measured.
The actual glycaemia value measured by the CGM sensor is calculated using the following formula:

$$G_{CGM}(k,t) = G(t-\tau) + \hat{G}(k, G(t-\tau), t) \qquad (4.3)$$

where the function *G(t)* describes the glycaemia time course and τ is the applied delay. In the model, τ =*7 mins.*

In addition to the current value, the sensor also evaluates the glucose trend. The trend is determined by the difference between the current value and the value 15 minutes ago. According to Table S14, there are 7 different trends. The cut-off values are determined by commercial CGM sensors [11,12].

Table S14. Trend determination

**Glycemia change (mmol/l/min)**	**Trend**
-0,167 and less	very rapid descent
-0,167 ... -0,111	rapid descent
-0,111 ... -0,056	slow descent
-0,056 ... 0,056	constant
0,056 ... 0,111	slow climb
0,111 ... 0,167	fast climb
0,167 and more	very fast climb

A4.2 CGM management

For the CGM sensor management, 3 scenes are designed: sensor application, sensor calibration and data reading. The sensor application procedure is described in Table S15 and illustrative images are shown in Figure S4.

Table S15. Application steps for the continuous glucose monitoring sensor

Step	Taks	Gesture description
1	unpack the package	click the box containing the sensor, patch and disinfectant gauze
2	charge the applicator	drag the applicator into the sensor box
3	select the application site	click the selected area on the body
4	disinfect the site	drag the disinfectant gauze over the skin
5	apply the sensor	drag the applicator onto the skin
6	fix the sensor	pull the patch over the sensor

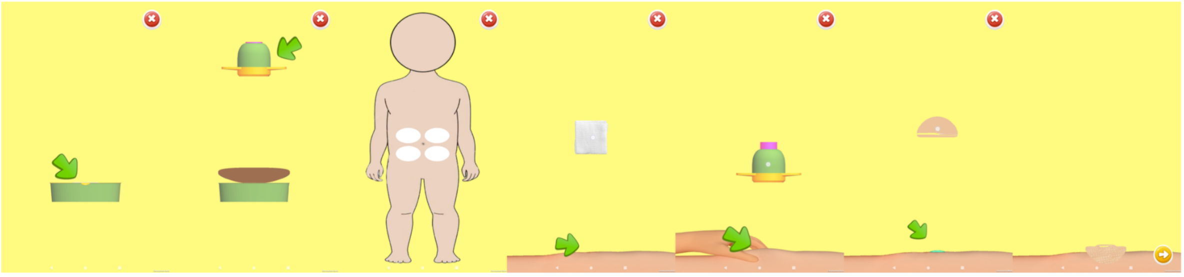

Figure S4. The process of applying a continuous glucose monitoring sensor typically involves several sequential steps, including opening the sensor package, charging the applicator, selecting an appropriate insertion site, disinfecting the site, starting the applicator, and finally securing the sensor in place.

A4.3 Insulin pump & CGM model description

To simulate interstitial glucose (IG) measurement, two effects that impact the dynamics of blood glucose (BG) need to be modelled. These effects are the delay and the absolute value associated with using a subcutaneous CGM sensor. Additionally, the influence of calibration must be considered, as it can reduce the delay and practically eliminate the difference in absolute value between CGM readings and actual blood glucose levels. In the game, the insulin pump featured represents a fundamental type of pump that utilizes a subcutaneous insulin infusion, delivered in small doses at 10-minute intervals. This device receives data from a continuous glucose monitoring (CGM) sensor (Figure S7A) and automatically halts insulin infusion if an impending hypoglycemic event is detected. The detection of hypoglycemia risk is determined through linear extrapolation of the glycaemic trends over a 10-minute interval.
The user is responsible for manually entering the size of the insulin bolus dose, which is administered as a single dose. In response to food intake, the insulin pump suggests a recommended bolus dose, which varies based on the type of food consumed. In the event of

hyperglycemia, the player is presented with a compensatory dose, calculated using a predetermined formula:

$$I = \frac{G - G_{ref}}{C} \qquad (4.4)$$

where *I* is the number of insulin units, *G* and G_{ref} is the current and reference glycaemia, *C* is the insulin sensitivity.

A4.4 Insulin Pump Management

Two distinct scenes were developed to facilitate interaction with the insulin pump. The initial scene focuses on the introduction of a new infusion set. The list of application steps is given in Table S16 and the flow is shown in Figure S5. The models used in the scene and the principle of the application are inspired by Medtronic MiniMed 640G (refer to Figure S6).

In the second scene, the insulin infusion scene (depicted in Figure S6), the player is presented with the recommended bolus dose of insulin. This is calculated as the sum of the insulin needed to compensate for the next main meal and the insulin needed to correct for any current hyperglycaemia. Additionally, the scene displays the amount of insulin that has already been injected, which is reset to zero before each main meal. Finally, there is a text box where the player can manually enter the desired insulin dose to be injected.

Table S16. Steps for insulin pump application

Step	Taks	Gesture description
1	unpack the package	unpack the package and click on the box that contains the infusion set, patch, and disinfectant gauze
2	assemble the pump	pull the end of the tubing from the reservoir towards the infusion set
3	squeeze the air out of the tubing	hold down the button on the reservoir until drops of insulin begin to flow from the infusion set
4	charge the applicator	click on the top of the applicator
5	remove the needle cover	click on the needle cover to remove it
6	select the application site	click on the selected area of the body to insert the infusion set
7	disinfect the site	drag the disinfectant gauze over the skin
8	attach the applicator	drag the applicator over the skin
9	start the applicator	click on the applicator button
10	attach the infusion set	pull the patch over the infusion set

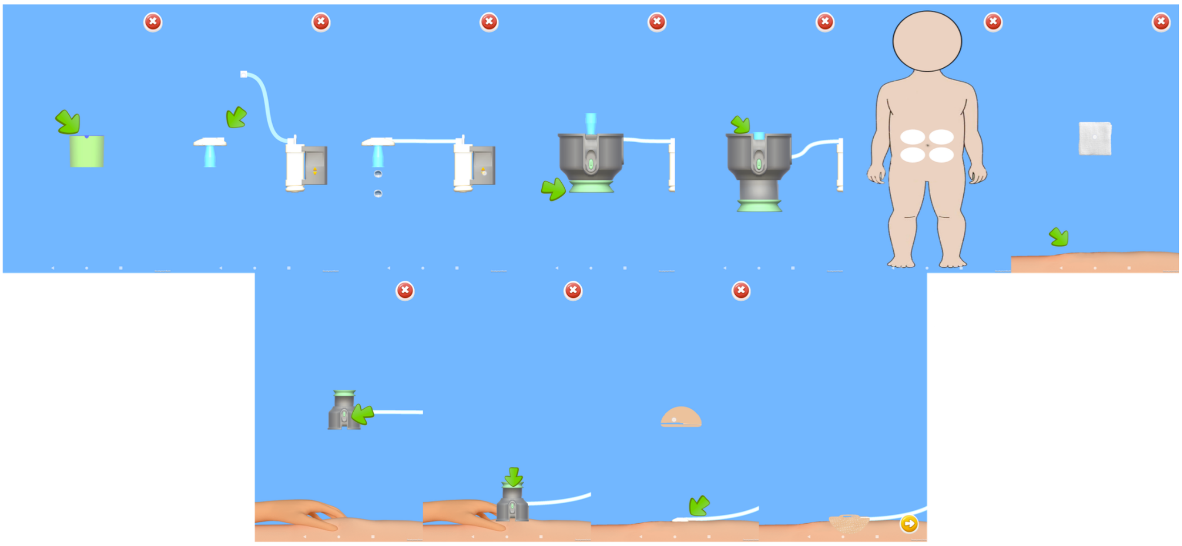

Figure S5. Insulin pump application. Opening the package, assembling the pump, displacing the air, charging the applicator, removing the needle cover, selecting the site, disinfecting the site, attaching the applicator, starting the applicator, and securing the infusion set.

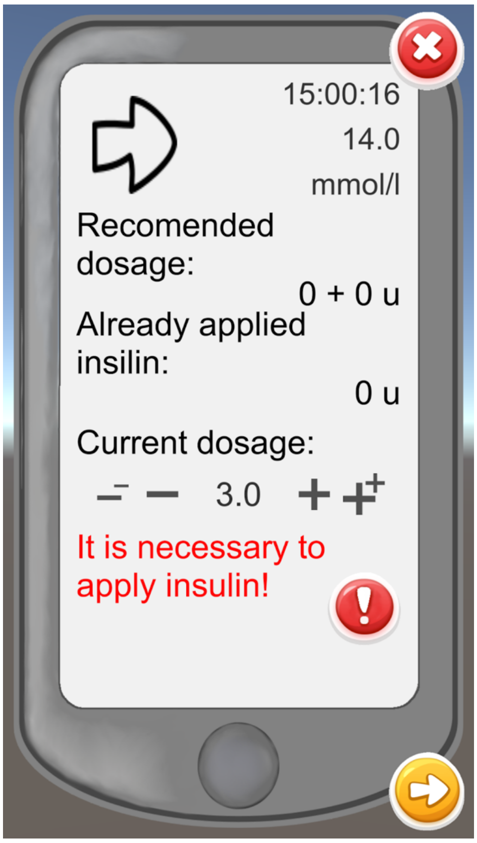

Figure S6. Insulin infusion scene

Regarding the insulin pump, there are several issues that need to be considered, such as disconnecting the pump before entering the water, the risk of the cannula breaking, the

infusion set being torn out, the CGM sensor being lost during rapid movement, and the refilling of the insulin pump reservoir. Several children expressed interest in the ability to test ketone bodies from urine and blood or breath. It would be beneficial to include the option to measure ketone bodies using a special glucometer in the future, which could be made available in more advanced levels of the game to encourage continued engagement. This could also ensure clarity between measuring ketone bodies and glucose from the blood, which are very similar and use the same device but different test strips. To address the issue of glucagon administration, when children attempt to take the bottle and syringe out of the bottle prematurely, it may be helpful to add an option to remove items from the box in the game. This could prevent confusion and ensure children follow the proper steps for administering glucagon. Additionally, incorporating clear instructions and visual cues in the game could help reinforce the correct procedure for using the glucagon kit. Finally, to improve the game's playability, it would be beneficial to automate frequently performed actions and speed up their execution.

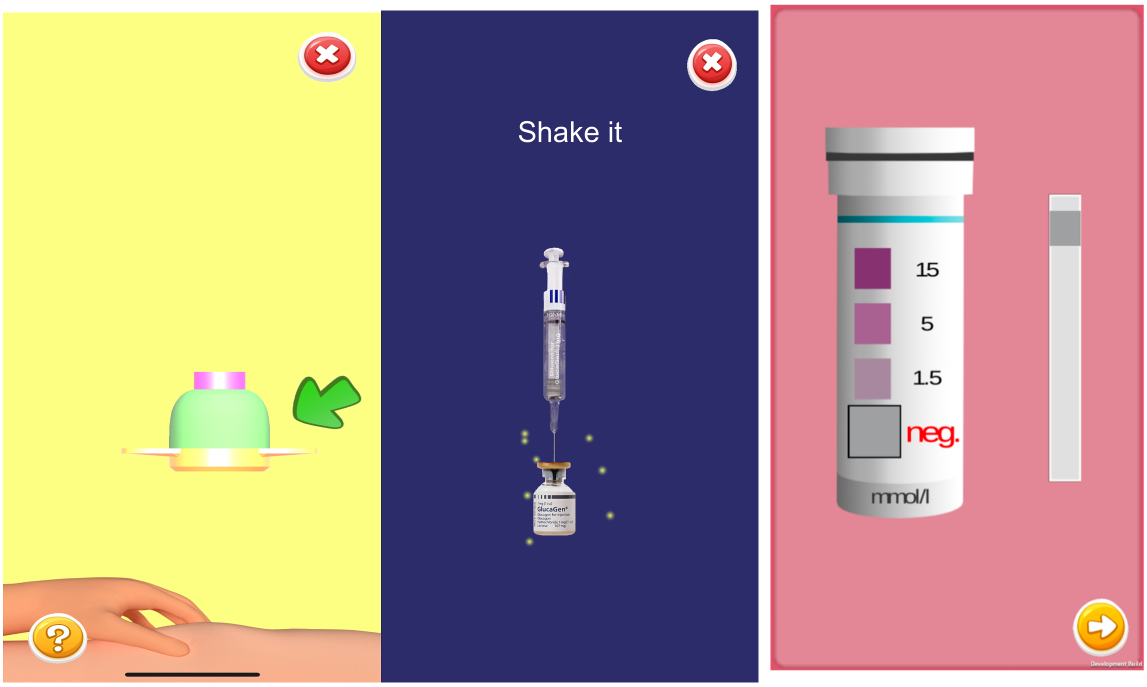

Figure S7(a) CGM sensor implementation, (b) glucagon injection, (c) ketoacidosis diagnostic kit

A5. Ketoacidosis module implementation

A5.1 Model of ketones

The kinetics of ketone body production in the human body depends on a large number of factors. However, accurately describing this process mathematically in diabetic adults and children is a challenging task. Conducting a clinical study that would map changes in ketone bodies in a diabetic patient who skips insulin application over an extended period could endanger their health and worsen their overall condition. Nevertheless, a study was conducted in 2006 [13] in which seven patients with T1DM, who were being treated with continuous subcutaneous insulin infusion, were disconnected from the insulin pump for four hours after an overnight fast. During this time, capillary β-hydroxybutyrate concentrations were measured nine times. The data obtained from the measurements was normalized with respect to the weighted mean of isochronous values, and the profile was then approximated from these non-dimensional data using a linear mathematical model.

$$dKB\frac{dKB}{dt} = K_k(I_{KB0} + I_{KB}) - C_k KB \qquad (5.1)$$

where *KB* is capillary concentration of ketone bodies, I_{KB0} is initial insulin concentration I_{KB} is insulin concentration, K_k is ketone body production coefficient and C_k = coefficient of clearance.

Thanks to the experimental model, the coefficients K_k and C_k were determined so that the *RMS* error was minimal:

$$C_k = -0.02h^{-1} \qquad K_k = 0.42h^{-1} \qquad (5.2)$$

$$RMS_{err} = 0.013$$

Using the estimated coefficients, equation 5.1 is finally reduced to

$$\frac{dKB}{dt} = 0.41(I_0 - I) \qquad (5.3)$$

where *I* is plasma insulin and I_0 is initial plasma insulin value.

This mathematical model is only an approximation, but sufficient for the purposes of the game. It is important to also realize that the body's reaction to missed insulin in patients treated with insulin pumps is different from those who only administer insulin several times a day. Several

studies indicate that many small doses of rapid-acting insulin in continuous subcutaneous insulin infusion are associated with faster and greater metabolic deterioration after interruption of insulin supply [13]. The model was also created based on the measurement of capillary ketone concentrations, while ketones reach the urine with a delay.

The model was also created based on the measurement of β-hydroxybutyrate, while in urine, we can only measure acetoacetate and to a limited extent acetone. Since the ratio between acetoacetate and β-hydroxybutyrate can vary greatly, there may be a situation where measuring ketones in urine will only show a low concentration, while in the body, there will be a high concentration of β-hydroxybutyrate, leading to acidosis. These discrepancies are highly individual and depend on many factors. Despite these deviations, this mathematical model was used because it is important in the game to show the relationship between missing insulin and the increase in ketones. The reference value for ketones in the case of zero insulin deficiency in the character will be 0.2 mmol/L. This value was chosen based on the assumption that the normal value of ketones in the blood is less than 0.5 mmol/L [14]. The design of the ketoacidosis administration is depicted in Figure S7C.

A6. Future work

Make the game feel more real-life, and users can learn to manage diabetes bette**r.** This can be done by:

- Having the avatar age over time.
- Including everyday activities like school, work, sports, socializing, and entertainment by integrating more storylines (Figure 8AB).
- Simulating possible complications of diabetes, like vision problems or foot issues (see Figure S8C).

By mirroring real life, the game can give users a more well-rounded experience that helps them prepare for managing diabetes in their own day-to-day routines.

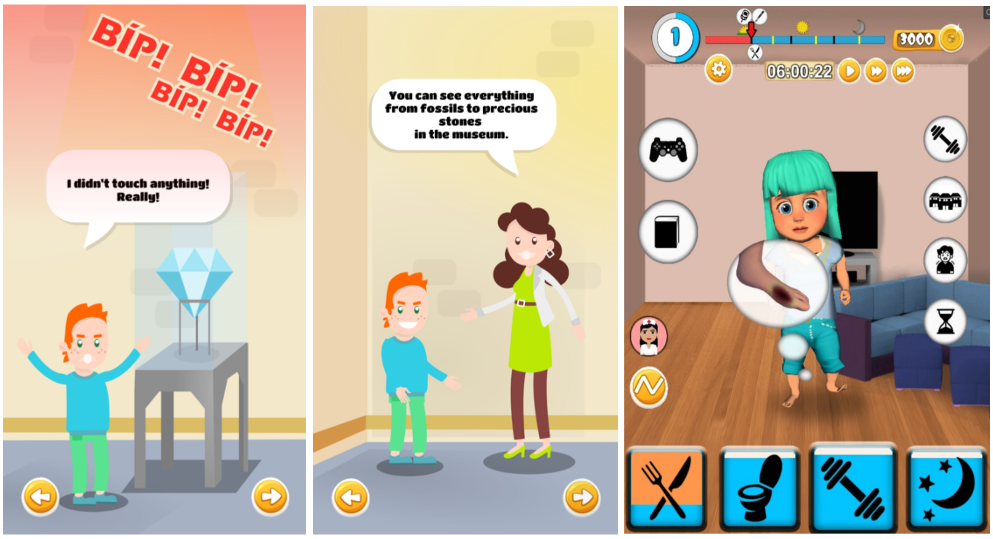

Figure S8. (a,b) storyline example (c) diabetes complications: diabetic foot

A7. Tutorial description

The first time the user turns on the game, it is advisable to introduce the game mechanisms in some way. Since children are the primary audience for this application, it is better to use the visual form instead of the written form. This is done by means of a 'tutorial' in which the player should learn the essential part of the game mechanisms, the meaning of the game and learn to control the game. In the tutorial, the player is only able to perform a given step, through which he should learn to control the game correctly. The tutorial was divided into 31 sections, which are listed below. The tutorial scenes are depicted using state automat method in Figure S10.

1. The first screen with the year of birth of the player (you can leave the year blank), including a link to the GDPR rules.
2. The second screen with a selection of the game's gender
3. You choose a character (avatar). Both Emma and Adam will be on the screen and the player is able to choose between them.
4. In the first screen, the character has to drink, and the button for the drink will flash. Then a second room (toilet) will open where the player can send the character to the toilet. The character will need to take a nap and then go to the toilet. These are the most typical symptoms of diabetes, which are sometimes seen in children with diabetes, so the situation is unavoidable. Then the character is not feeling well and is taken away by the physician.
5. The nurse explains that Emma/Adam has been diagnosed with type 1 diabetes. For some reason, it is not their fault. The game explains that taking care of the character is necessary now.
6. Then the character appears in a home where there are 4 rooms (kitchen, bathroom, living room, children's room). The virtual physician appears, introduces herself and gradually explains the game and the actual situation. It's just before 5:30 pm, the character is in level 1,

has a small amount of money. MD says that the female character (the character will sometimes be Emma or Adam, depending on the player's choice) is hungry, but needs to wash her hands, measure glycaemia, and administrate the insulin before proceeding.
7. The handwash button is only accessible, and it is flashing.
8. The player completes the hand washing with soap and a towel. Followed by a reward for this handwashing action.
9. The button for measuring glycemia is flashing
10. Complete the glucose measurement step by step, indicated by arrows. The physician explained what the device is and how to use it. The reward for the treatment.
11. The button with the insulin will unlock and flash.
12. Injection of short-term insulin. The physician explains and recommends a specific value of insulin. It will not be possible to take any dosage than the physician recommends. MD will praise the player for the insulin application. It won't be possible to take the injection again, the physician explained.
13. Cutlery icon is flashing
14. The physician recommends a particular food that has already been bought. The food will is transferred by the player to the tray The physician points out the list of foods and their BUs. MD will praise the player for the food selection.
15. The character suddenly wants to go to the toilet again. The physician will not explain it further the WC room functionality.
16. Use the toilet. Reward.
17. The physician recommended sport.
18. Practising sport for half an hour. The physician explains that the exercise can be stopped and the stop button will flash.
19. The physician recommends a shower.
20. Use the shower. Reward for the shower.
21. The physician explains that the character is fine now and must wait until the second dinner. She recommends and explains the time rewind. (The rewind itself stops at the time of the second dinner.)
22. The character will be hungry. The second diner. The physician explained that the insulin is administrated after the second dinner.
23. Food intake - vegetables.
24. The physician reminds to wash hands.
25. BG measurement
26. Injection of a long-lasting insulin, because the night is getting closer - explained. The individual steps will flash (handwashing, BG measurement, insulin administration). For all the previous steps, the player receives award.
27. The MD says that the character needs to sleep, but he has to brush his teeth before doing so.
28. Brush your teeth. Reward.

29. Bedroom scene. Sleeping.
30. The physician recommended a time rewind
31. Once the night is over, the character is happy and advances to level 2. This is the end of the tutorial.

Table S17. Average time spent in the section during the first week of feasibility testing, in total 7 participants tested the game.

**Section**	**Description**	**Avg time per user (s)**
1-5	character selection and intro	167,01
6-8	MD introduction and hands wahs up	127,71
9-10	BG measurement	99,67
11-12	short-term insulin administration	132,53
13-14	food intake	61,43
15-16	wc	23,01
17-18	sport	58,4
19-21	shower nad time rewind	88,2
22-23	food intake	35,46
24	wash hand	43,91
25	BG measurement	57,43
26	long-term insulin administration	73,44
27-28	brushing teeth	29,21
29-31	sleeping and time rewind	51,79
29-11	tutorial	1049,19

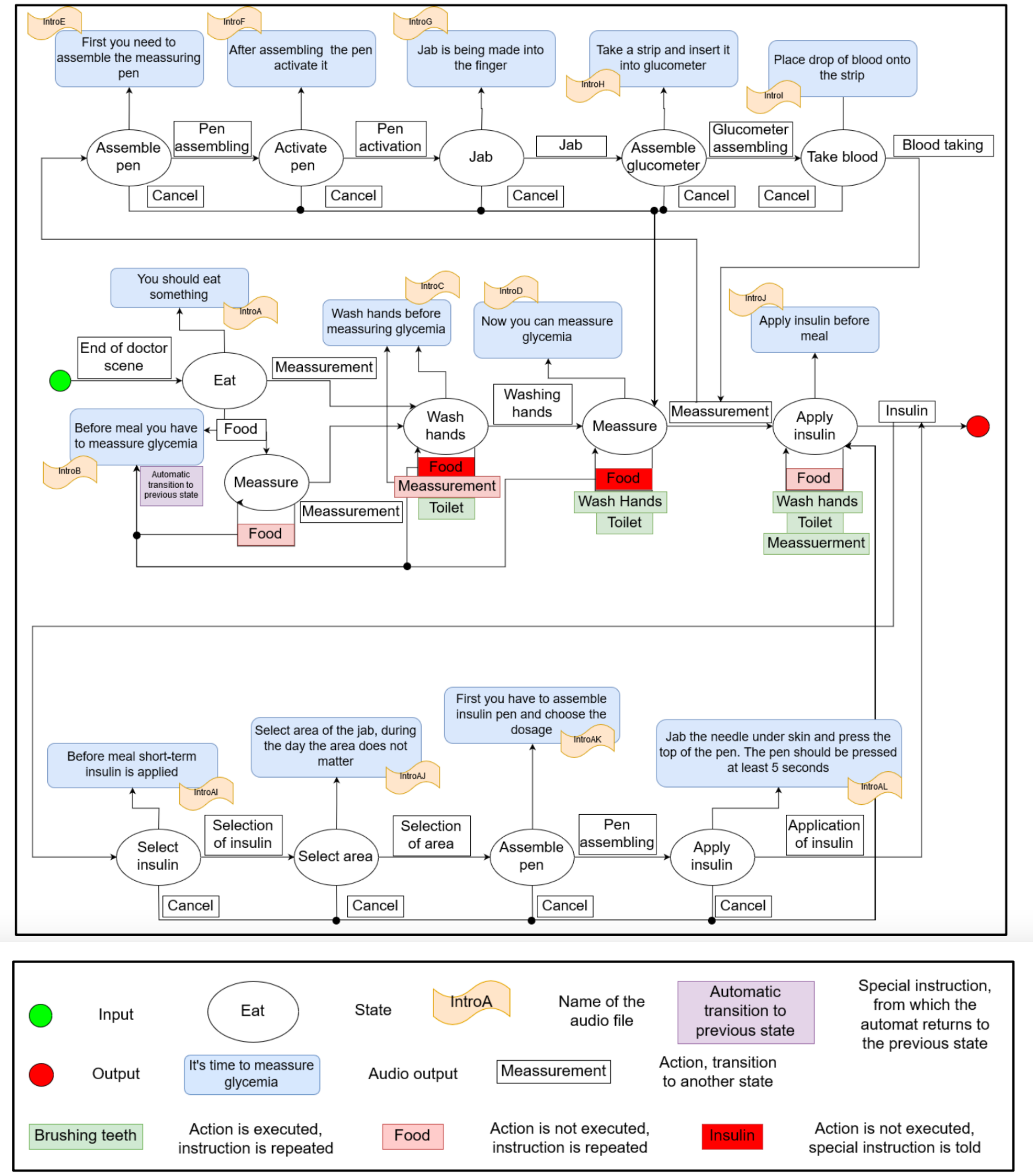

Figure S9. State automat for the tutorial

A8. Survey of serious T1D games

A8.1 Survey of serious T1D games

The results of the literature search, listed in section A8.2, are compiled in Table S18.

Table S18. Serious game on diabetes mellitus comparison

Author	Target group	Name	Methods	Education	Carb Intake	BG meas.	Insul. intake	Sports	Adv. concept	Validation	Real world use	Platform
Novak	4-12	MyDiabetic	participatory design, usability evaluation	x	x	x	x	x	x	Pilot study, 12 children	x	Android/iOS
Ebrahimour et al [15]	3-12	Koodad-e-Tavana	participatory design, pilot testing	x			x					PC
Calle-Bustos et al [16]	5 - 14		pre and post questionnaire	x	x					70 children		
Glasemann [17]	>10		participatory design, interview with dieticiens, user test	x	x							
Brox et al [18]	8-12		User centric design, focus-testing	x					x			
Liberman [19]	8-16	Packy Marlon	review of clinical findings	x	x	x	x			[illegible] in diabetes related urgemt care, 6	not now	Nintendo
Peters [20]	8-10		usability evaluation	x								
Ronningen [21]	>9	Diaquarium	participatory design, usability evaluation	x	x							PC
Baghaei [22]	9-13	Mario Brothers	pre-post pilot study							Pilot study 12 children		Android
Pesare [23]	8-12		pre-post pilot study	x	x					Pilot study 20 children		
Lauritzen [24]	7-12		games									
Bomark [25]	8-12		demonstration proposal	x								
D'Aprile [26]	11-17	Tako Dojo	usability test, questionnairse	x						Observation study, 124 participants	not now	Android/iOS
Moosa [27]	8-11	Qure and Qure		x								
Sparapani [28]	7-12		methodological study	x								
Boulos [29]	6-10	Monster Manor	review	x		x					not now	Android/iOS
Jimenez [30]		with Lenny	review	x		x						Android/iOS
Ling [31]	7-12*	Diabetic Dog	methodological study	x	x		x				not now	Web app
Ullman [32]	4-9	Jerry the Bear		x	x	x	x		x		x	Android/iOS

A9. References

[1] Sweetser P, Wyeth P. GameFlow: a model for evaluating player enjoyment in games. Comput Entertain 2005 Jul;3(3):3–3. doi: 10.1145/1077246.1077253

[2] Hunicke R, LeBlanc M, Zubek R. MDA: A Formal Approach to Game Design and Game Research. I. Proc AAAI Workshop Chall Game AI.

[3] Ganesh A, Ndulue C, Orji R. Tailoring a Persuasive Game to Promote Secure Smartphone Behaviour. Proc 2023 CHI Conf Hum Factors Comput Syst Hamburg Germany: ACM; 2023. p. 1–18. doi: 10.1145/3544548.3581038

[4] Thompson D. Designing serious video games for health behavior change: current status and future directions. J Diabetes Sci Technol 2012 Jul 1;6(4):807–811. PMID:22920806

[5] Yari A, Hassani L, Ziapour A, Toghroli R, Mohammadkhah F, Yazdi F. Prediction of self-care behaviors among diabetic patients based on social cognitive theory. J Public Health Res 2023 Jan;12(1):227990362211503. doi: 10.1177/22799036221150330

[6] Thompson D, Baranowski T, Buday R. Conceptual model for the design of a serious video game promoting self-management among youth with type 1 diabetes. J Diabetes Sci Technol 2010 May 1;4(3):744–749. PMID:20513343

[7] Novak D, Tulu B, Brendryen H. Handbook of Research on Holistic Perspectives in Gamification for Clinical Practice. Hershey: IGI Global; 2015.

[8] Brorsson AL, Leksell J, Viklund G, Lindholm Olinder A. A multicentre randomized controlled trial of an empowerment-inspired intervention for adolescents starting continuous subcutaneous insulin infusion--a study protocol. BMC Pediatr 2013 Dec 20;13:212. PMID:24354899

[9] Almeida AC, Leandro ME, Pereira MG. Adherence and Glycemic Control in Adolescents with Type 1 Diabetes: The Moderating Role of Age, Gender, and Family Support. J Clin Psychol Med Settings 2020 Jun;27(2):247–255. doi: 10.1007/s10880-019-09662-y

[10] Lehmann ED. Why people download the freeware AIDA v4.3a diabetes software program: a proof-of-concept semi-automated analysis. Diabetes Technol Ther 2003;5(3):477–490. PMID:12828835

[11] KUDVA, Yogish C, Andrew J AHMANN, Richard M BERGENSTAL, James R GAVIN, Davida F KRUGER, L Kurt MIDYETT, Eden MILLER a Dennis R HARRIS. Approach to Using Trend Arrows in the FreeStyle Libre Flash Glucose Monitoring Systems in Adults. Journal of the Endocrine Society [online]. 2018, 2(12), 1320-1337

[12] ALEPPO, Grazia, Lori M LAFFEL, Andrew J AHMANN, Irl B HIRSCH, Davida F KRUGER, Anne PETERS, Ruth S WEINSTOCK a Dennis R HARRIS. A Practical Approach to Using Trend Arrows on the Dexcom G5 CGM System for the Management of Adults With Diabetes. Journal of the Endocrine Society [online]. 2017, 1(12), 1445-1460

[13] P. Fabietti, V Canonico, M. Federici, E. Sarti a M. Benedetti, Model Based Study on Monitoring Ketone Bodies to Improve Safety in Intensive Insulin Therapy, The International journal of artificial organs, 29, s. 596–601, 2006
[14] C. R. Barnett a Y. A. Barnett, „KETONE BODIES“, in Encyclopedia of Food Sciences and Nutrition, Maryland, USA: Academic Press, 2003
[15] Ebrahimpour F, Najafi M, Sadeghi N. The design and development of a computer game on insulin injection. Electron Physician 2014;6(2):845–855. PMID:25763157
[16] Calle-Bustos A-M, Juan M-C, García-García I, Abad F. An augmented reality game to support therapeutic education for children with diabetes. PloS One 2017;12(9):e0184645. PMID:28957355
[17] Glasemann M, Kanstrup AM, Ryberg T. Making chocolate-covered broccoli: designing a mobile learning game about food for young people with diabetes. Proc 8th ACM Conf Des Interact Syst Aarhus Denmark: ACM; 2010. p. 262–271. doi: 10.1145/1858171.1858219
[18] Brox E, Hirche J, Evertsen G, Yliräisänen-Seppänen P, Bomark P. User centric social diabetes game design for children. Proceeding 16th Int Acad MindTrek Conf Tampere Finland: ACM; 2012. p. 291–293. doi: 10.1145/2393132.2393196
[19] Lieberman DA. Management of chronic pediatric diseases with interactive health games: theory and research findings. J Ambulatory Care Manage 2001 Jan;24(1):26–38. PMID:11189794
[20] Peters R, Oleari E, Sardu F, Neerincx MA. Usability of the PAL Objectives Dashboard for Children's Diabetes Self-Management Education. Proc 2019 5th Int Conf E-Soc E-Learn E-Technol - ICSLT 2019 Vienna, Austria: ACM Press; 2019. p. 22–28. doi: 10.1145/3312714.3312736
[21] Rønningen IC, Årsand E, Hartvigsen G. Exploring In-Game Reward Mechanisms in Diaquarium – A Serious Game for Children with Type 1 Diabetes. In: Rojas I, Ortuño F, editors. Bioinforma Biomed Eng Cham: Springer International Publishing; 2018. p. 443–455. doi: 10.1007/978-3-319-78759-6_40
[22] Baghaei N, Nandigam D, Casey J, Direito A, Maddison R. Diabetic Mario: Designing and Evaluating Mobile Games for Diabetes Education. Games Health J 2016 Aug;5(4):270–278. PMID:27304882
[23] Pesare E, Roselli T, Rossano V. Intelligent Agent and Virtual Game to support education in e-health. 2015. p. 204–211. doi: 10.18293/DMS2015-035
[24] Lauritzen J. SOCIAL MEDIA AND GAMES AS SELF-MANAGEMENT TOOLS FOR CHILDREN AND ADOLESCENTS WITH TYPE 1 DIABETES MELLITUS: Proc Int Conf Health Inform Vilamoura, Algarve, Portugal: SciTePress - Science and and Technology Publications; 2012. p. 459–466. doi: 10.5220/0003874104590466
[25] Bomark P, Evertsen G, Brox E, Hirche J, Yliräisänen-Seppänen P. A prototype social learning platform for children with diabetes type 1. Proceeding 16th Int Acad MindTrek Conf Tampere Finland: ACM; 2012. p. 211–213. doi: 10.1145/2393132.2393174

[26] D'Aprile G, Ligorio MB, Ulloa Severino A. How Serious Games for Health Enhance Empowerment-Related Patient Education: The Impact of Gender. Technol Knowl Learn 2019 Sep;24(3):325–340. doi: 10.1007/s10758-017-9344-x
[27] Moosa AM, Al-Maadeed N, AlJa'am JM. A Simple Health-Based Game for Children. 2018 Int Conf Comput Appl ICCA Beirut: IEEE; 2018. p. 309–312. doi: 10.1109/COMAPP.2018.8460213
[28] Sparapani VDC, Fels S, Kamal N, Nascimento LC. Conceptual framework for designing video games for children with type 1 diabetes. Rev Lat Am Enfermagem 2019;27:e3090. doi: 10.1590/1518-8345.2764.3090
[29] Kamel Boulos MN, Gammon S, Dixon MC, MacRury SM, Fergusson MJ, Miranda Rodrigues F, Mourinho Baptista T, Yang SP. Digital Games for Type 1 and Type 2 Diabetes: Underpinning Theory With Three Illustrative Examples. JMIR Serious Games 2015 Mar 18;3(1):e3. doi: 10.2196/games.3930
[30] Jimenez G, Lum E, Car J. Examining Diabetes Management Apps Recommended From a Google Search: Content Analysis. JMIR MHealth UHealth 2019 Jan 16;7(1):e11848. PMID:30303485
[31] Ling C, Seetharaman S, Mirza L. Roles of Serious Game in Diabetes Patient Education. Simul Gaming 2022 Oct;53(5):513–537. doi: 10.1177/10468781221120686
[32] Ullman D, Phillips E, Aladia S, Haas P, Fowler HS, Iqbal IS, Mi KL, Riches IW, Omori M, Malle BF. Evaluating Psychosocial Support Provided by an Augmented Reality Device for Children With Type 1 Diabetes. Proc Int Symp Hum Factors Ergon Health Care 2021 Jun;10(1):126–130. doi: 10.1177/2327857921101117
