# Supplementary material for: A Serious Game (MyDiabetic) to Support Children’s Education in Type 1 Diabetes Mellitus: Iterative Participatory Co-Design and Feasibility Study
Source: JMIR Serious Games. 2024 May 7;12:e49478. doi: 10.2196/49478 (PMC11109855; doi:10.2196/49478)
Supplement: Multimedia Appendix 3 [file games_v12i1e49478_app3.pdf]

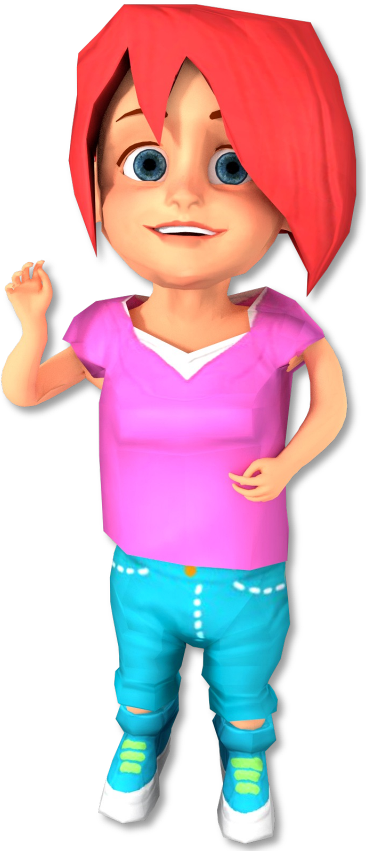

Educational game

MyDiabetic

For childer 5-12 years with T1D

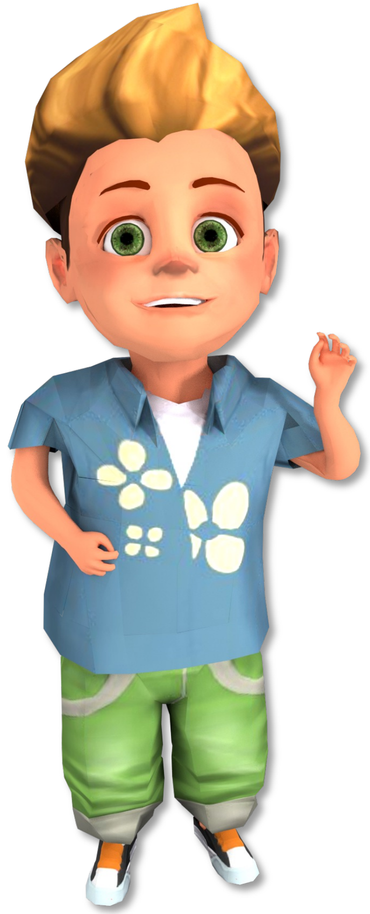

Daniel Novák
CTU, Prague

Content

Intro
Gamification
Some games
Goals
Concept
Features
What next
Thanks

Goals

- TO HELP CHILDREN UNDERSTAND THEIR DISEASE.
- CARBS COUNTING
- SHOW SYMTOMPS OF HYPO/HYPERGLYCEMIA AND SOLUTION
- INSULIN TREATMENT DEMONSTRATION
- MOTIVATION TO MOVEMENT
- NOT JUST EDUCATION BUT JOY
- ALSO FOR FRIENDS !!!

Content

Intro

Gamification

Some games

Goals

Concept

Features

What next

Thanks

Some games

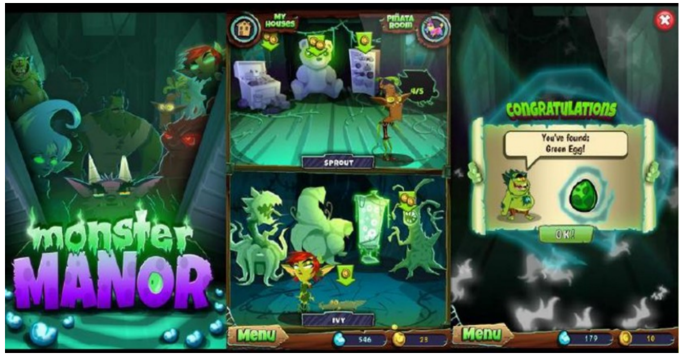

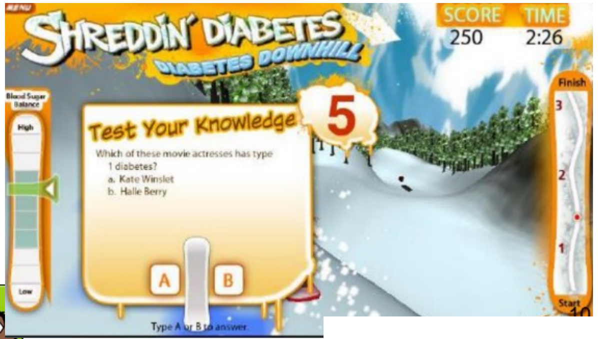

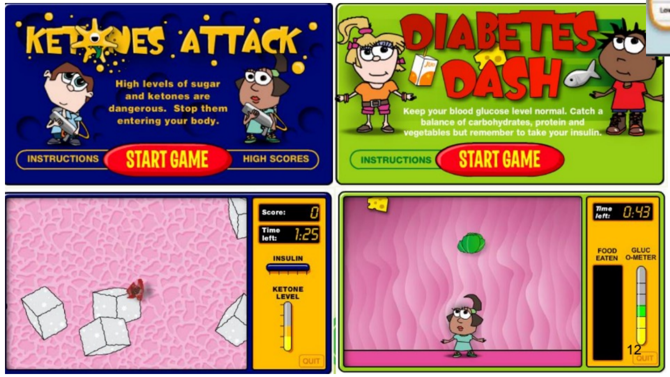

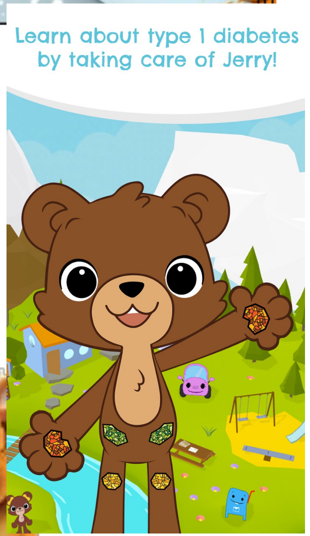

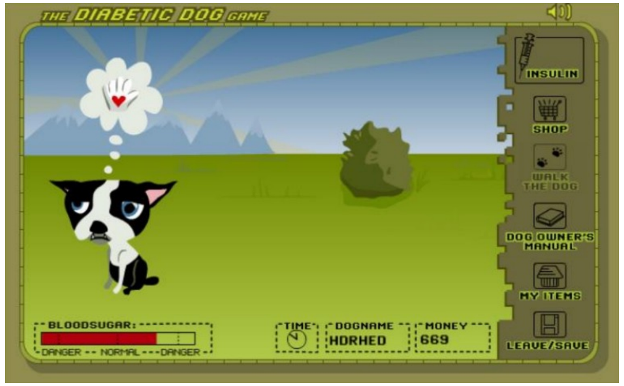

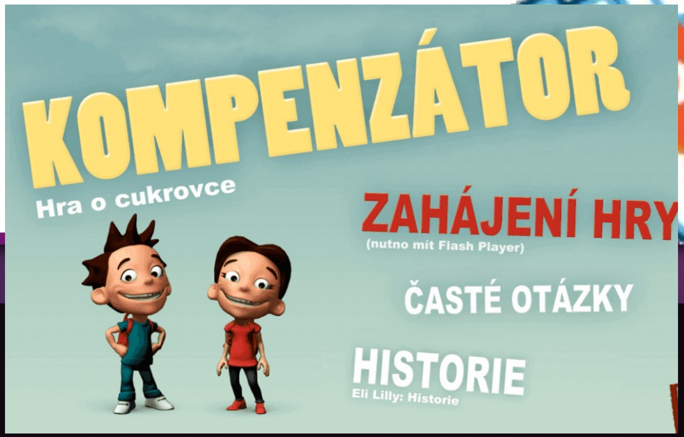

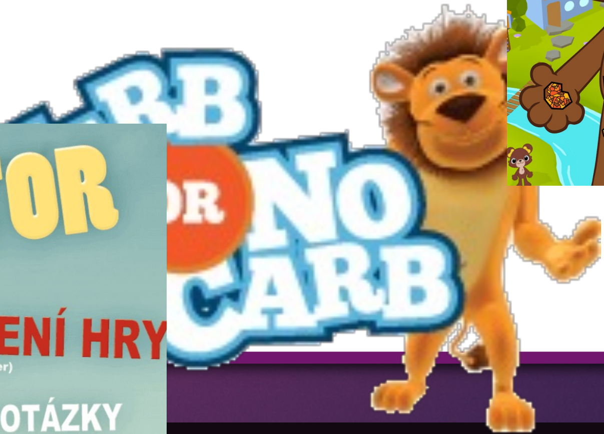

Content

Intro
Gamification
Some games
Goals
Concept
Features
What next
Thanks

Game concept

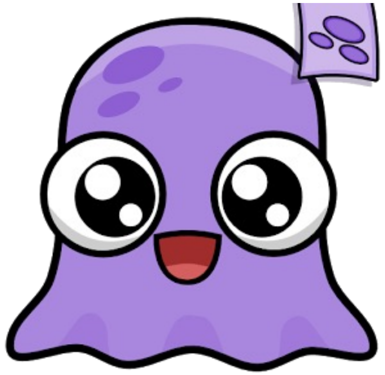

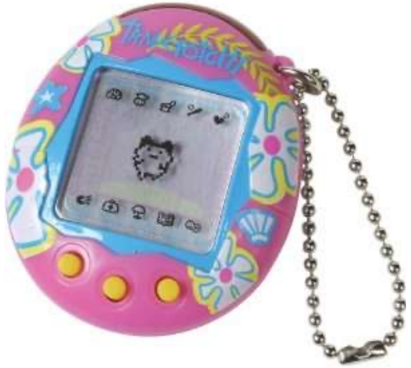

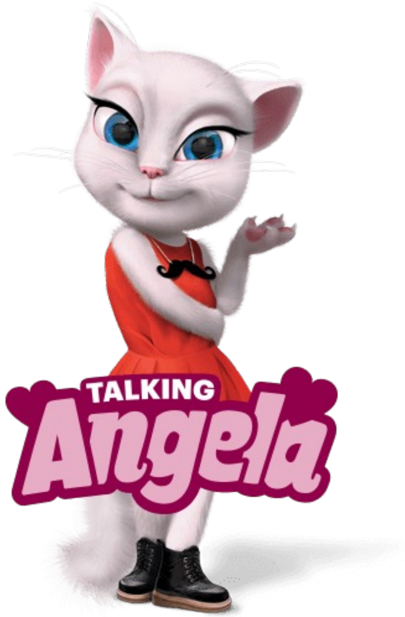

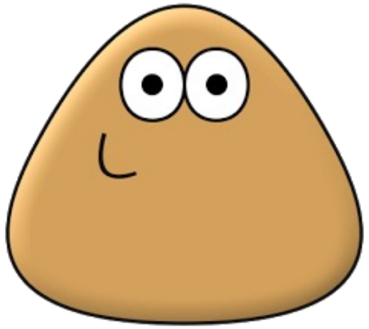

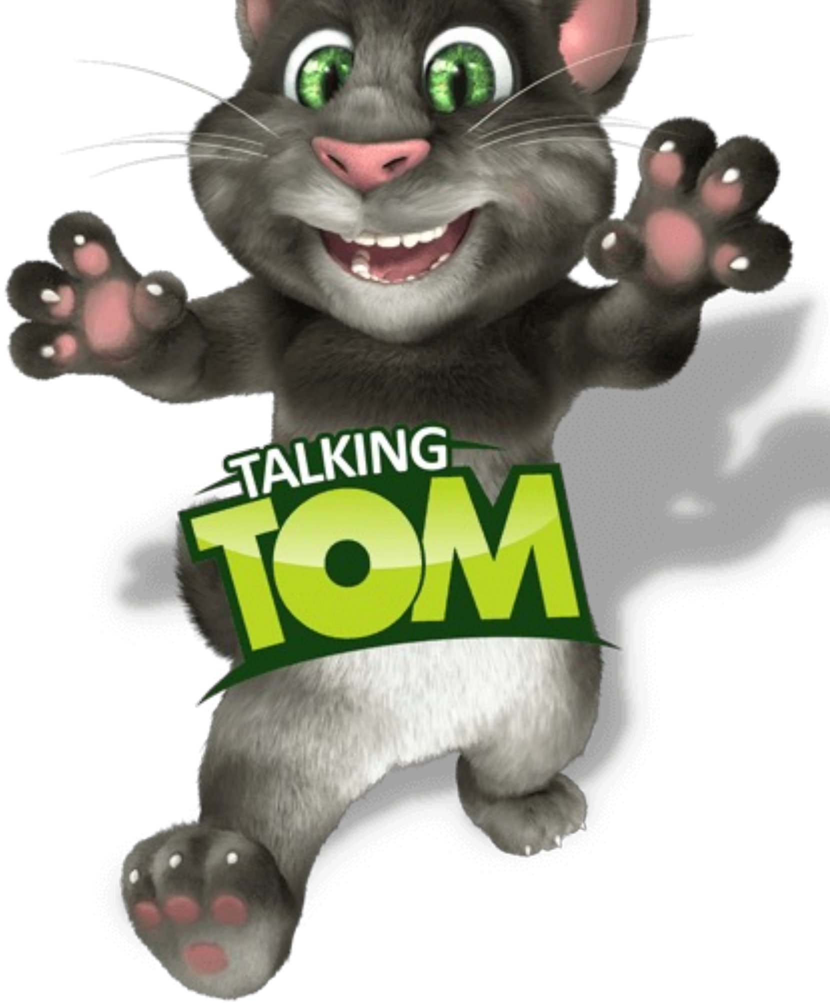

Content

Intro
Gamification
Some games
Goals
Concept
Features
What next
Thanks

User-Centered Design

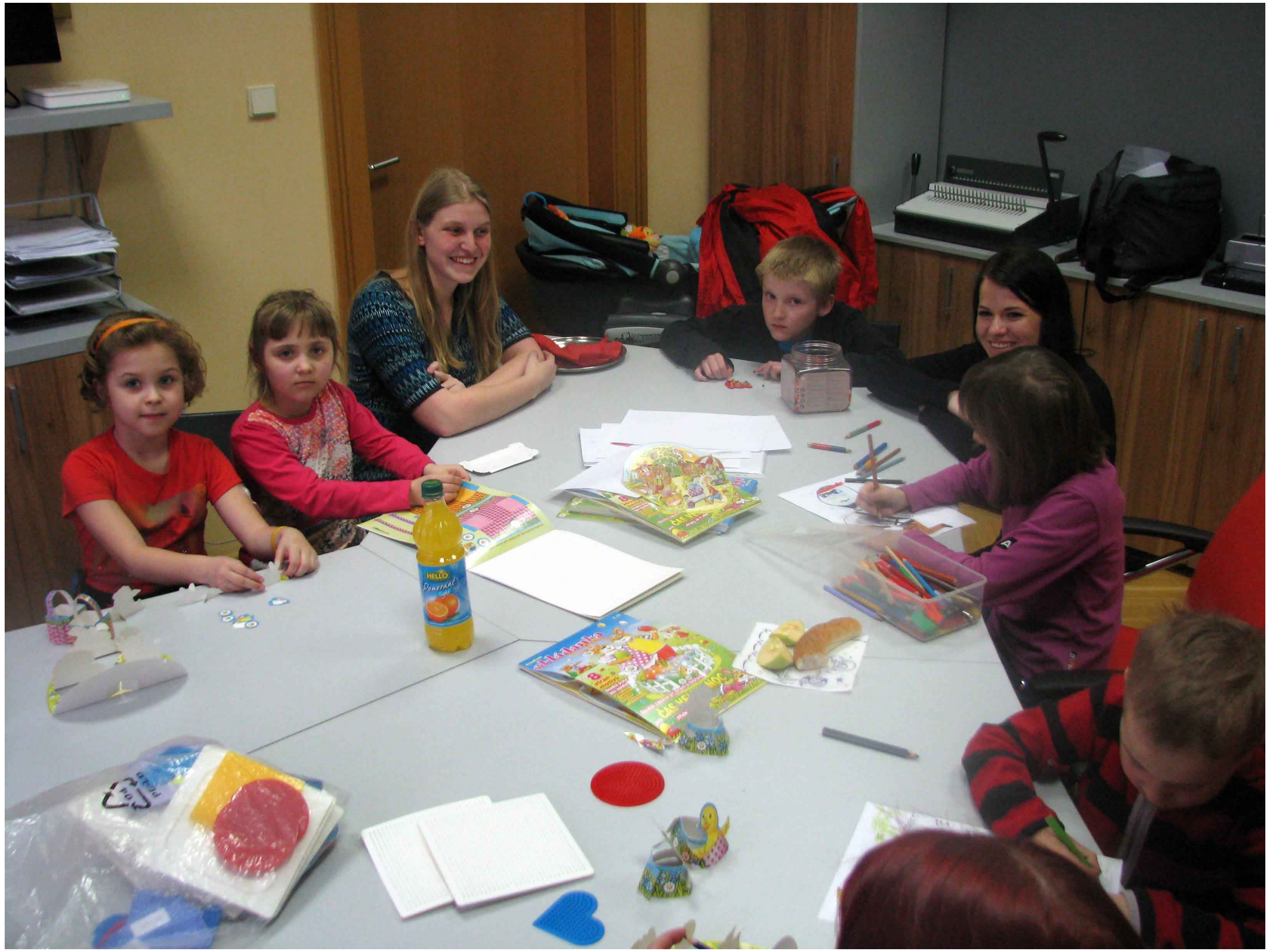

Content

Intro

Gamification

Some games

Goals

Concept

Features

What next

Thanks

Participatory Co-Design

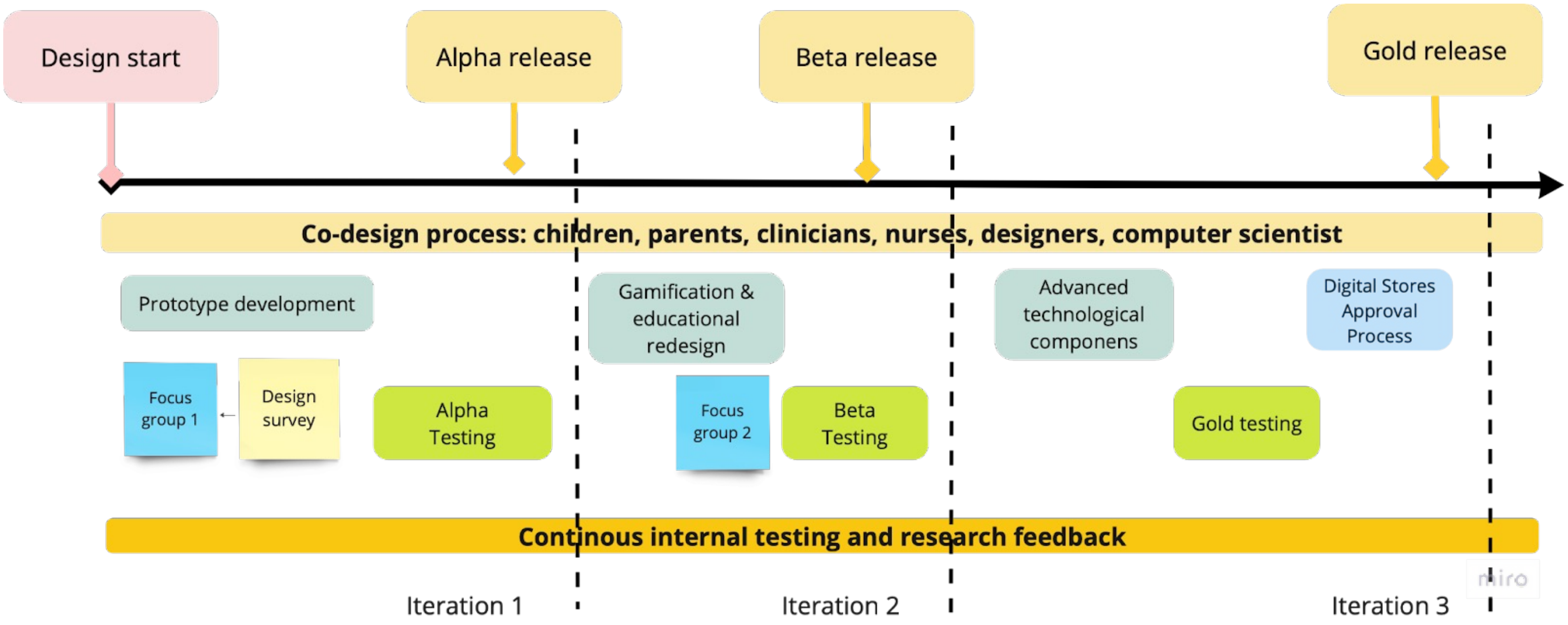

Content

Intro
Gamification
Some games
Goals
Concept
Features
What next
Thanks

Participatory Co-Design

Iteration	Activity	Users (m/f)	Age range	Stages of development	Features introduced
1	Focus group	4/0	11-14	mock-up	Basic gaming concept, design of control elements, taking-care avatar concept, carb counting, insulin-pen application, glycemia measurement, practising sports
	Design survey	15/12	7-13	mock-up	
	Alpha testing	8/4(*)	6-13	Alpha release	
2	Beta testing	2/4	5-15	Beta release	Education elements as virtual interactive library or simulations of blood glucose, minigames, story line and levels design
3	Gold testing	7/2	10-15	Gold (final) release	Advanced topics as CGM and insulin pump illustration, measurement ketoacidosis and application of glucagon
		2/5	8-16		
		4/4	7-15		

Main design

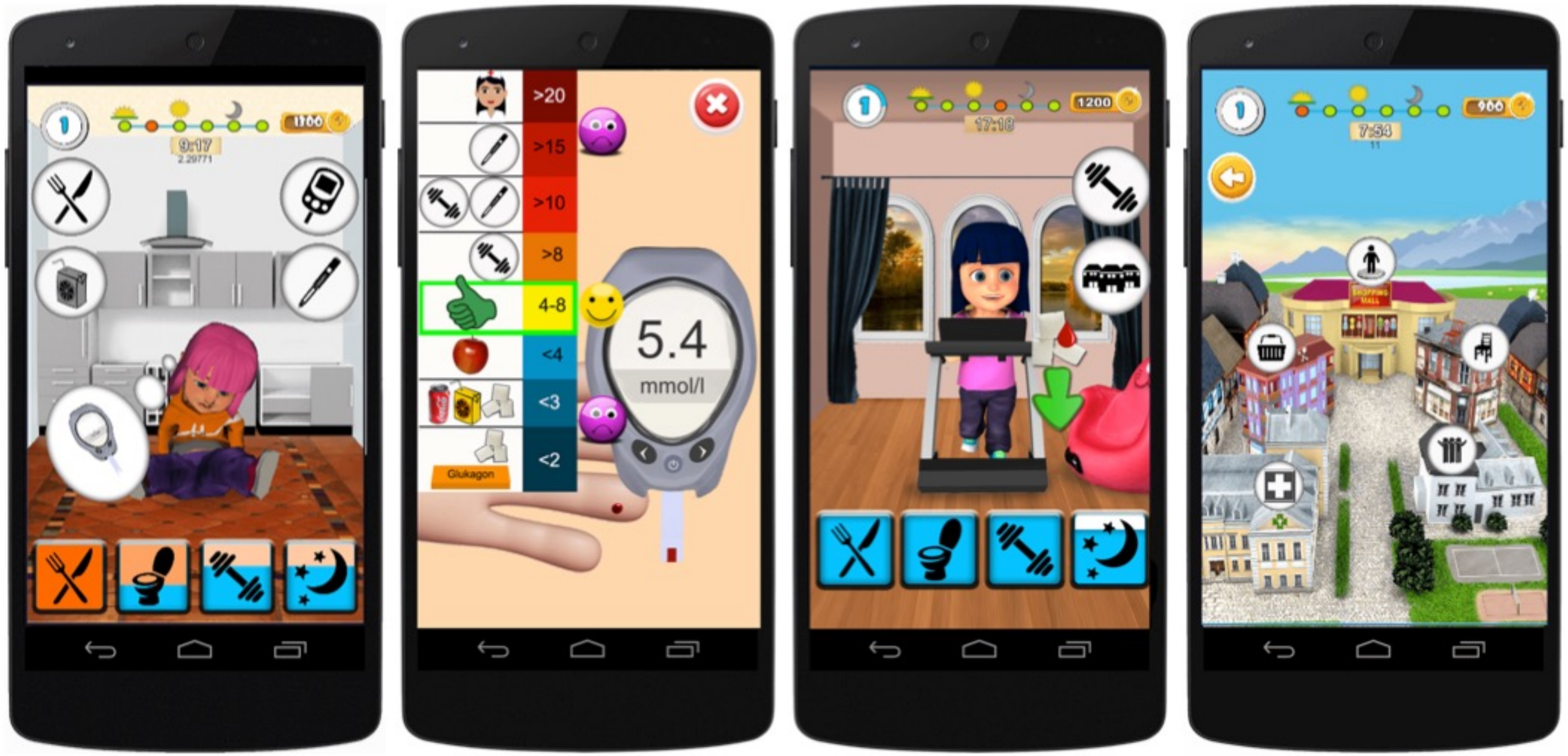

Content

Intro
Gamification
Some games
Goals
Concept
Features
What next
Thanks

First symptomps

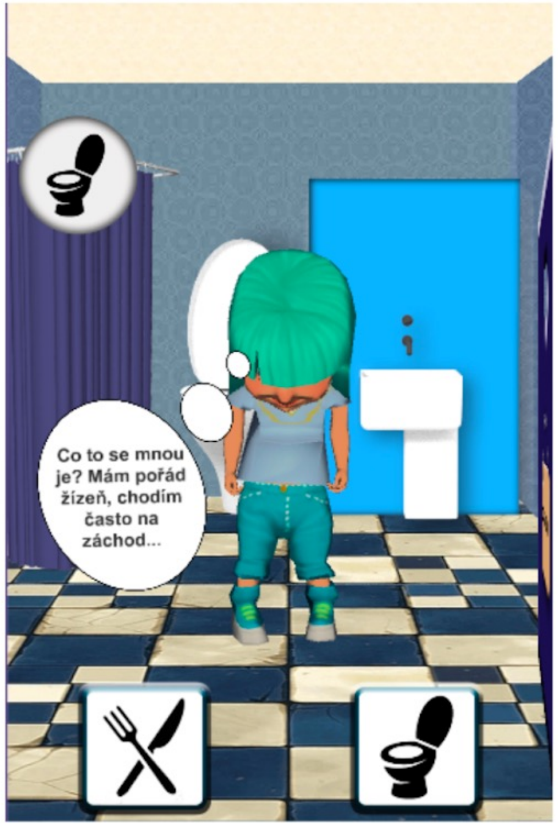

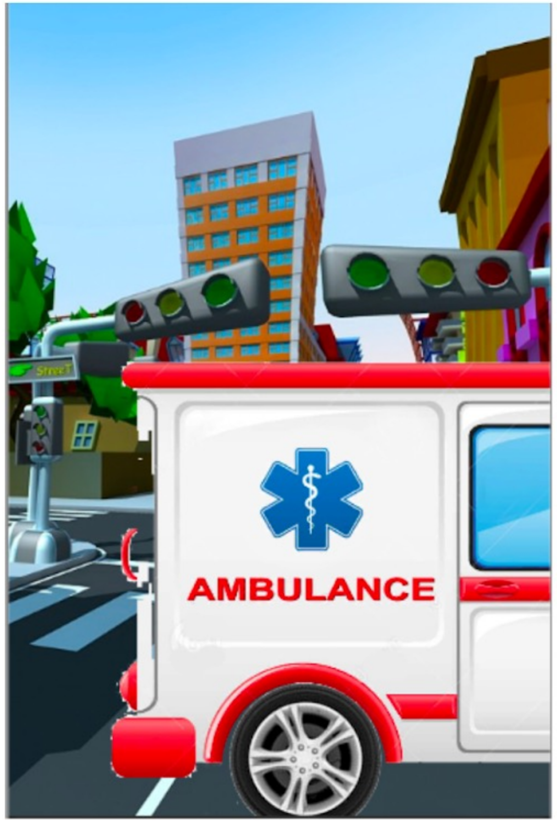

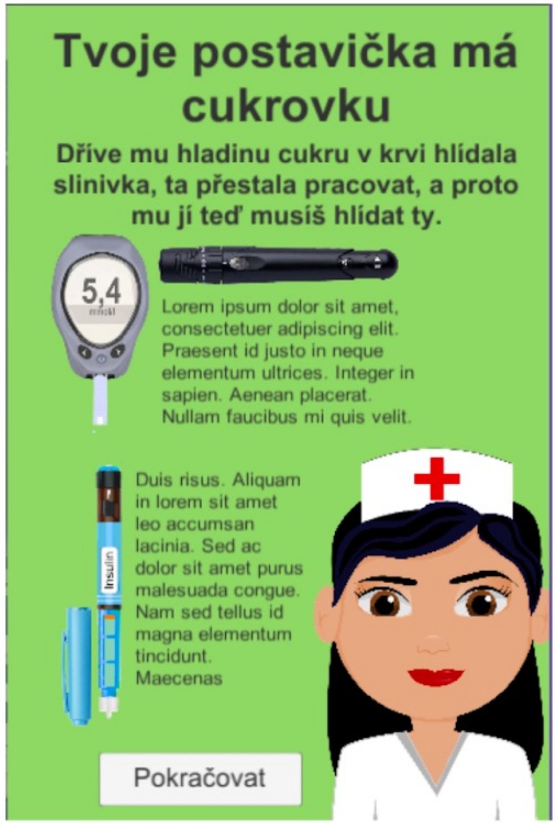

Content

Intro
Gamification
Some games
Goals
Concept
Features
What next
Thanks

Glycemia measurement

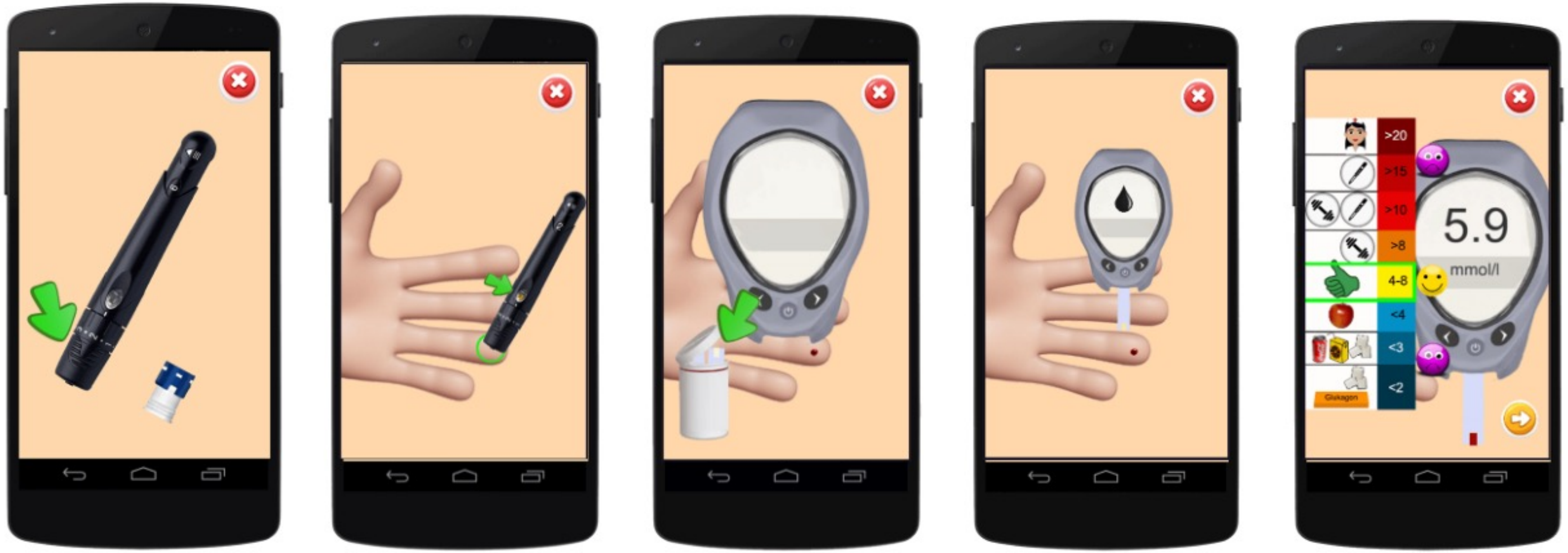

Content

Intro
Gamification
Some games
Goals
Concept
Features
What next
Thanks

Demonstration of insulin application

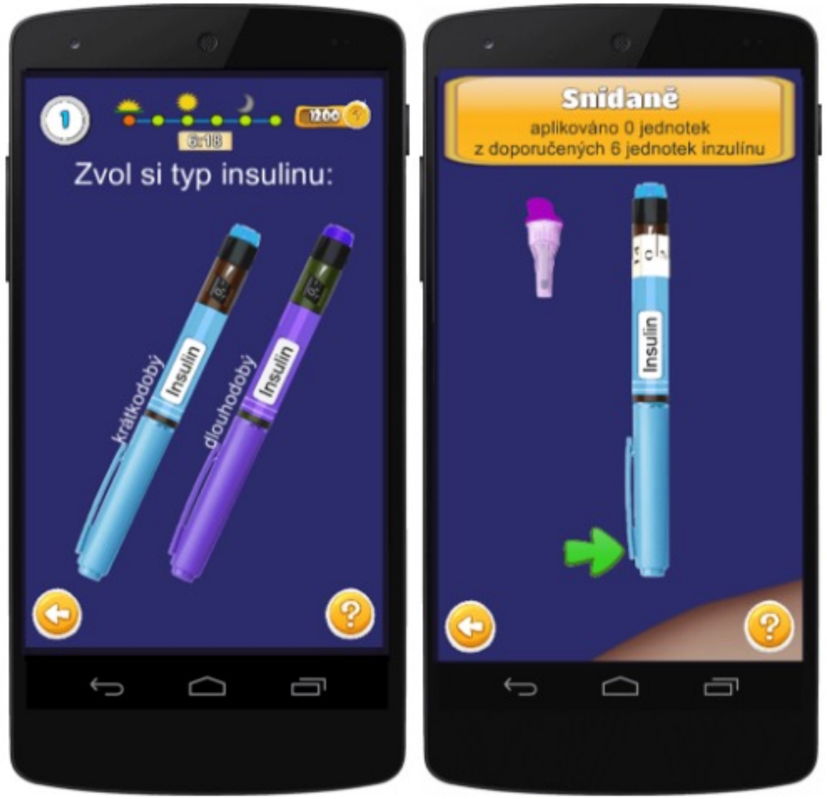

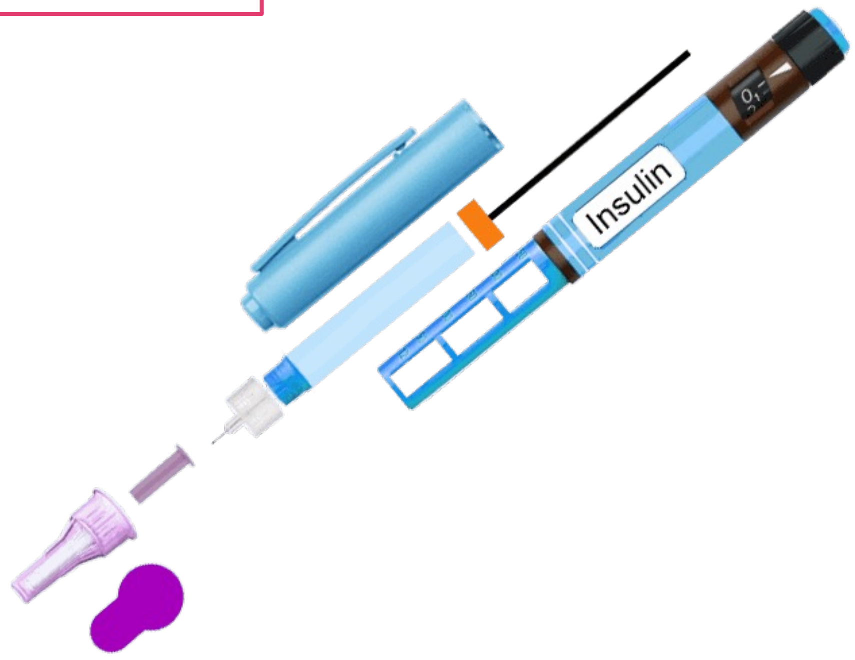

Content

Intro
Gamification
Some games
Goals
Concept
Features
What next
Thanks

Carbs counting

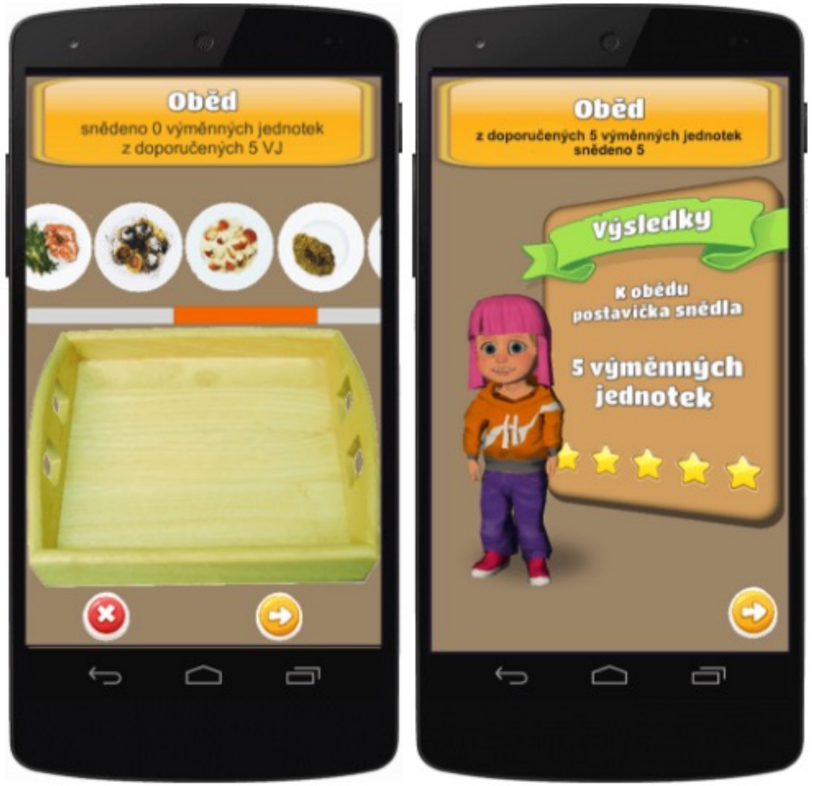

Content

Intro
Gamification
Some games
Goals
Concept
Features
What next
Thanks

Symptoms of hypo/hyper

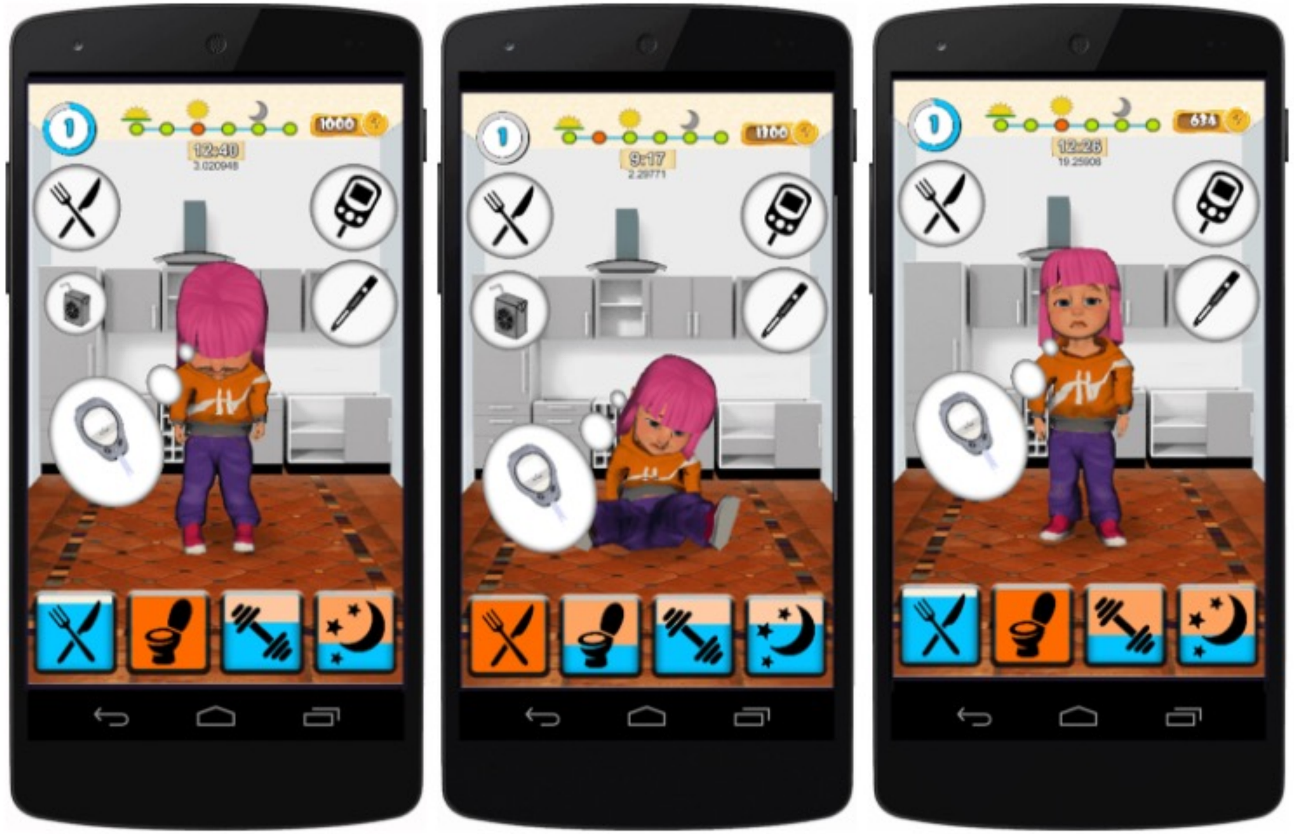

Content

Intro
Gamification
Some games
Goals
Concept
Features
What next
Thanks

Preschool children - audio guidance

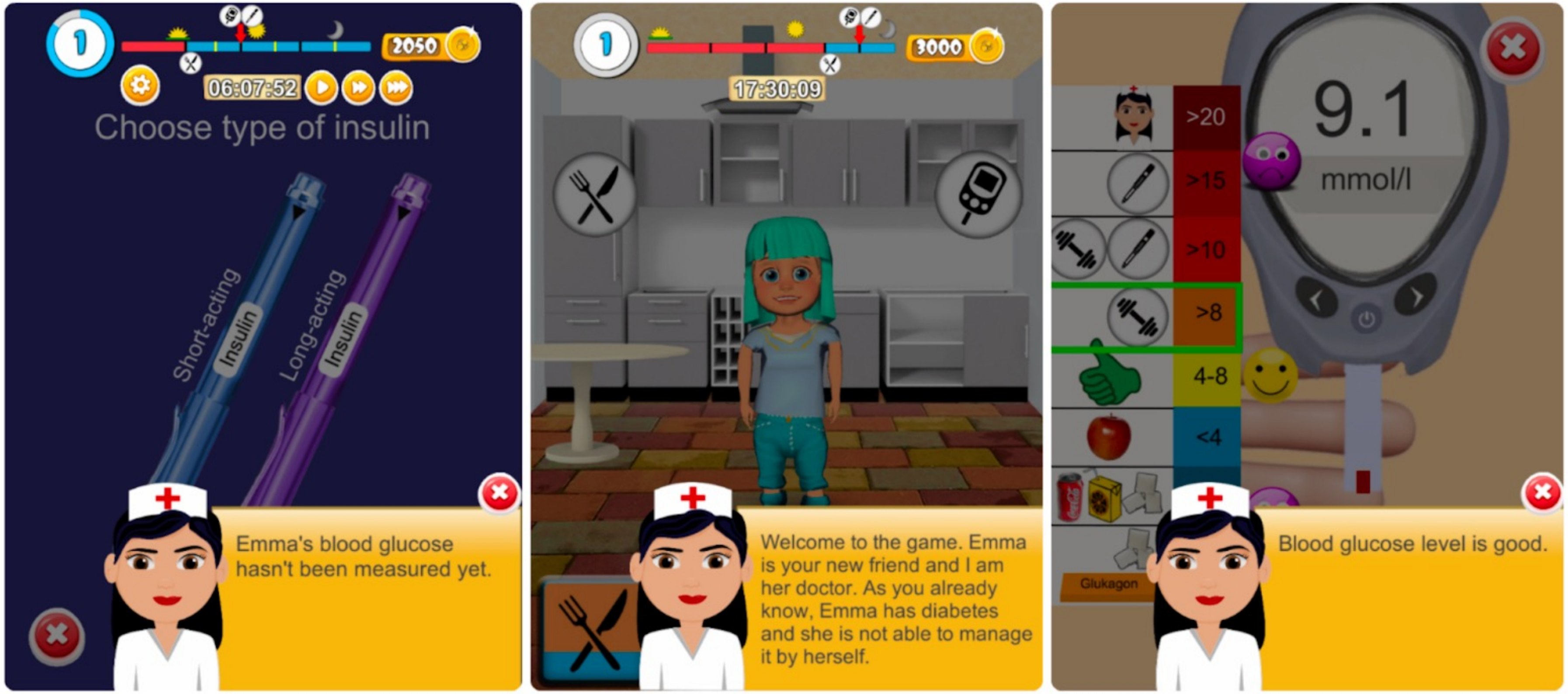

Content

Intro
Gamification
Some games
Goals
Concept
Features
What next
Thanks

Insulin pump: infusion set replacement

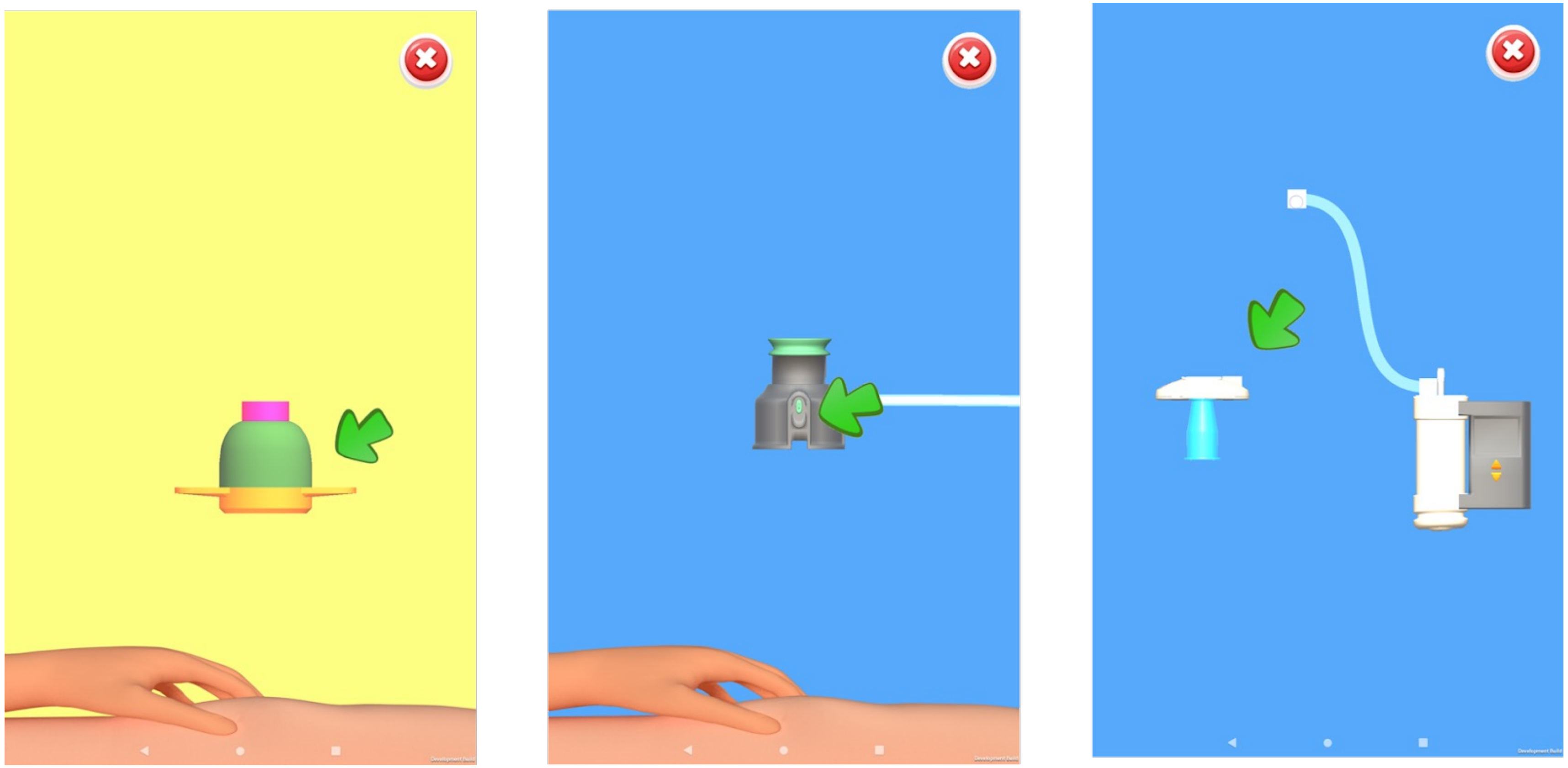

Content

Intro
Gamification
Some games
Goals
Concept
Features
What next
Thanks

CGM

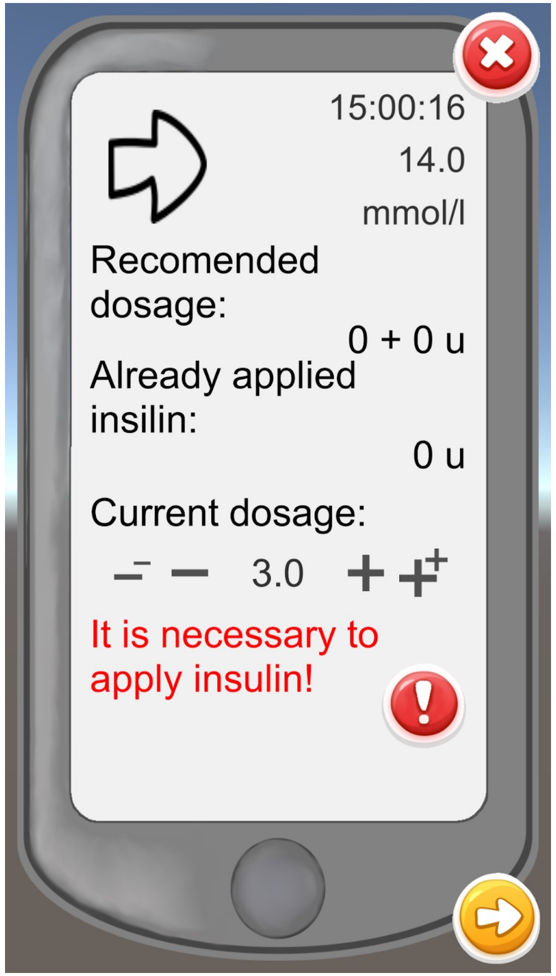

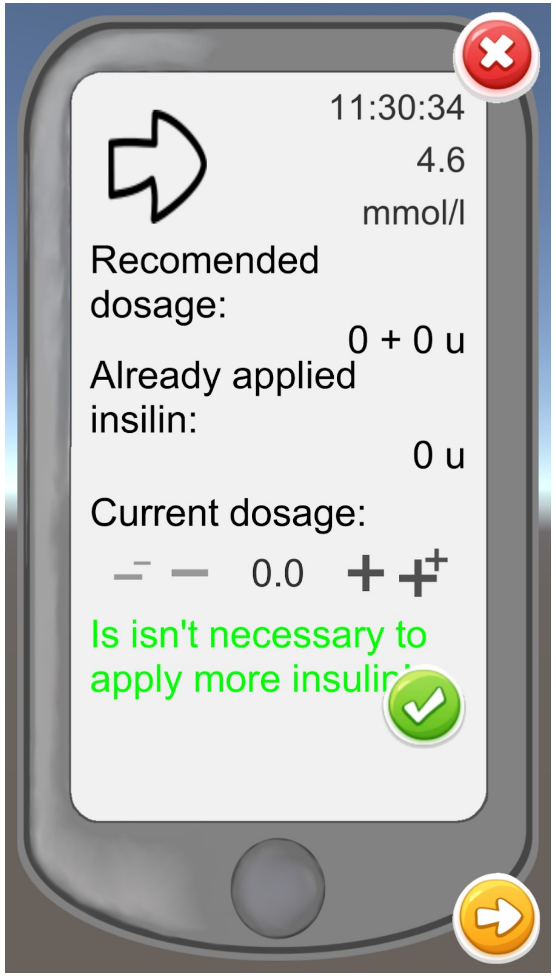

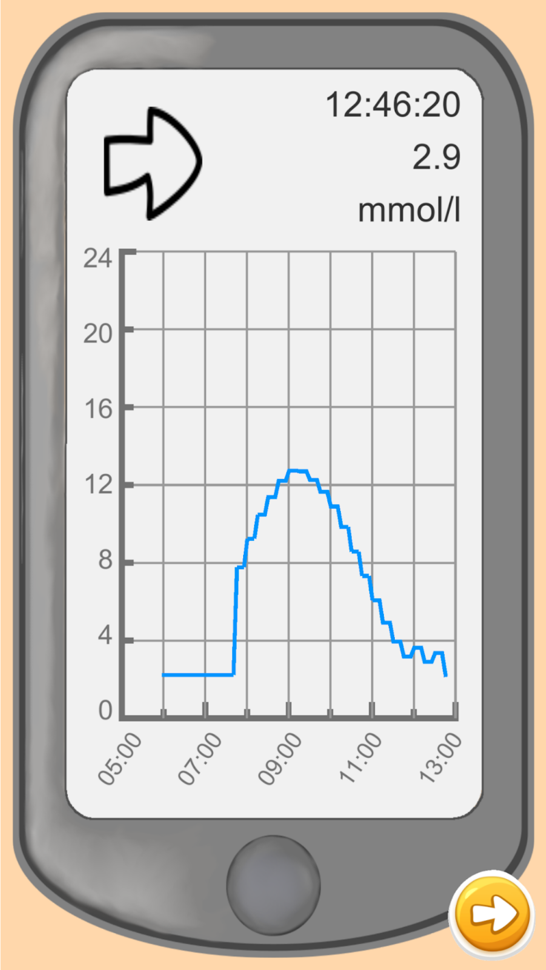

Content

Intro
Gamification
Some games
Goals
Concept
Features
What next
Thanks

Glucagon application

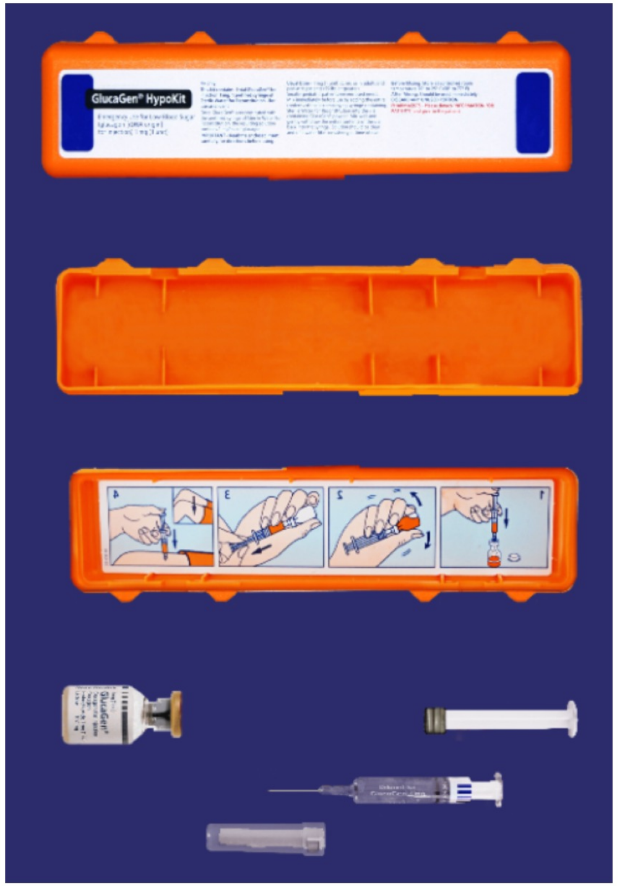

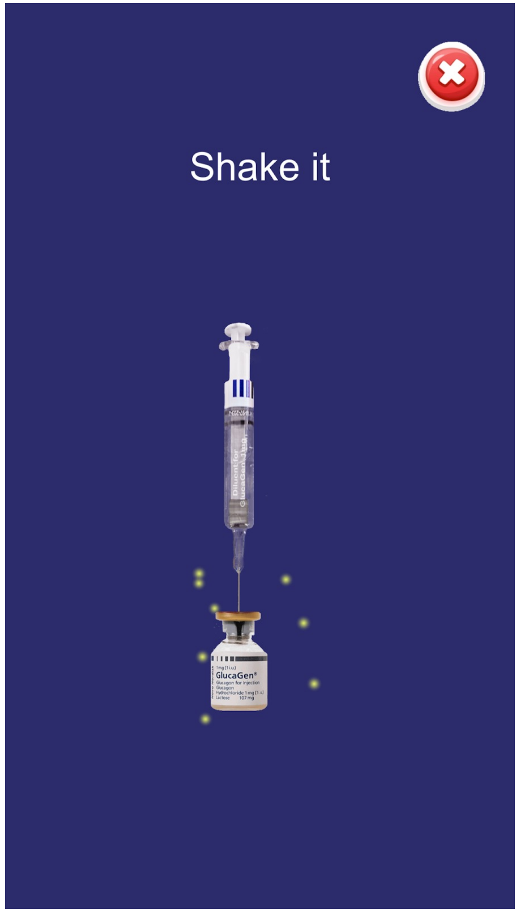

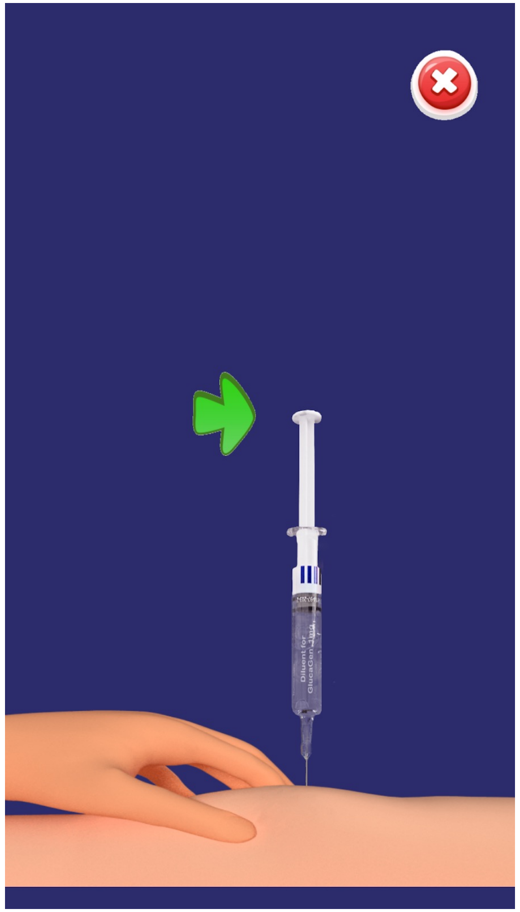

Content

Intro
Gamification
Some games
Goals
Concept
Features
What next
Thanks

Ketones urine test

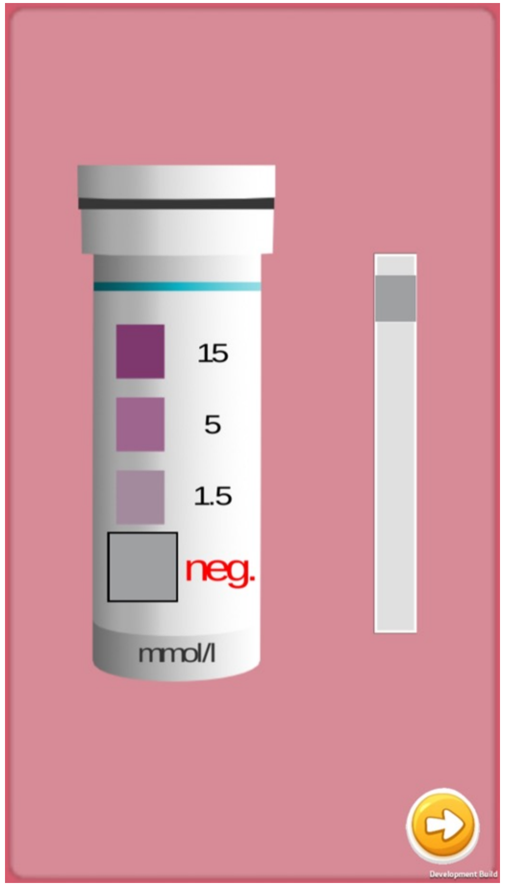

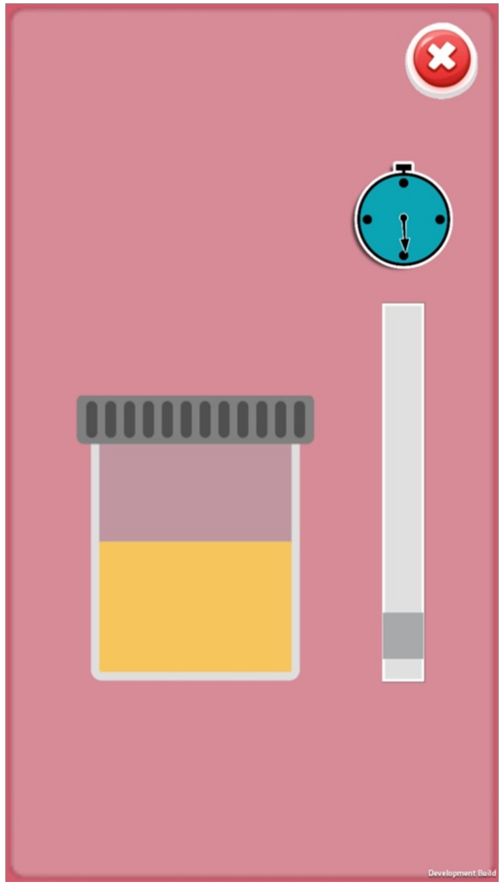

Content

Intro
Gamification
Some games
Goals
Concept
Features
What next
Thanks

Usability testing

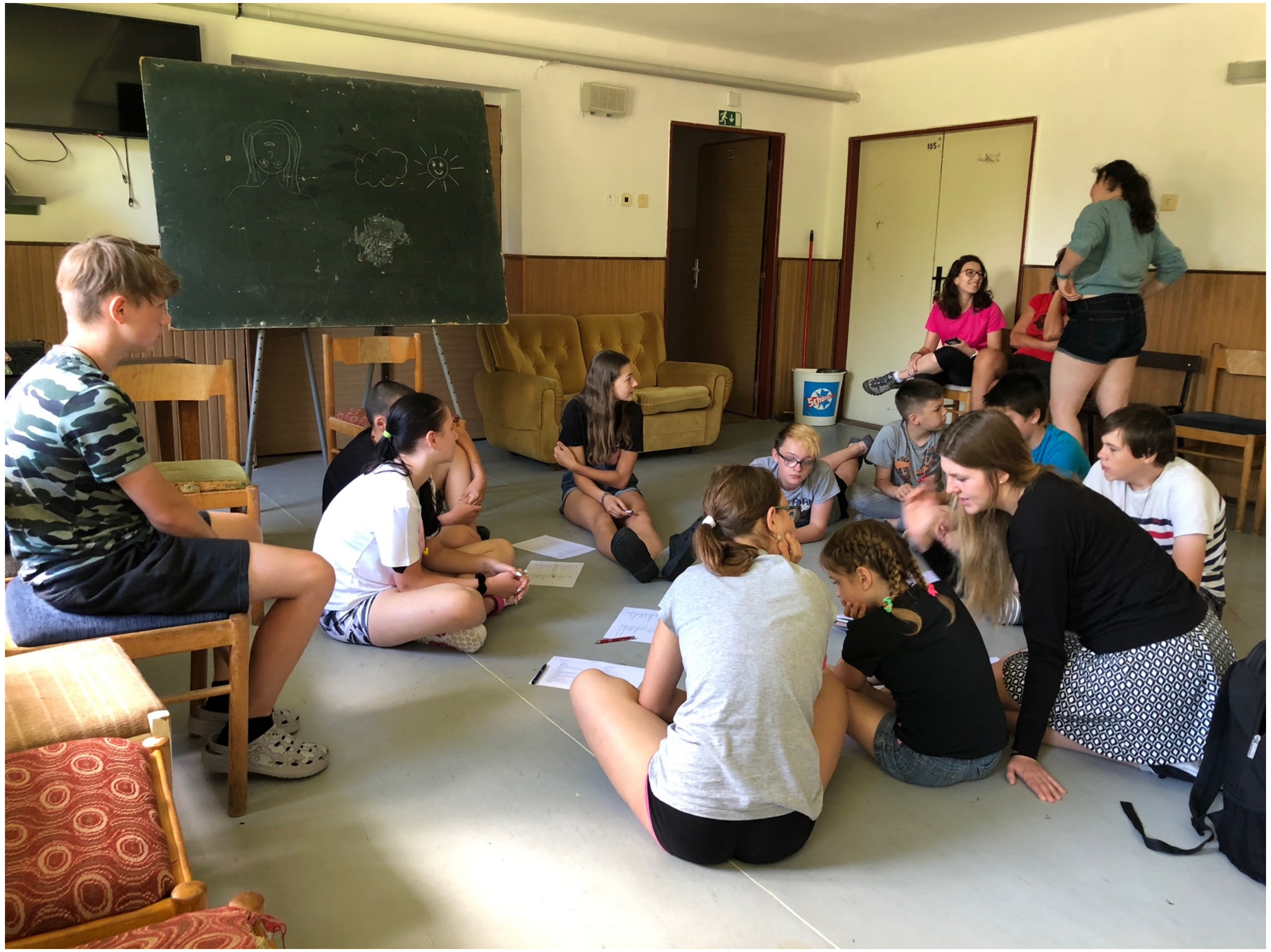

Content

Intro

Gamification

Some games

Goals

Concept

Features

What next

Thanks

Education but joy

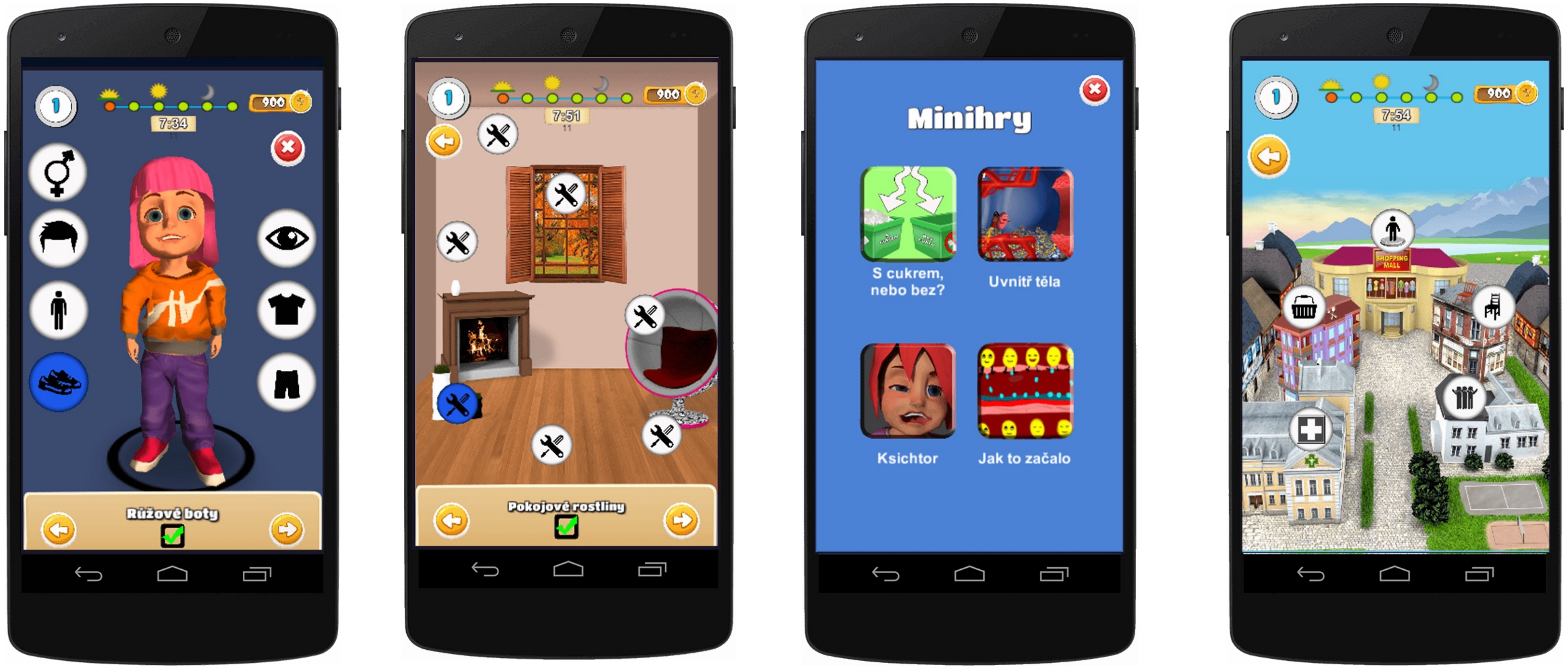

Content

Intro
Gamification
Some games
Goals
Concept
Features
What next
Thanks

Education but joy

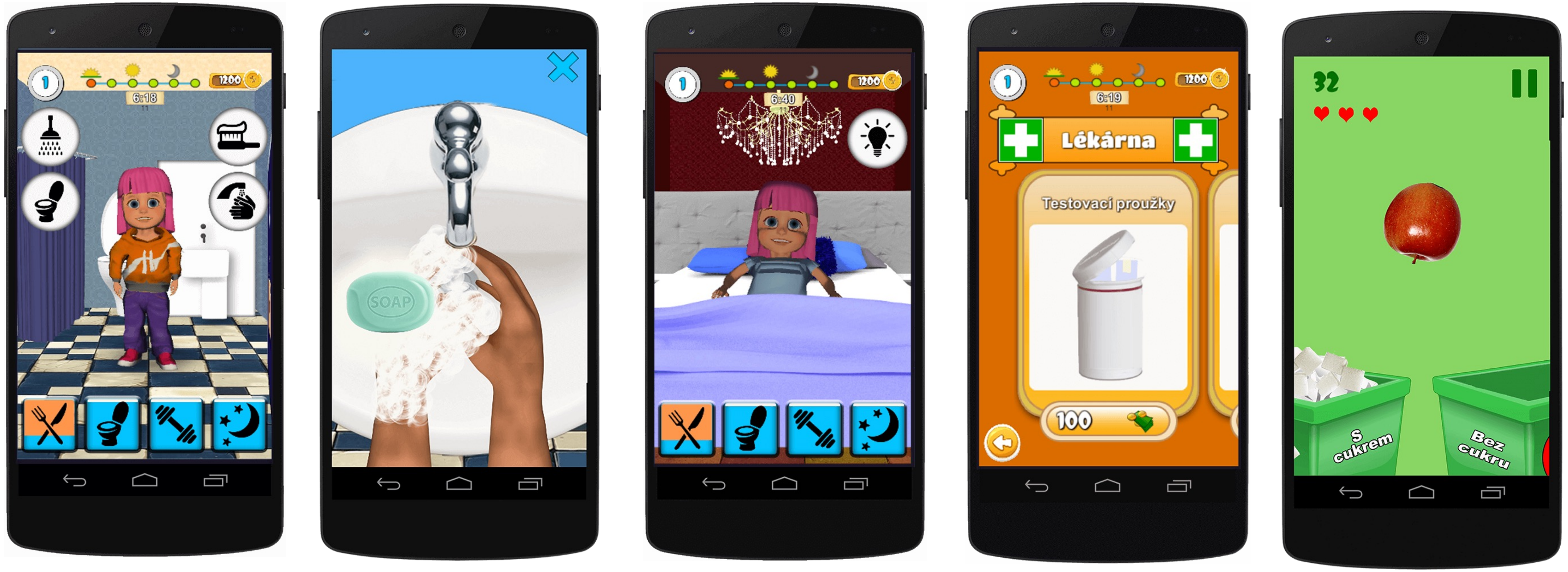

Content

Intro
Gamification
Some games
Goals
Concept
Features
What next
Thanks

Some stats

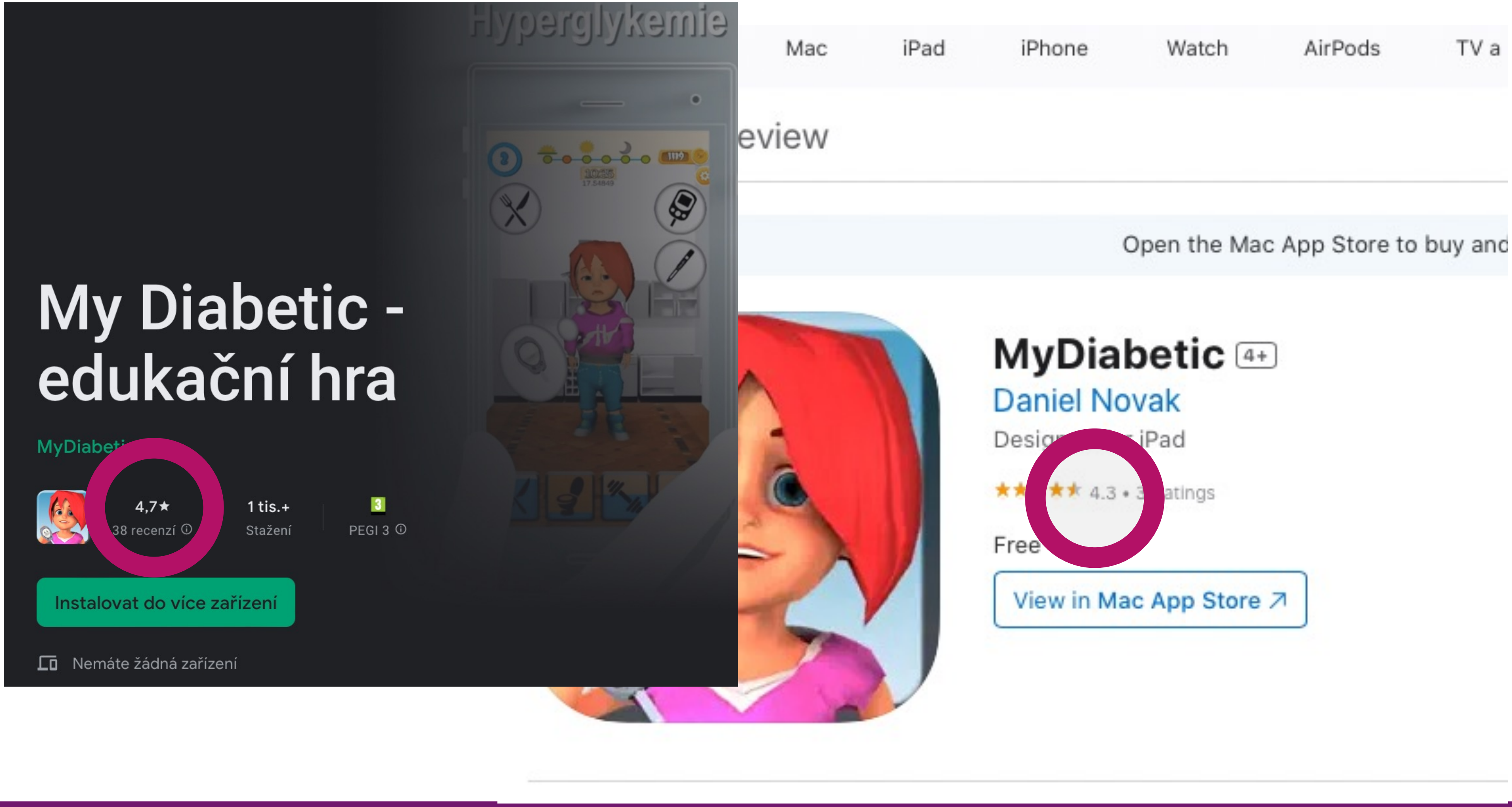

Content

Intro
Gamification
Some games
Goals
Concept
Features
Stats
Thanks

Some stats

Users by Country

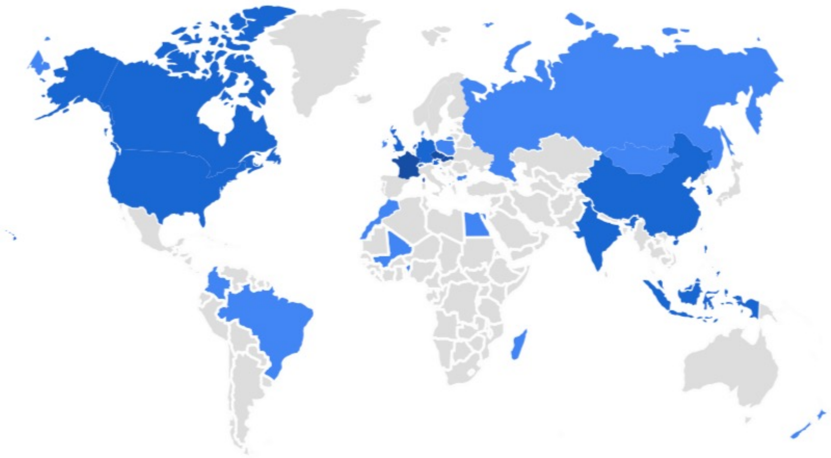

COUNTRY	USERS
Czechia	347
Slovakia	147
France	119
United States	63
Germany	8
South Korea	6
Netherlands	5

Content

Intro
Gamification
Some games
Goals
Concept
Features
Stats
Thanks

Some stats

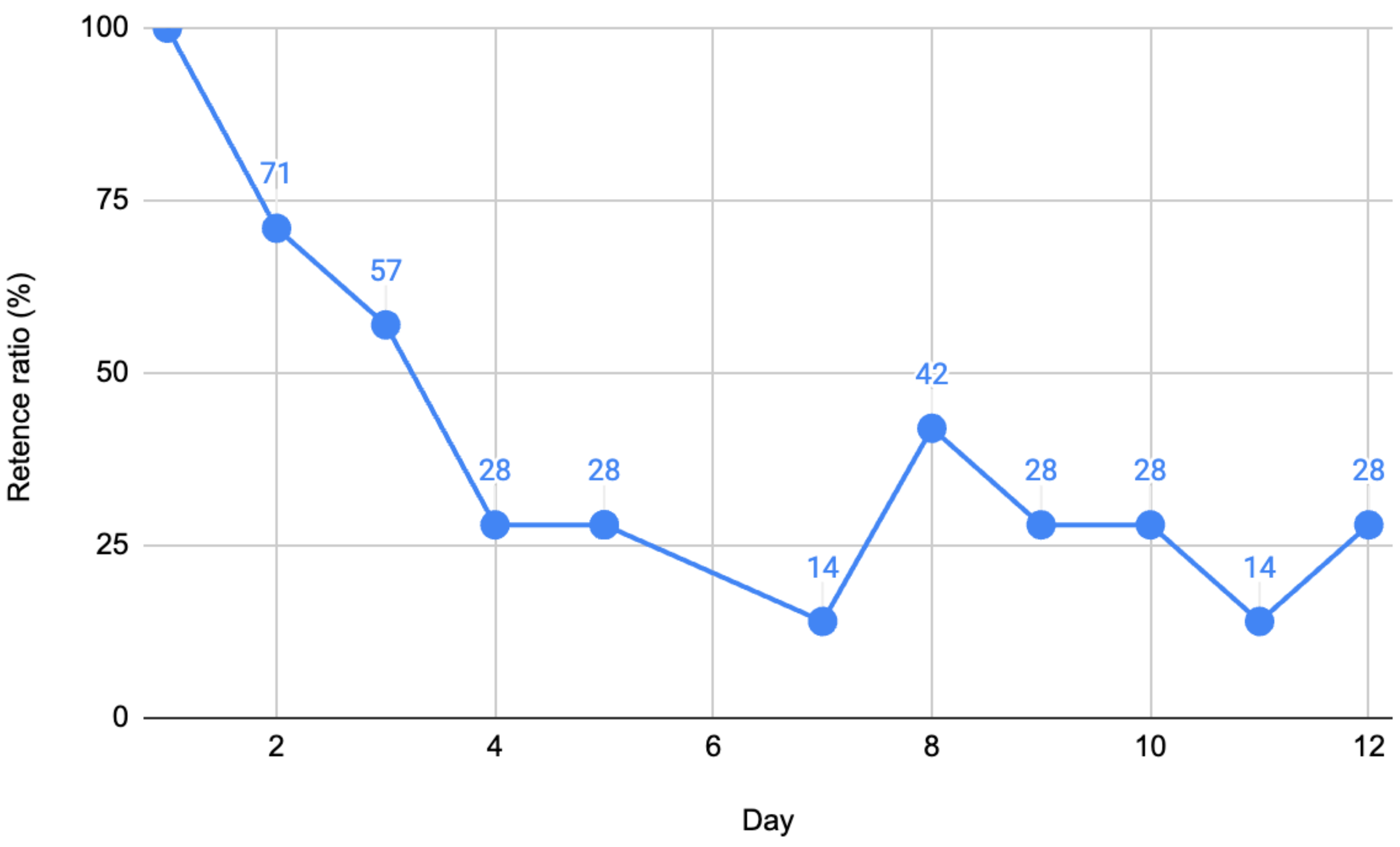

Content

Intro
Gamification
Some games
Goals
Concept
Features
Stats
Thanks

Some stats

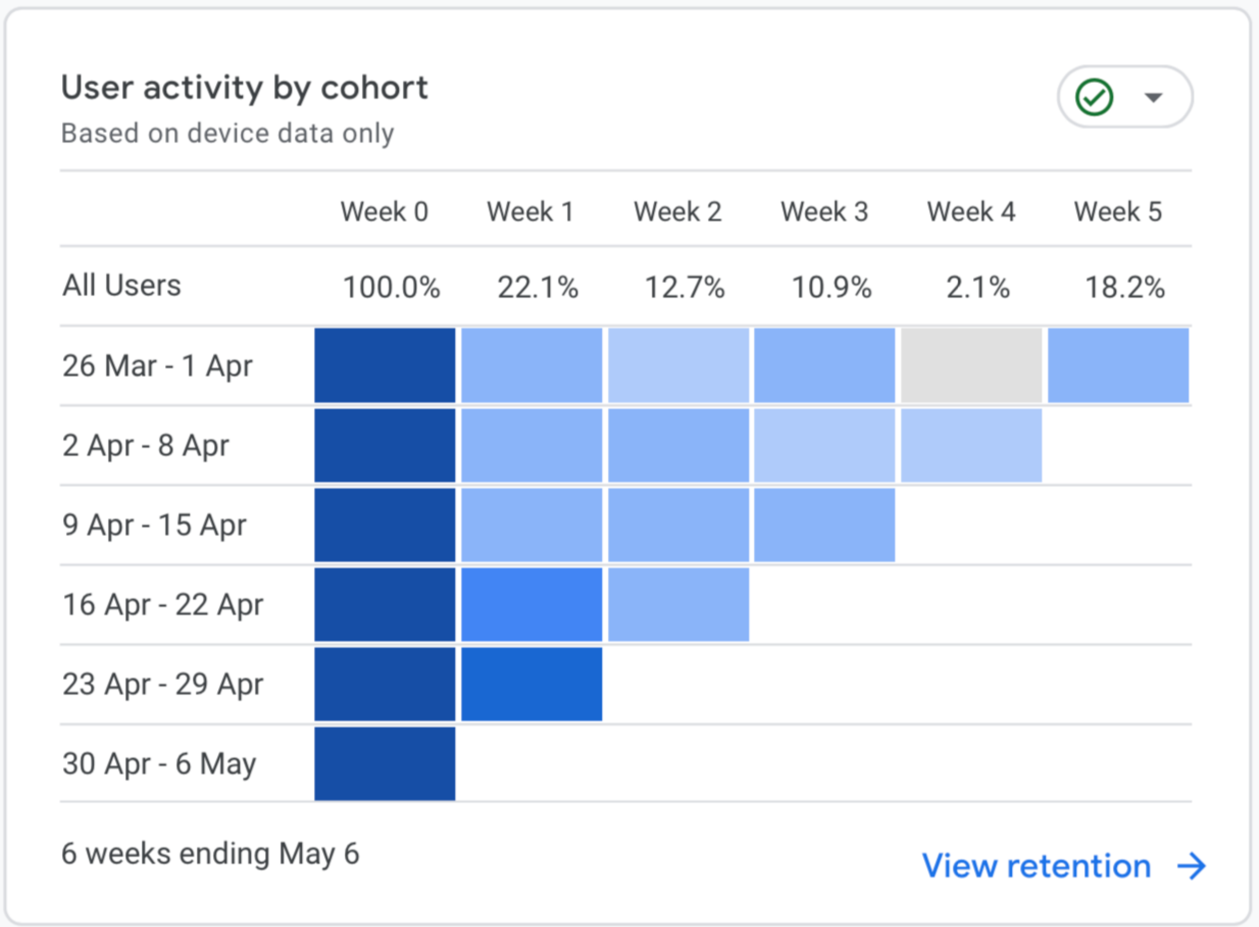

Content

Intro
Gamification
Some games
Goals
Concept
Features
Stats
Thanks

Story lines concept

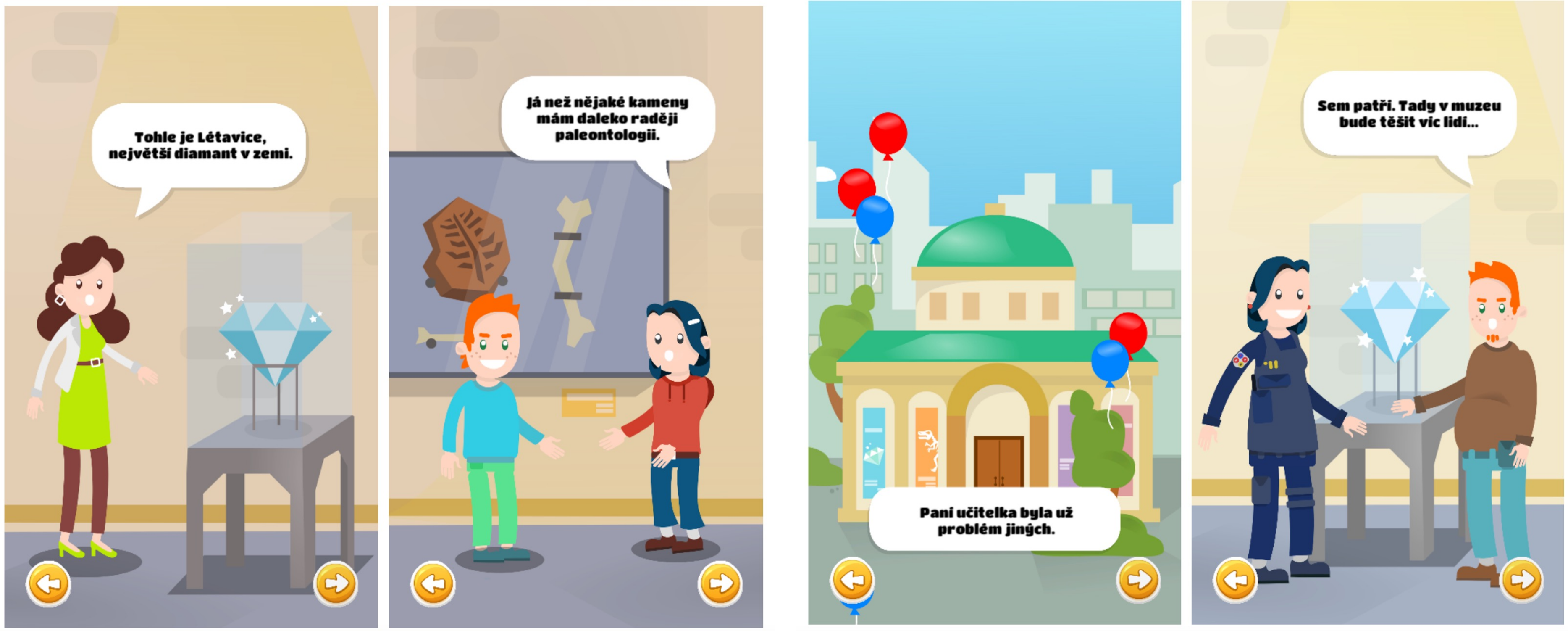

Content

- Intro
- Gamification
- Some games
- Goals
- Concept
- Features
- What next
- Thanks

Complications

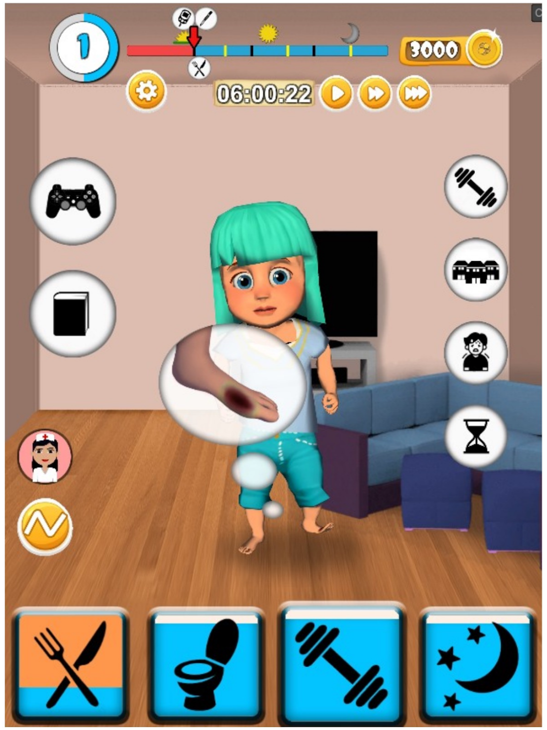

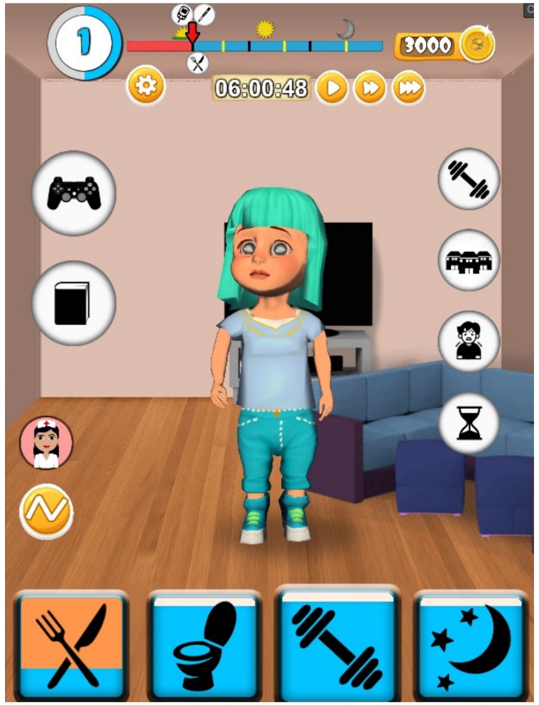

Content

Intro
Gamification
Some games
Goals
Concept
Features
What next
Thanks

Conclusion

- CHILDREN AND PARENTS LIKE THE GAME
- ADHERENCE IS AVERAGED COMPARED TO LITERATURE
- FURTHER DEVELOPMENT TO INCREASE ADHERENCE
  - Big competition from game industry
- OBSERVATION STUDY IN CLINICAL SETTINGS
  - Educational effect, compensation using e.g HbA1c?
- MEASURING EDUCATIONAL EFFECT
  - Significant improvements

Content

- Intro
- Gamification
- Some games
- Goals
- Concept
- Features
- What next
- Thanks

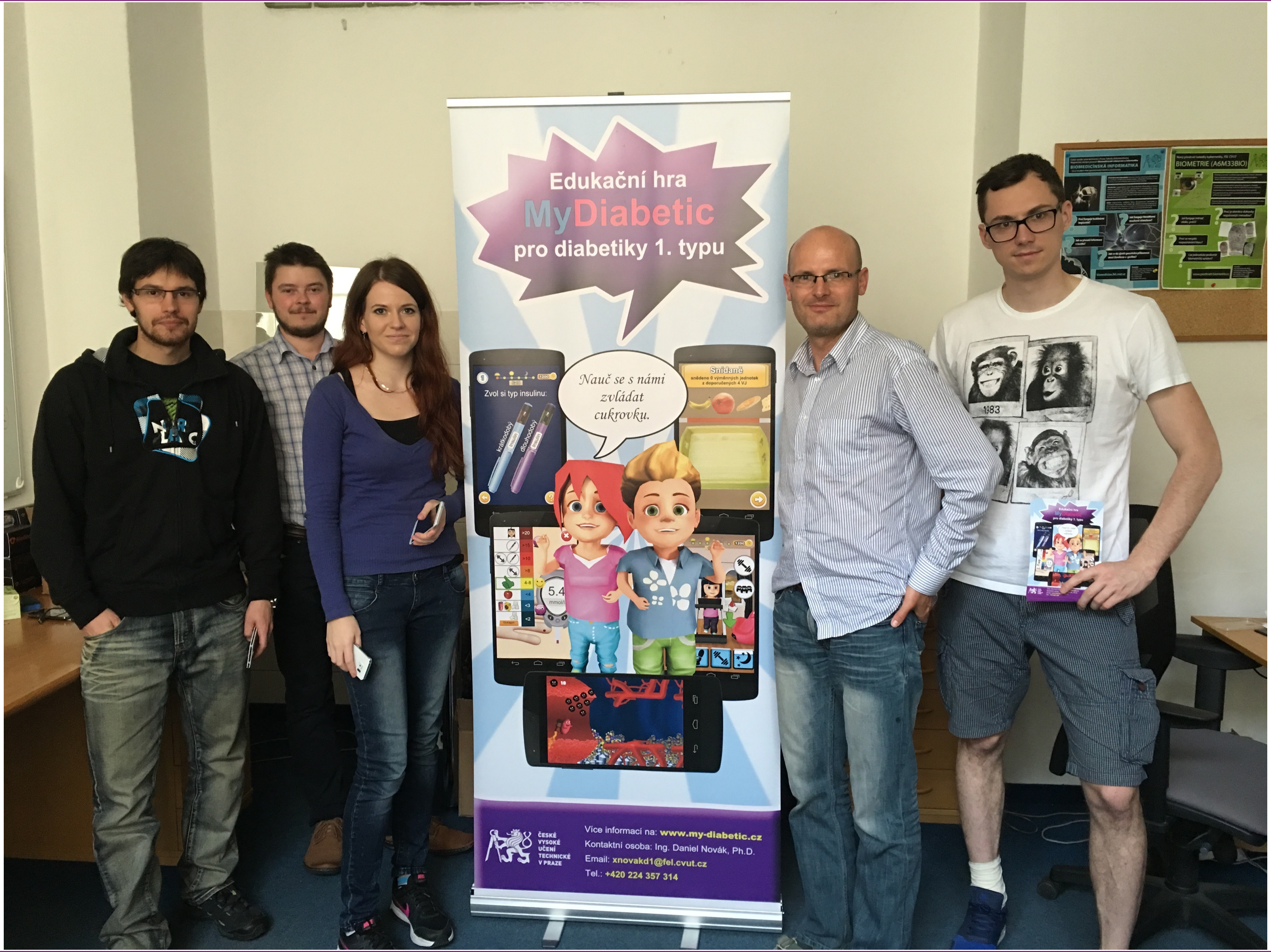

Content

Intro
Gamification
Some games
Goals
Concept
Features
What next
Thanks

Thanks to

- Z. SUMNIK, L. BRAZDOVA, K. STECHOVA, J. SKVOR, M. MRAZ
- V. LETOCHA, S. NOSALKOVA, Z. STANKOVA
- M. BERLINGER, L. WORKOWA
- V. CERNOHORSKA, J. KEJVALOVA, N. ZUBKOVA, D. JENCIK, L. RUBES, V. EYKHMANN, E. ULIARIKOVA, P. SALFICKA, B. HEJL

Content

- Intro
- Gamification
- Some games
- Goals
- Concept
- Features
- What next
- Thanks
